# Semi-synthesis and insecticidal activity of spinetoram J and its D-forosamine replacement analogues

**DOI:** 10.3762/bjoc.14.207

**Published:** 2018-09-04

**Authors:** Kai Zhang, Jiarong Li, Honglin Liu, Haiyou Wang, Lamusi A

**Affiliations:** 1 School of Chemistry and Chemical Engineering, Beijing Institute of Technology, 5 South Zhongguancan Street, Haidian District, Beijing, P. R. China; 2 Institute of Grassland Research of CAAS, No. 120 Wulanchabu East Street, Saihan District, Hohhot, P. R. China

**Keywords:** analogues, forosamine replacement, improved semi-synthesis, insecticide, natural products, spinetoram

## Abstract

Spinetoram, a mixture of spinetoram J (XDE-175-J, major component) and spinetoram L (XDE-175-L), is a new kind of fermentation-derived insecticide with a broad range of action against many insect pests, especially Cydia pomonella, Leaf miner and Thrips. Similar to spinosad, spinetoram is friendly to the environment, and non-toxic to animals and human beings. Therefore, spinetoram has been widely applied in pest control and grain storage. In a previous study, we had reported a semi-synthesis of spinetoram J. However, in that synthesis, there were more experimental steps, and the operations were troublesome. So an improved synthesis based on a self-protection strategy was designed and discussed. In this work, 3-*O*-ethyl-2,4-di-*O*-methylrhamnose was used as both the reaction substrate of C9–OH and the protecting group of C17–OH. The number of synthetic steps and costs were significantly reduced. In addition, a variety of D-forosamine replacement analogues of spinetoram J were synthesized based on the improved semi-synthesis, and their insecticidal activities were evaluated against third-instar larvae of *Plutella xylostella*. Although none of the analogues were as potent as spinetoram, a few of the analogues have only a 20–40 times lower activity than spinetoram. In particular, one of these analogues was approximately as active as spinosad. This study highlights the possibility of developing new insecticidal chemistries by replacing sugars on natural products with other groups, and the improved semi-synthesis will be helpful for further researches on spinetoram.

## Introduction

**Figure 1:**
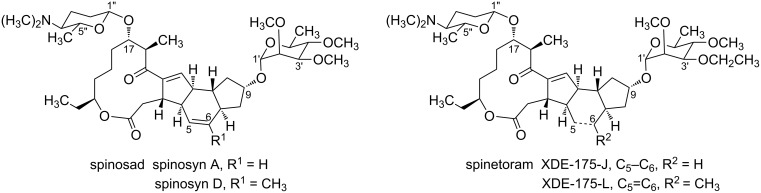
Structures of spinosad and spinetoram.

Nowadays, insect pests are one of the primary hazards that affect crop production and storage, and pest control depends mainly on the use of insecticides. Therefore, it is particularly important to identify an efficient insecticide for controlling insect pests. In the quest for new and efficient insecticides, natural products have always been considered an excellent source of inspiration for insecticides [1–2]. Among natural product-based insecticides, spinosyns are a novel kind of green insecticides with broad insecticide spectrum. Spinosyns, derivatives of bioactive substances produced by the soil actinomycete *Saccharopolyspora spinosa* [3], have been widely applied in pest control and crop protection due to their broad pest spectrum, high insecticidal activity, low environmental impact, and low toxicity to non-target species [4]. So far, more than 24 natural spinosyns and 800 semi-synthetic spinosyn analogues have been obtained and characterized, and their relative insecticidal activities have also been reported [5–6]. Since 1990, there are two generations of commercial products of spinosyns (spinosad) and spinetoram (Figure 1). Structurally, spinosad and spinetoram are both composed of D-forosamine and rhamnose coupled to a macrocyclic tetracycle. Spinosad, a mixture of ≈85% spinosyn A and ≈15% spinosyn D [7], is considered a highly effective bioinsecticide, and it has been widely used for the management of various insect pests. Compared with spinosad, spinetoram is more active and has a longer duration of control against many insect pests, such as codling moth (Cydia pomonella), a major pest of pome fruits, tobacco budworm, cotton and vegetable crops [8]. Like spinosad, spinetoram elicits toxicity in the pest species via a neurotoxic mode of action. Although the exact mechanism of action has yet to be characterized, it is hypothesized that the spinosyn molecule interacts with both gamma-aminobutyric acid (GABA) receptor and nicotine acetylcholine (NACh) receptors [9]. Spinosad and spinetoram can be degraded via a combination of photolysis and microbial action, ultimately producing CO_2_, H_2_O, and nitrogen oxides [10]. That suggests that along with a broad-pest spectrum and crop-uses, spinosad and spinetoram are both environment- and human-friendly. In addition, spinosad and spinetoram show high specificity to target insects and low toxicity to both mammals and beneficial insects [11]. Therefore, spinosad and spinetoram were successively awarded the Presidential Green Chemistry Challenge Award in 1999 and 2008 [12].

**Scheme 1:**
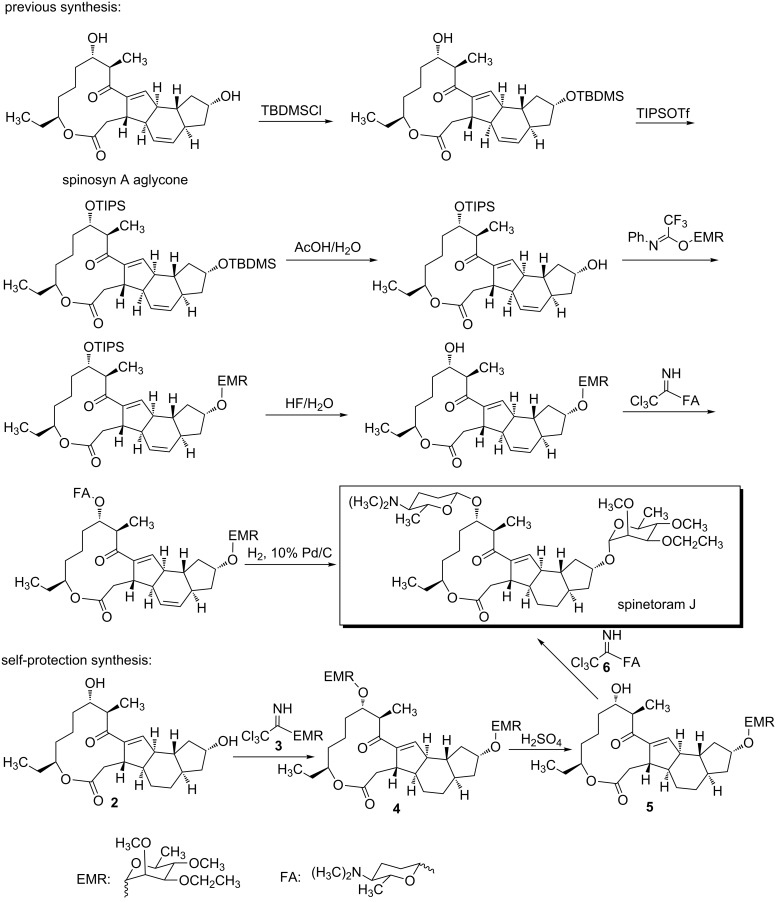
Comparison of previous synthesis and self-protection synthesis.

**Scheme 2:**
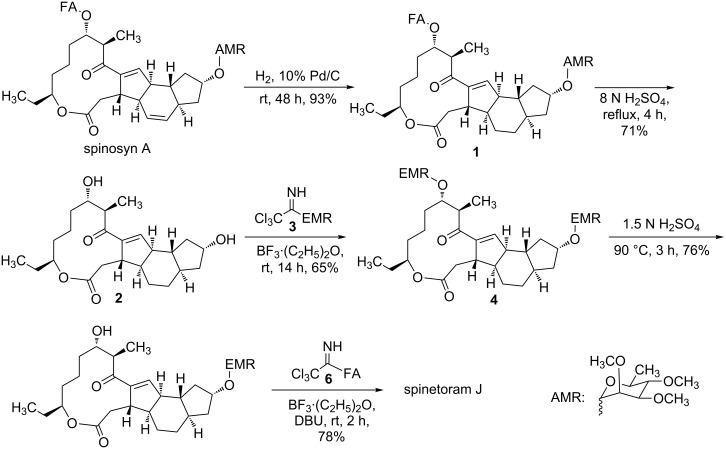
Synthesis of spinetoram J from spinosyn A based on a self-protection strategy.

At present, spinetoram is obtained from spinosyn J and spinosyn L by a chemical modification. However, the fermentation productivity of spinosyn J and spinosyn L is low. So a chemical synthesis of spinetoram would be helpful for the application of spinetoram, as well as for the further research on spinetoram. In the previous study [13], we had reported a semi-synthesis of spinetoram J with spinosyn A aglycon as initiator (Scheme 1). However, in the previous route, C9–OH and C17–OH of the aglycone needed to be protected with different protecting groups, resulting in cumbersome steps and high costs. To reduce the synthetic steps and simplify the operations, we developed a new semi-synthesis of spinetoram J based on a self-protection strategy (Scheme 2). In this study, 3-*O*-ethyl-2,4-di-*O*-methylrhamnose was used not only as the glycosylation donor of C9–OH, but also the protecting group of C17–OH, greatly reducing the synthetic steps and costs.

**Scheme 3:**
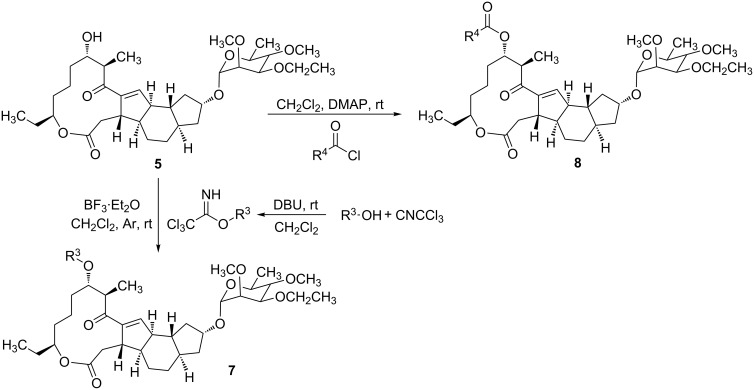
Synthesis of spinetoram J analogues.

Macrolide compounds are a new kind of insecticides and fungicides which have also been widely applied in medicine [14–15]. Currently, research on structural modification of macrolide compounds, such as modification of the macrolide and the branched chain glycosyl residue, and replacement of the sugar group, are in the limelight [16–17]. The unique macrolide structure of spinosyns and the efficient appearance of resistance in some insect pests have prompted further exploration of spinosyns via chemical modification [18]. Bioactivities of many microbial secondary metabolites are highly dependent on their sugar constituents which are transferred as nucleotide-activated sugars to an aglycon by glycosyltransferases [19]. Therefore, bioactivities of these metabolites could change when the sugar constituents are altered. Previous studies have shown that modification of the spinosyn structure could potentially improve insecticidal activity and expand the insect spectrum [20–21]. Indeed, a large number of researchers have altered spinosad, replacing rhamnose or D-forosamine groups with sugar and nonsugar substituents [22–26], and evaluated the insecticidal efficacy of these new spinosyn products. Although most spinosyn analogues were not as potent as spinosyns, a few analogues demonstrated good potential for the use in pest control. However, up until now, there were no reports about the chemical modification of spinetoram. Therefore, in this study, a series of D-forosamine replacement analogues of spinetoram J were synthesized based on the improved semi-synthesis (Scheme 3), and the insecticidal activities of analogues were evaluated against third-instar larvae of *Plutella xylostella*. This work extended the examination of sugar and nonsugar substituents to determine whether simple sugar or nonsugar substitutions would be suitable bioisosteric substituents for the D-forosamine sugar of spinetoram J.

## Results and Discussion

### Semi-synthesis of spinetoram J

As reported in the previous study [27], spinosyn A could be hydrolyzed to 17-pseudoaglycone under weak sulfuric acid conditions, indicating that the C9 glycosidic bond was more stable than the C17 glycosidic bond under weak sulfuric acid conditions (Scheme 2). Similar to spinosyn A, both D-forosamine and 2’,3’,4’-tri-*O*-methylrhamnose of 5,6-dihydrospinosyn A could be hydrolyzed under strong acidic conditions to afford the aglycone of spinetoram J (compound **2**). Then compound **2** was glycosylated with donor **3** to provide **4**. Since the C9 glycosidic bond was more stable than the C17 glycosidic bond under weak sulfuric acid conditions, the 3-*O*-ethyl-2,4-di-*O*-methylrhamnose at the C17 position could be removed selectively under the weak acidic conditions to afford **5**. The structure of compound **5** was also ascertained by NMR and mass spectrometry. Finally, compound **5** was glycosylated with donor **6** to provide spinetoram J. Compared with the previous semi-synthesis, selective protection and deprotection of the C9–OH and the C17–OH were simplified in this self-protection strategy, resulting in less synthetic steps and costs.

During the selective hydrolysis of 3-*O*-ethyl-2,4-di-*O*-methylrhamnose at the C17 position, neither the 9-pseudoaglycone of compound **4** nor the aglycone (compound **2**) were observed. Moreover, the analogues **7** and **8** were also hydrolyzed under 1.5 N H_2_SO_4_ at 90 °C, and the hydrolysis yields of all analogues were shown in Table 1. During the hydrolysis of analogues **7**, only the 17-pseudoaglycone of spinetoram J (compound **5**) was observed, which was the same as the hydrolysis of compound **4**. However, during the hydrolysis of analogues **8**, none of the 17-pseudoaglycone of spinetoram J, the 9-pseudoaglycone of compound **4**, nor compound **2** were observed. It seemed that the ester bond is more stable than the ether bond at the C17 position under acidic conditions.

**Table 1:** The hydrolysis yields of spinetoram J analogues under weak acidic conditions.

compound	C17–O substituent structure	hydrolysis yield

**7a**	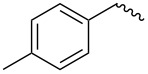	56%
**7b**	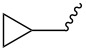	54%
**7c**	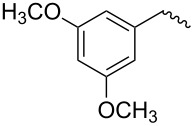	53%
**7d**	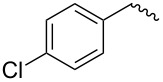	55%
**7e**	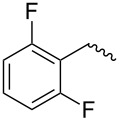	53%
**7f**	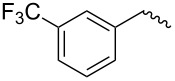	57%
**7g**	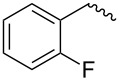	56%
**7h**	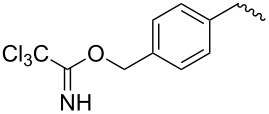	61%
**7i**	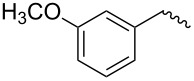	59%
**7j**	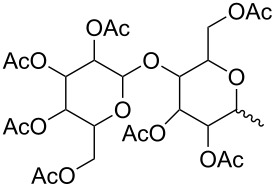	57%
**7k**	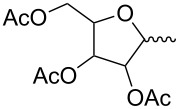	62%
**7l**	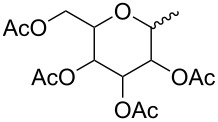	63%
**8a**	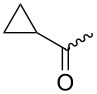	0
**8b**	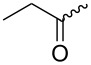	0
**8c**	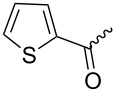	0
**8d**	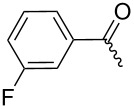	0
**8e**	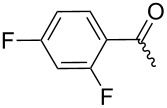	0
**8f**	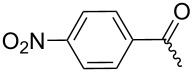	0
**8g**	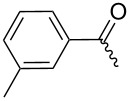	0
**8h**	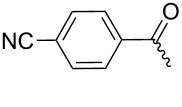	0
**8i**	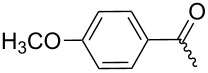	0

### Synthesis of spinetoram J analogues

All carbohydrates and alcohols were activated by CNCCl_3_ with DBU as catalyst initially to afford glycoside donors, and then the 17-pseudoaglycone of spinetoram J was glycosylated with donors in the presence of BF_3_·(C_2_H_5_)_2_O under Ar gas. Due to the high yields and few byproducts in the synthesis of glycoside donors, the donors could be used directly in the glycosylation without purification.

### Insecticidal activity

The insecticidal activities of synthetic spinetoram J and its analogues were evaluated using third-instar larvae of *P. xylostella*. The lethal concentration for 50% of the test population (LC_50_) and 95% confidence intervals were corrected for mortality in the controls, and then determined using *Probit* analysis. The results are shown in Table 2.

**Table 2:** The insecticidal activities of spinetoram J and its analogues.

compound	LC_50_ (mg/L)	95% CI (mg/L)

spinosad [28]	0.627	0.509–0.771
spinetoram [28]	0.048	0.036–0.060
spinetoram J	0.052	0.043–0.061
**7a**	14.621	10.824–23.019
**7b**	8.654	7.538–10.016
**7c**	2.151	1.651–3.061
**7d**	1.577	1.272–2.015
**7e**	1.792	1.428–2.350
**7f**	2.557	2.062–3.376
**7g**	1.226	1.047–1.440
**7h**	10.172	8.136–13.585
**7i**	7.342	6.155–8.964
**7j**	2.166	1.844–2.545
**7k**	2.537	2.164–2.986
**7l**	3.824	3.225–4.516
**8a**	16.353	11.659–28.038
**8b**	0.805	0.742–1.051
**8c**	18.097	14.934–22.689
**8d**	17.633	15.591–20.221
**8e**	16.602	13.464–21.413
**8f**	13.895	11.375–17.604
**8g**	21.996	16.964–31.458
**8h**	9.724	8.049–11.825
**8i**	12.792	10.577–15.810

As shown in Table 2, the semi-synthetic spinetoram J exhibited excellent insecticidal activity against *P. xylostella*, which was similar to spinetoram. The results prompted a comprehensive evaluation of spinetoram J analogues, which indicated that spinetoram J analogues had a wide range of insecticidal activity. Indeed, all of the spinetoram J analogues demonstrated toxicity to *P. xylostella*. Although most analogues were far less toxic than spinetoram (demonstrating 50−400 times lower toxicity), but a few of the analogues, such as **7d**, **7e**, **7g** and **8b**, exhibited toxicity to *P. xylostella* that was 20–40 times lower than that of spinetoram. Particularly, the compound **8b** was approximately as active as spinosad.

Carbohydrate substituents are considered to be related closely to many secondary metabolites [19], hence, C17–O glycosyl analogues are regarded as efficient insecticides that are likely to rival present insecticides. However, as noted in Table 2, C17–O-glycosyl analogues **7j**, **7k** and **7l** did not exhibit excellent performance as expected.

Similar to the C17–O-benzyl analogues of spinosyn A, the C17–O-benzyl analogues of spinetoram J with electron-withdrawing substituents demonstrated better insecticidal activity than the C17–O-benzyl analogue with electron-donating substituents, illustrating that the insecticidal activities of spinetoram J analogues are closely related to the electrical properties of the substituents. For compound **7c**, the methoxy group was attached to the benzene ring at the *meta*-position, leading to a decrease in the conjugate effect, mainly for the role of electron absorption. So the compound **7c** exhibited much more efficient insecticidal activity than the C17–O-benzyl analogues with electron-donating substituents. Therefore, an electron-donating substituent is more likely to generate a promising analogue with good insecticidal activity. In addition, the majority of C17–O-acyl analogues did not show as good insecticidal activity as other analogues, probably indicating that C17–O-ether analogues were more likely to show more efficient insecticidal activity than C17–O-ester analogues.

Spinetoram has excellent insecticidal activity against a broad array of target pests such as Lepidopteran, Thysanoptera, Diptera, Isoptera, Coleoptera and Themiptera [29]. So the spinetoram J analogues may be potent against other pests in addition to *P. xylostella*, such as leafroller thrips, leafminer flies, white flies and so on. Moreover, these analogues may also have antibiotic, antifungal, anticancer, antiparasitic, and immunosuppressive properties.

## Conclusion

This study demonstrated a viable approach to synthesize spinetoram J and its analogues. Compared with the previous semi-synthesis of spinetoram J, the improved semi-synthesis shown in this study demonstrated less steps and simpler operations. Meanwhile, a variety of spinetoram J analogues were synthesized based on this improved semi-synthesis, and their insecticidal activities were evaluated against third-instar larvae of *P. xylostella*. Although spinetoram J analogues displayed a wide range of insecticidal activity, none of the analogues were as potent as spinetoram, but a few of the analogues exhibited insecticidal activity that was close to that of spinetoram. This study demonstrates a viable method to develop natural products that contain glycosidic bonds or hydroxy groups. It also highlights the potential for developing new insecticidal products by replacing sugars on natural products with other groups.

## Experimental

### Insecticide bioassays

The insecticidal activities of spinetoram J analogues were evaluated against third-instar larvae of *P. xylostella*. Stock solutions were prepared by weighting manufactured analogues and dissolving them in acetone. Dosing solutions were then prepared by diluting stock solutions to different concentrations with 0.01% Triton X-100 aqueous solution. Cabbage leaves were then soaked in the dosing solutions for 10 s, dried, and placed in Petri dishes. Approximately twenty *P. xylostella* were added to each dish. After 48 h, the number of *P. xylostella* deaths was recorded. Three replicates were used for each compound of exposure.

### Preparation of 5,6-dihydrospinosyn A (**1**)

Spinosyn A (3.96 g, 5.43 mmol) was added to 110 mL methanol, and then 10% Pd/C (0.36 g, 0.33 mmol) was added. The mixture was stirred under hydrogen at room temperature for 48 h. The mixture was then filtered. The filtrate was evaporated under reduced pressure, and the residue was purified by column chromatography on silica gel (200–300 mesh) to afford pure **1** (3.70 g, yield 93%). TLC (methanol/dichloromethane 1:9, v:v); ^1^H NMR (400 MHz, CDCl_3_) δ 6.79 (s, 1H, C_13_-H), 4.75 (s,1H, C_1’_-H), 4.58 (m, 1H, C_21_-H), 4.39 (d, *J =* 7.6 Hz, 1H, C_1”_-H), 4.13 (m, 1H, C_9_-H), 3.57 (m, 1H, C_2’_-H), 3.48–3.41 (m, 14H, C_5”_-H, C_17_-H, C_5’_-H, C_4_-H, C_4’_-OCH_3_, C_2’_-OCH_3_, C_3’_-OCH_3_, C_3’_-H), 3.20 (m, 1H, C_16_-H), 3.21–3.09 (m, 2H, one of C_2_-H, C_3_-H), 2.89–2.67 (m, 3H, C_4’_-H, C_12_-H, one of C_2_-H), 2.54–2.42 (m, 3H, one of C_10_-H, C_7_-H, C_4”_-H), 2.37 (m, 6H, N(CH_3_)_2_), 2.28–2.16 (m, 2H, one of C_8_-H, one of C_2”_-H), 1.96–1.93 (m, 3H, one of C_3”_-H, one of C_8_-H, one of C_19_-H), 1.59–1.40 (m, 10H, C_18_-H, one of C_20_-H, C_22_-H, one of C_2”_-H, one of C_3”_-H, one of C_5_-H, one of C_6_-H, one of C_10_-H), 1.31 (d, *J =* 7.6 Hz, 3H, C_6’_-H), 1.21–1.18 (m, 6H, C_16_-CH_3_, C_6”_-H), 1.11–1.09 (m, 4H, one of C_19_-H, one of C_20_-H, one of C_5_-H, one of C_6_-H), 0.96 (m, 1H, C_11_-H), 0.74 (t, *J =* 7.6 Hz, 3H, C_23_-H); ^13^C NMR (101 MHz, CDCl_3_) δ 202.21, 171.51, 148.53, 144.13, 102.03, 94.45, 81.29, 80.05, 79.45, 76.74, 74.57, 71.59, 66.87, 64.02, 59.91, 58.01, 57.99, 56.69, 49.03, 46.82, 45.54, 42.17, 39.99, 39.51, 38.49, 37.75, 37.00, 33.26, 31.98, 29.49, 28.94, 27.44, 26.01, 23.49, 20.89, 18.43, 18.39, 18.30, 16.80, 15.00, 8.36; MS (MALDI) *m*/*z*: [M + H]^+^ calcd for C_41_H_67_NO_10_, 734.483774; found, 734.483663.

### Preparation of aglycone of 5,6-dihydrospinosyn A **2**

Compound **1** (2.15 g, 3.64 mmol) was added to a solution of 60 mL MeOH and 130 mL 8 N H_2_SO_4_, then the mixture were heated to reflux for 4 hours. After the mixture was cooled, an appropriate amount of NaHCO_3_ was added to adjust the pH to 5–6. Then the mixture was extracted with CH_2_Cl_2_ (3 **×** 15 mL). The CH_2_Cl_2_ extracts were combined, dried with Na_2_SO_4_ and evaporated under reduced pressure. The residue was purified by column chromatography on silica gel (200–300 mesh) to afford pure **2** (1.04 g, yield 71%). TLC (ethyl acetate/petroleum ether 2:1, v:v); ^1^H NMR (400 MHz, CDCl_3_) δ 6.84 (s, 1H, C_13_-H), 4.60 (m, 1H, C_21_-H), 4.27 (m, 1H, C_9_-H), 3.58 (m, 1H, C_17_-H), 3.16–3.06 (m, 2H, C_16_-H, one of C_2_-H), 2.89 (m, 1H, C_3_-H), 2.75 (m, 1H, C_12_-H), 2.51 (m, 1H, one of C_2_-H), 2.27–2.22 (m, 2H, one of C_8_-H, C_7_-H), 1.62–1.19 (m, 13H, C_18_-H, C_20_-H, C_22_-H, one of C_10_-H, C_5_-H, C_6_-H, one of C_8_-H, one of C_19_-H), 1.14 (d, *J =* 3.4 Hz, 3H, C_16_-CH_3_), 1.10 (m, 1H, C_4_-H), 0.94 (m, 1H, C_11_-H), 0.75 (t, *J* = 7.4 Hz, 3H, C_23_-H); ^13^C NMR (101 MHz, CDCl_3_) δ 202.53, 171.70, 148.75, 144.38, 71.25, 70.50, 52.47, 49.11, 47.28, 46.21, 42.04, 41.23, 40.26, 40.06, 38.50, 33.95, 31.75, 28.83, 27.46, 26.05, 23.45, 21.13, 14.64, 8.39; MS (MALDI) *m*/*z*: [M + Na]^+^ calcd for C_24_H_36_O_5_, 427.245495; found, 427.245611.

### Preparation of 3-ethoxy-2,4-dimethoxyrhamnosyltrichoroacetimidate (**3**)

3-*O*-Ethyl-2,4-di-*O*-methylrhamnose (0.91 g, 4.09 mmol) was dissolved in 20 mL CH_2_Cl_2_, then CNCCl_3_ (0.81 g, 5.61 mmol) and 0.15 mL DBU was added. The mixture was stirred at room temperature for 20 min, then diluted with CH_2_Cl_2_ and washed with saturated sodium bicarbonate solution (3 × 10 mL). The combined organic layers were dried with Na_2_SO_4_ and evaporated under reduced pressure. The residue can be used directly in the next reaction step.

### Preparation of spinetoram J analogue **4**

Compound **2** (0.32 g, 0.79 mmol) and compound **3** (0.68 g, 1.87 mmol) were dissolved in 10 mL dry CH_2_Cl_2_ with some molecular sieve under Ar. Then BF_3_·(C_2_H_5_)_2_O (0.12 mL, 1.01 mmol) was added at room temperature. The mixture was stirred for 14 h, then diluted with CH_2_Cl_2_ (15 mL) and washed with saturated sodium bicarbonate solution (3 × 10 mL). The combined organic layers were dried with Na_2_SO_4_ and evaporated under reduced pressure. The residue was purified by column chromatography on silica gel (200–300 mesh) to afford pure **4** (0.42 g, yield 65%). TLC (ethyl acetate/petroleum ether 1:2, v:v); ^1^H NMR (400 MHz, CDCl_3_) δ 6.79 (s, 1H, C_13_-H), 4.80–4.73 (m, 2H, C_1’_-H, C_1”_-H), 4.57 (m, 1H, C_21_-H), 4.20 (m, 1H, C_9_-H), 3.73–3.52 (m, 16H, C_2’_-H, C_2”_-H, C_17_-H, C_3’_-OCH_2_-, C_3”_-OCH_2_-, C_4’_-OCH_3_, C_4”_-OCH_3_, C_5’_-H, C_5”_-H, C_4_-H), 3.48–3.44 (m, 8H, C_2’_-OCH_3_, C_2”_-OCH_3_, C_4’_-H, C_4”_-H), 3.22 (m, 1H, C_10_-H), 3.11–2.99 (m, 5H, one of C_2_-H, C_3_-H, C_3’_-H, C_3”_-H, C_12_-H), 2.36–2.26 (m, 2H, one of C_2_-H, C_7_-H), 2.18 (m, 2H, C_10_-H), 1.88 (m, 1H, one of C_8_-H), 1.61–1.37 (m, 11H, C_5_-H, C_6_-H, one of C_8_-H, C_18_-H, one of C_19_-H, one of C_20_-H, C_22_-H), 1.28–1.22 (m, 7H, one of C_20_-H, C_6’_-H, C_6”_-H), 1.18–1.12 (m, 10H, one of C_19_-H, C_16_-CH_3_, C_3’_-OC-CH_3,_ C_3”_-OC-CH_3_), 0.95 (m, 1H, C_11_-H), 0.75 (t, *J* = 7.4 Hz, 3H, C_23_-H); ^13^C NMR (176 MHz, CDCl_3_) δ 202.76, 171.18, 149.17, 144.80, 99.43, 95.79, 82.19, 82.01, 79.68, 79.54, 78.51, 78.39, 75.70, 72.70, 68.68, 67.96, 65.68, 61.02, 60.41, 59.23, 59.02, 50.26, 48.19, 47.07, 46.37, 43.52, 40.79, 39.60, 38.80, 38.14, 34.86, 34.22, 31.59, 30.62, 28.05, 26.94, 24.68, 22.66, 17.85, 17.72, 15.76, 15.69, 14.20, 9.41; MS (MALDI) *m*/*z*: [M + Na]^+^ calcd for C_44_H_72_O_13_, 831.486513; found, 831.486515.

### Preparation of spinetoram J 17-pseudoaglycone **5**

Compound **4** (0.23 g, 0.28 mmol) was dissolved in 10 mL 1.5 N H_2_SO_4_, then the mixture was heated to 90 °C. After 3 h of stirring, the mixture was cooled to room temperature, and then extracted with CH_2_Cl_2_ (3 × 15 mL). The CH_2_Cl_2_ extracts were combined, dried with Na_2_SO_4_ and evaporated under reduced pressure. The residue was purified by column chromatography on silica gel (200–300 mesh) to afford pure **5** (0.13 g, yield 76%). TLC (ethyl acetate/petroleum ether 1:1, v/v); ^1^H NMR (400 MHz, CDCl_3_) δ 6.87 (s, 1H, C_13_-H), 4.80 (s, 1H, C_1’_-H), 4.68 (m, 1H, C_21_-H), 4.21 (m, 1H, C_9_-H), 3.72 (m, 1H, C_2’_-H), 3.62 (m, 1H, C_17_-H), 3.57–3.56 (m, 5H, C_3’_-OCH_2_-, C_4’_-OCH_3_), 3.54 (m, 1H, C_5’_-H), 3.51 (m, 1H, C_4_-H), 3.49 (s, 3H, C_2’_-OCH_3_), 3.44 (m, 1H, C_4’_-H), 3.19–3.11 (m, 3H, C_16_-H, one of C_2_-H, C_3_-H), 2.95 (m, 1H, C_3’_-H), 2.82 (m, 1H, C_12_-H), 2.36 (dd, *J*_1_ = 13.2 Hz, *J*_2_ = 2.8 Hz, 1H, one of C_2_-H), 2.32 (m, 1H, C_7_-H), 2.25 (m, 2H, C_10_-H), 1.93 (m, 1H, one of C_8_-H), 1.73–1.47 (m, 11H, C_5_-H, C_6_-H, one of C_8_-H, C_18_-H, one of C_19_-H, one of C_20_-H, C_22_-H), 1.28–1.21 (m, 11H, one of C_19_-H, one of C_20_-H, C_6’_-H, C_16_-CH_3_, C_3’_-OC-CH_3_), 1.01 (m, 1H, C_11_-H), 0.82 (t , *J* = 7.4 Hz, 3H, C_23_-H); ^13^C NMR (101 MHz, CDCl_3_) δ 203.24, 172.71, 149.44, 145.42, 95.80, 82.22, 79.69, 78.55, 75.55, 72.48, 67.98, 65.60, 61.02, 59.22, 50.03, 48.21, 46.59, 45.90, 43.17, 41.06, 39.51, 38.80, 38.01, 34.96, 32.84, 29.90, 28.47, 27.04, 24.50, 22.03, 17.85, 15.76, 15.60, 9.39; MS (MALDI) *m*/*z*: [M + Na]^+^ calcd for C_34_H_54_O_9_, 629.366004; found, 629.366428.

### Preparation of spinetoram J

Compound **5** (0.15 g, 0.25 mmol) and compound **6** (0.11 g, 0.36 mmol) were dissolved in 10 mL dry CH_2_Cl_2_ with some molecular sieve under Ar. Then BF_3_·(C_2_H_5_)_2_O (0.13 mL, 1.02 mmol) was added at room temperature. The mixture was stirred for 16 h. The mixture was then diluted with CH_2_Cl_2_ (15 mL) and washed with saturated sodium bicarbonate solution (3 × 10 mL). The combined organic layers were dried with Na_2_SO_4_ and evaporated under reduced pressure. The residue was purified by column chromatography on silica gel (200–300 mesh) to afford pure spinetoram J (0.15 g, yield 78%). TLC (methanol/dichloromethane 1:8, v/v); MS (MALDI) *m*/*z*: [M + Na]^+^ calcd for C_42_H_69_NO_10_, 770.481368; found, 770.481016.

### Preparation of **7a**

Compound **5** (0.32 g, 0.53 mmol) and 4-methylbenzyl-2,2,2-trichloroacetimidate (0.28 g, 0.45 mmol) were dissolved in 15 mL dry CH_2_Cl_2_ with some molecular sieve under Ar. Then BF_3_·(C_2_H_5_)_2_O (0.15 mL, 1.03 mmol) was added at room temperature. The mixture was stirred for 24 h, then diluted with CH_2_Cl_2_ (15 mL) and washed with saturated sodium bicarbonate solution (3 × 10 mL). The combined organic layers were dried with Na_2_SO_4_ and evaporated under reduced pressure. The residue was purified by column chromatography on silica gel (200–300 mesh) to afford pure **7a** (0.27 g, yield 72%). TLC (ethyl acetate/petroleum ether 1:2, v/v). ^1^H NMR (400 MHz, CDCl_3_) δ 7.25–7.13 (m, 4H, C_3”_-H, C_5”_-H, C_6”_-H, C_7”_-H,), 6.86 (s, 1H, C_13_-H), 4.81 (s, 1H, C_1’_-H), 4.65 (m, 1H, C_21_-H), 4.52 (m, 2H, C_1”_-H), 4.22 (m, 1H, C_9_-H), 3.73 (m, 1H, C_2’_-H), 3.64 (m, 1H, C_17_-H), 3.57–3.54 (m, 7H, C_3’_-OCH_2_-, C_4’_-OCH_3_, C_5’_-H, C_4_-H), 3.50 (s, 3H, C_2’_-OCH_3_), 3.45 (m, 1H, C_3’_-H), 3.17–3.09 (m, 3H, C_16_-H, one of C_2_-H, C_3_-H), 2.94 (m, 1H, C_4’_-H), 2.82 (m, 1H, C_12_-H), 2.59 (t, 3H, C_4”_-H), 2.35 (dd, 1H, one of C_2_-H), 2.31 (m, 1H, C_7_-H), 2.27 (m, 2H, C_10_-H), 1.94 (m, 1H, one of C_8_-H), 1.73–1.47 (m, 11H, C_5_-H, C_6_-H, one of C_8_-H, C_18_-H, one of C_19_-H, one of C_20_-H, C_22_-H), 1.31–1.25 (m, 11H, one of C_19_-H, one of C_20_-H, C_6’_-H, C_16_-CH_3_, C_3’_-OC-CH_3_), 1.01 (m, 1H, C_11_-H), 0.83 (t , 3H, C_23_-H); ^13^C NMR (101 MHz, CDCl_3_) δ 203.52, 172.59, 163.78, 149.16, 144.89, 137.32, 135.47, 129.05, 128.60, 127.99, 95.73, 82.22, 79.62, 78.61, 75.70, 71.94, 70.99, 67.97, 65.62, 60.92, 59.15, 50.28, 47.06, 46.33, 43.62, 40.78, 39.57, 38.98, 38.77, 38.14, 33.47, 31.93, 30.56, 28.05, 26.94, 24.71, 21.14, 20.84, 17.84, 15.73, 15.54, 9.36; MS (MALDI) *m*/*z*: [M + Na]^+^ calcd for C_42_H_62_O_9_, 733.428604; found, 733.428914.

### Preparation of **8a**

Compound **5** (0.33 g, 0.54 mmol), cyclopropanecarbonyl chloride (0.06 g, 0.58 mmol) and 4-dimethylaminopyridine (DMAP, 0.15 g, 1.23 mmol) were added to 15 mL CH_2_Cl_2_ under Ar, then the mixture was heated to reflux. After about 6 h, the mixture was diluted with CH_2_Cl_2_ (15 mL) and washed with saturated sodium bicarbonate solution (3 × 10 mL). The combined organic layers were dried with Na_2_SO_4_ and evaporated under reduced pressure. The residue was purified by column chromatography on silica gel (200–300 mesh) to afford pure **8a** (0.31 g, yield 85%). TLC (ethyl acetate/petroleum ether 1:3, v/v). ^1^H NMR (400 MHz, CDCl_3_) δ 6.75 (s, 1H, C_13_-H), 4.82 (s, 1H, C_1’_-H), 4.67 (m, 1H, C_21_-H), 4.50 (m, 1H, C_9_-H), 4.06 (m, 1H, C_2’_-H), 3.57 (m, 1H, C_17_-H), 3.46–3.31 (m, 12H, C_5’_-H, C_4_-H, C_3’_-OCH_2_-, C_2’_-OCH_3_, C_4’_-OCH_3_, C_3’_-H, C_16_-H), 3.23 (m, 1H, one of C_2_-H), 2.99–2.95 (m, 2H, C_3_-H, C_4’_-H), 2.80 (m, 1H, C_12_-H), 2.67 (m, 1H, one of C_2_-H), 2.43 (m, 2H, C_10_-H), 2.16 (m, 1H, C_7_-H), 1.94 (m, 1H, one of C_8_-H), 1.67 (m, 2H, one of C_5_-H, one of C_6_-H), 1.58 (m, 1H, C_2”_-H), 1.47–1.42 (m, 7H, one of C_8_-H, C_18_-H, one of C_19_-H, one of C_20_-H, C_22_-H), 1.34–1.32 (m, 5H, one of C_19_-H, one of C_20_-H, C_6’_-H), 1.20–1.10 (m, 8H, one of C_5_-H, one of C_6_-H, C_16_-CH_3_, C_3’_-OC-CH_3_), 0.97–0.90 (m, 5H, C_3”_-H, C_4”_-H, C_11_-H), 0.77 (m, 3H, C_23_-H); ^13^C NMR (101 MHz, CDCl_3_) δ 201.48, 174.28, 172.41, 149.15, 144.99, 95.71, 82.08, 79.48, 78.36, 76.27, 75.70, 74.66, 67.80, 65.39, 60.69, 58.90, 50.03, 49.04, 47.75, 46.34, 45.60, 43.20, 40.84, 39.44, 38.65, 37.94, 32.79, 32.34, 29.94, 28.04, 27.35, 26.83, 21.32, 17.57, 15.50, 12.57, 8.15, 8.01; MS (MALDI) *m*/*z*: [M + Na]^+^ calcd for C_38_H_58_O_10_, 697.392219; found, 697.392189.

## Supporting Information

File 1 Details for the synthesis of spinetoram J analogues, analytical data of all compounds, NMR spectra and MS data of all synthesized compounds.
