# Supplementary material for: Semi-synthesis and insecticidal activity of spinetoram J and its D-forosamine replacement analogues
Source: Beilstein J Org Chem. 2018 Sep 4;14:2321–30. doi: 10.3762/bjoc.14.207 (PMC6142758; doi:10.3762/bjoc.14.207)
Supplement: File 1 — Details for the synthesis of spinetoram J analogues, analytical data of all compounds, NMR spectra and MS data of all synthesized compounds. [file Beilstein_J_Org_Chem-14-2321-s001.pdf]

Supporting Information

for

Semi-synthesis and insecticidal activity of spinetoram J and its D-forosamine replacement analogues

Kai Zhang[1], Jiarong Li*[1], Honglin Liu[2], Haiyou Wang[2] and Lamusi A*[2]

Address: [1]School of Chemistry and Chemical Engineering, Beijing Institute of Technology, 5 South Zhongguancun Street, Haidian District, Beijing, P. R. China and [2]Institute of Grassland Research of CAAS, No. 120 Wulanchabu East Street, Saihan District, Hohhot, P. R. China

Email: Jiarong Li - jrligroup@126.com

*Corresponding author

Details for the synthesis of spinetoram J analogues, analytical data of all compounds, NMR spectra and MS data of all synthesized compounds

Experimental

Preparation of 7b

Compound **5** (0.36 g, 0.59 mmol) and cyclopropylmethyl-2,2,2-trichloroacetimidate (0.15 g, 0.71 mmol) were dissolved in 15 mL dry CH_2Cl_2 with some molecular sieve under Ar. Then $BF_3·(C_2H_5)_2O$ (0.13 mL, 1.02 mmol) was added at room temperature. The mixture was stirred for 22 h, then diluted with CH_2Cl_2 (15 mL) and washed with saturated sodium bicarbonate solution (3 × 10 mL). The combined organic layers were dried with Na_2SO_4 and evaporated under reduced pressure. The residue was purified by column chromatography on silica gel (200–300 mesh) to afford pure **7b** (0.23 g, yield 59%). TLC (ethyl acetate : petroleum ether = 1:2, v:v). ^{1}H NMR (400 MHz, $CDCl_3$) δ: 6.75 (s, 1H, C_{13}-H), 4.82 (s, 1H, $C_{1'}$-H), 4.67 (m, 1H, C_{21}-H), 4.50 (m, 1H, C_9-H), 4.06 (m, 1H, $C_{2'}$-H), 3.57 (m, 1H, C_{17}-H), 3.46-3.31 (m, 12H, $C_{5'}$-H, C_4-H, $C_{3'}$-OCH_2-, $C_{2'}$-OCH_3, $C_{4'}$-OCH_3, $C_{3'}$-H, C_{16}-H), 3.23 (m, 1H, one of C_2-H), 2.99-2.95 (m, 2H, C_3-H, $C_{4'}$-H), 2.80 (m, 1H, C_{12}-H), 2.67 (m, 1H, one of C_2-H), 2.43 (m, 2H, C_{10}-H), 2.16 (m, 1H, C_7-H), 1.94 (m, 1H, one of C_8-H), 1.67 (m, 2H, one of C_5-H, one of C_6-H), 1.58 (m, 1H, $C_{2''}$-H), 1.47-1.42 (m, 7H, one of C_8-H, C_{18}-H, one of C_{19}-H, one of C_{20}-H, C_{22}-H), 1.34-1.32 (m, 5H, one of C_{19}-H, one of C_{20}-H, $C_{6'}$-H), 1.20-1.10 (m, 8H, one of C_5-H, one of C_6-H, C_{16}-CH_3, $C_{3'}$-OC-CH_3), 0.97-0.90 (m, 5H, $C_{3''}$-H, $C_{4''}$-H, C_{11}-H), 0.77 (t, 3H, C_{23}-H); ^{13}C NMR (101 MHz, $CDCl_3$) δ: 201.48, 174.28, 172.41, 149.15, 144.99, 95.71, 82.08, 79.48, 78.36, 76.27, 75.70, 74.66, 67.80, 65.39, 60.69, 58.90, 50.03, 49.04, 47.75, 46.34, 45.60, 43.20, 40.84, 39.44, 38.65, 37.94, 32.79, 32.34, 29.94, 28.04, 27.35, 26.83, 21.32, 17.57, 15.50, 12.57, 8.15, 8.01; MS (MALDI) cal for $C_{38}H_{60}O_9$ $[M+Na]^+$ 683.412954, found $[M+ Na]^+$ 683.413206.

Preparation of 7c

Compound **5** (0.34 g, 0.56 mmol) and 3,5-dimethoxybenzy-2,2,2-trichloroacetimi-date (0.19 g, 0.61 mmol) were dissolved in 15 mL dry CH_2Cl_2 with some molecular sieve under Ar. Then $BF_3·(C_2H_5)_2O$ (0.11 mL, 1.00 mmol) was added at room temperature. The mixture was stirred for 18 h, then diluted with CH_2Cl_2 (15 mL) and washed with saturated sodium bicarbonate solution (3 × 10 mL). The combined

organic layers were dried with Na_2SO_4 and evaporated under reduced pressure. The residue was purified by column chromatography on silica gel (200–300 mesh) to afford pure **7c** (0.26 g, yield 61%). TLC (ethyl acetate : petroleum ether = 1:2, v:v). ^{1}H NMR (400 MHz, $CDCl_3$) δ: 6.79 (s, 1H, C_{13}-H), 6.45-6.31 (m, 3H, $C_{3''}$-H, $C_{5''}$-H, $C_{7''}$-H), 5.04 (s, 1H, $C_{1'}$-H), 4.74 (s, 2H, $C_{1''}$-H), 4.57 (m, 1H, C_{21}-H), 4.14 (m, 1H, C_9-H), 3.72 (s, 6H, $C_{4''}$-OCH_3, $C_{6''}$-OCH_3), 3.67-3.60 (m, 2H, $C_{2'}$-H, C_{17}-H), 3.55-3.43 (m, 11H, $C_{3'}$-OCH_2-, $C_{4'}$-OCH_3, $C_{5'}$-H, C_4-H, $C_{2'}$-OCH_3, $C_{3'}$-H), 3.36 (m, 1H, C_{16}-H), 3.11-2.99 (m, 2H , one of C_2-H, C_3-H), 2.89 (m, 1H, $C_{4'}$-H), 2.74 (m, 1H, C_{12}-H), 2.38 (m, 1H, one of C_2-H), 2.28 (m, 1H, C_7-H), 2.18 (m, 2H, C_{10}-H), 1.74-1.49 (m, 12H, one of C_8-H, C_5-H, C_6-H, one of C_8-H, C_{18}-H, one of C_{19}-H, one of C_{20}-H, C_{22}-H), 1.20-1.11 (m, 11H, one of C_{19}-H, one of C_{20}-H, $C_{6'}$-H, C_{16}-CH_3, $C_{3'}$-OC-CH_3), 1.01 (m, 1H, C_{11}-H), 0.76 (t, 3H, C_{23}-H); ^{13}C NMR (101 MHz, $CDCl_3$) δ: 201.72, 171.47, 159.98, 159.85, 148.51, 144.86, 143.89, 104.77, 103.56, 99.52, 94.81, 81.22, 78.69, 77.55, 74.73, 74.58, 68.41, 66.98, 64.58, 59.98, 58.20, 54.35, 54.32, 49.18, 47.10, 45.48, 44.73, 42.53, 39.94, 38.56, 37.77, 37.06, 33.67, 31.84, 30.89, 29.04, 27.19, 25.97, 20.20, 16.84, 15.33, 14.74, 8.36; MS (MALDI) cal for $C_{43}H_{64}O_{11}$ $[M+Na]^+$ 779.434084, found $[M+ Na]^+$ 779.434141.

Preparation of 7d

Compound **5** (0.33 g, 0.54 mmol) and 4-chlorobenzyl-2,2,2-trichloroacetimidate (0.18 g, 0.63 mmol) were dissolved in 15 mL dry CH_2Cl_2 with some molecular sieve under Ar. Then $BF_3{\cdot}(C_2H_5)_2O$ (0.14 mL, 1.03 mmol) was added at room temperature. The mixture was stirred for 26 h, then diluted with CH_2Cl_2 (15 mL) and washed with saturated sodium bicarbonate solution (3 × 10 mL). The combined organic layers were dried with Na_2SO_4 and evaporated under reduced pressure. The residue was purified by column chromatography on silica gel (200–300 mesh) to afford pure **7d** (0.31g, yield 79%). TLC (ethyl acetate : petroleum ether = 1:2, v:v). ^{1}H NMR (400 MHz, $CDCl_3$) δ: 7.24-7.13 (m, 4H, $C_{3''}$-H, $C_{4''}$-H, $C_{6''}$-H, $C_{7''}$-H), 6.79 (s, 1H, C_{13}-H), 4.98 (s, 1H, $C_{1'}$-H), 4.73 (m, 1H, C_{21}-H), 4.60 (s, 2H, $C_{1''}$-H), 4.37 (m, 1H, C_9-H), 4.15 (m, 1H, $C_{2'}$-H), 4.01 (m, 1H, C_{17}-H), 3.55-3.42 (m, 11H, $C_{3'}$-OCH_2-, $C_{4'}$-OCH_3, $C_{5'}$-H, C_4-H, $C_{2'}$-OCH_3, $C_{3'}$-H), 3.28 (m, 1H, C_{16}-H), 3.13-2.79 (m, 4H, one of C_2-H, C_3-H, $C_{4'}$-H,

C_{12}-H), 2.42 (m, 1H, one of C_2-H), 2.28 (m, 1H, C_7-H), 2.16 (m, 2H, C_{10}-H), 1.85-1.35 (m, 12H, one of C_8-H, C_5-H, C_6-H, one of C_8-H, C_{18}-H, one of C_{19}-H, one of C_{20}-H, C_{22}-H), 1.27-1.13 (m, 11H, one of C_{19}-H, one of C_{20}-H, $C_{6'}$-H, C_{16}-CH_3, $C_{3'}$-OC-CH_3), 1.02 (m, 1H, C_{11}-H), 0.83 (t, 3H, C_{23}-H); ^{13}C NMR (101 MHz, $CDCl_3$) δ: 202.06, 162.78, 148.47, 145.03, 128.71, 128.26, 128.11, 127.82, 127.61, 127.25, 90.89, 81.01, 78.63, 77.57, 77.19, 70.82, 67.26, 66.96, 64.60, 63.42, 59.97, 58.15, 49.13, 45.32, 43.18, 42.58, 41.16, 39.78, 37.81, 37.07, 35.72, 32.43, 29.57, 28.44, 26.70, 23.96, 23.69, 18.13, 16.80, 14.71, 9.07; MS (MALDI) cal for $C_{41}H_{59}ClO_9$ $[M+Na]^+$ 753.373982, found $[M+ Na]^+$ 753.373992.

Preparation of 7e

Compound **5** (0.35 g, 0.58 mmol) and 2,6-difluorobenzyl-2,2,2-trichloroacetimidate (0.19 g, 0.66 mmol) were dissolved in 15 mL dry CH_2Cl_2 with some molecular sieve under Ar. Then $BF_3{\cdot}(C_2H_5)_2O$ (0.12 mL, 1.01 mmol) was added at room temperature. The mixture was stirred for 23 h, then diluted with CH_2Cl_2 (15 mL) and washed with saturated sodium bicarbonate solution (3 × 10 mL). The combined organic layers were dried with Na_2SO_4 and evaporated under reduced pressure. The residue was purified by column chromatography on silica gel (200–300 mesh) to afford pure **7e** (0.34 g, yield 80%). TLC (ethyl acetate : petroleum ether = 1:2, v:v). ^1H NMR (400 MHz, $CDCl_3$) *δ*: 6.97-6.68 (m, 4H, $C_{4''}$-H, $C_{5''}$-H, $C_{6''}$-H, C_{13}-H), 4.72 (s, 1H, $C_{1'}$-H), 4.57 (s, 2H, $C_{1''}$-H), 4.48 (m, 1H, C_{21}-H), 4.22 (m, 1H, C_9-H), 4.02 (m, 1H, $C_{2'}$-H), 3.63 (m, 1H, C_{17}-H), 3.54-3.41 (m, 11H, $C_{3'}$-OCH2-, $C_{4'}$-OCH3, $C_{5'}$-H, C_4-H, $C_{2'}$-OCH_3, $C_{3'}$-H), 3.18-3.02 (m, 3H, C_{16}-H, one of C_2-H, C_3-H), 2.86 (m, 1H, $C_{4'}$-H), 2.72 (m, 1H, C_{12}-H), 2.43 (m, 1H, one of C_2-H), 2.32 (m, 2H, C_{10}-H), 2.17 (m, 1H, C_7-H), 1.95 (m, 1H, one of C_8-H), 1.72-1.41 (m, 11H, C_5-H, C_6-H, one of C_8-H, C_{18}-H, one of C_{19}-H, one of C_{20}-H, C_{22}-H), 1.33-1.17 (m, 11H, one of C_{19}-H, one of C_{20}-H, $C_{6'}$-H, C_{16}-CH_3, $C_{3'}$-OC-CH_3), 0.96 (m, 1H, C_{11}-H), 0.74 (t, 3H, C_{23}-H); ^{13}C NMR (101 MHz, $CDCl_3$) δ: 203.07, 172.37, 163.55, 163.12, 149.00, 144.76, 130.08, 113.92, 111.31, 111.06, 95.65, 82.11, 80.77, 79.44, 78.47, 76.21, 75.61, 67.84, 65.45, 60.75, 60.23, 58.99, 50.22, 46.70, 46.17, 43.56, 40.66, 39.44, 38.67, 38.06, 33.35, 31.66, 30.40, 27.95, 26.84, 24.62, 20.51, 17.72, 16.88, 15.63, 9.25; MS (MALDI) cal for

$C_{41}H_{58}F_2O_9$ $[M+Na]^+$ 755.394111, found $[M+ Na]^+$ 755.393714.

Preparation of 7f

Compound **5** (0.34 g, 0.56 mmol) and 3-trifluoromethylbenzyl-2,2,2-trichloroacetimidate (0.21 g, 0.66 mmol) were dissolved in 15 mL dry CH_2Cl_2 with some molecular sieve under Ar. Then $BF_3 \cdot (C_2H_5)_2O$ (0.15 mL, 1.03 mmol) was added at room temperature. The mixture was stirred for 18 h, then diluted with CH_2Cl_2 (15 mL) and washed with saturated sodium bicarbonate solution (3 × 10 mL). The combined organic layers were dried with Na_2SO_4 and evaporated under reduced pressure. The residue was purified by column chromatography on silica gel (200–300 mesh) to afford pure **7f** (0.33 g, yield 77%). TLC (ethyl acetate : petroleum ether = 1:2, v:v). ^1H NMR (400 MHz, $CDCl_3$) δ: 7.56-7.40 (m, 4H, $C_{3''}$-H, $C_{5''}$-H, $C_{6''}$-H, $C_{7''}$-H), 6.81 (s, 1H, C_{13}-H), 6.64-6.48 (m, 3H, C_{16}-H, one of C_2-H, C_3-H), 4.83-4.57 (m, 6H, $C_{1'}$-H, C_{21}-H, $C_{1''}$-H, C_9-H, $C_{2'}$-H), 4.14 (m, 1H, C_{17}-H), 3.64-3.27 (m, 11H, $C_{3'}$-OCH_2-, $C_{4'}$-OCH_3, $C_{5'}$-H, C_4-H, $C_{2'}$-OCH_3, $C_{3'}$-H), 2.95 (m, 1H, $C_{4'}$-H), 2.79 (m, 1H, C_{12}-H), 2.52-2.15 (m, 4H, one of C_2-H, C_7-H, C_{10}-H), 1.96 (m, 1H, one of C_8-H), 1.86-1.30 (m, 11H, C_5-H, C_6-H, one of C_8-H, C_{18}-H, one of C_{19}-H, one of C_{20}-H, C_{22}-H), 1.27-1.11 (m, 11H, one of C_{19}-H, one of C_{20}-H, $C_{6'}$-H, C_{16}-CH_3, $C_{3'}$-OC-CH_3), 0.91 (m, 1H, C_{11}-H), 0.75 (t, 3H, C_{23}-H); ^{13}C NMR (101 MHz, $CDCl_3$) δ: 202.38, 171.60, 162.94, 148.46, 143.79, 140.83, 129.97, 129.03, 127.91, 123.26, 122.48, 94.72, 90.90, 81.21, 80.09, 78.58, 77.59, 74.73, 70.87, 70.19, 66.95, 64.63, 63.35, 59.91, 58.13, 49.29, 45.98, 45.33, 42.58, 39.78, 38.58, 37.75, 37.11, 32.46, 29.52, 27.01, 25.90, 23.66, 18.11, 16.79, 14.69, 8.32; MS (MALDI) cal for $C_{42}H_{59}F_3O_9$ $[M+Na]^+$ 787.400339, found $[M+ Na]^+$ 787.400000.

Preparation of 7g

Compound **5** (0.35 g, 0.58 mmol) and 2-fluorobenzyl-2,2,2-trichloroacetimidate (0.18 g, 0.67 mmol) were dissolved in 15 mL dry CH_2Cl_2 with some molecular sieve under Ar. Then $BF_3 \cdot (C_2H_5)_2O$ (0.12 mL, 1.01 mmol) was added at room temperature. The mixture was stirred for 22 h, then diluted with CH_2Cl_2 (15 mL) and washed with saturated sodium bicarbonate solution (3 × 10 mL). The combined organic layers were dried with Na_2SO_4 and evaporated under reduced pressure. The residue was purified

by column chromatography on silica gel (200–300 mesh) to afford pure **7g** (0.29 g, yield 70%). TLC (ethyl acetate : petroleum ether = 1:2, v:v). ^{1}H NMR (700 MHz, $CDCl_3$) δ: 7.43 (m, 1H, $C_{5''}$-H), 7.26 (m, 1H, $C_{4''}$-H), 7.14 (m, 1H, $C_{7''}$-H), 7.04 (m, 1H, $C_{6''}$-H), 6.86 (s, 1H, C_{13}-H), 4.80 (s, 1H, $C_{1'}$-H), 4.64 (m, 1H, C_{21}-H), 4.21 (m, 1H, C_9-H), 3.72 (m, 1H, $C_{2'}$-H), 3.62 (m, 1H, C_{17}-H), 3.57-3.55 (m, 5H, $C_{3'}$-OCH_2-, $C_{4'}$-OCH_3), 3.54 (m, 1H, $C_{5'}$-H), 3.51 (m, 1H, C_4-H), 3.49 (s, 3H, $C_{2'}$-OCH_3), 3.44 (m, 1H, $C_{3'}$-H), 3.15-3.09 (m, 3H, C_{16}-H, one of C_2-H, C_3-H), 2.94 (m, 1H, $C_{4'}$-H), 2.81 (m, 1H, C_{12}-H), 2.58 (m, 1H, C_7-H), 2.36 (dd, 1H, J=14Hz, one of C_2-H), 2.25 (m, 2H, C_{10}-H), 1.93 (m, 1H, one of C_8-H), 1.68-1.47 (m, 11H, C_5-H, C_6-H, one of C_8-H, C_{18}-H, one of C_{19}-H, one of C_{20}-H, C_{22}-H), 1.29-1.25 (m, 11H, one of C_{19}-H, one of C_{20}-H, $C_{6'}$-H, C_{16}-CH_3, $C_{3'}$-OC-CH_3), 0.91 (m, 1H, C_{11}-H), 0.82 (t, 3H, C_{23}-H); ^{13}C NMR (176 MHz, $CDCl_3$) δ: 203.40, 172.61, 163.71, 149.26, 144.85, 129.50, 129.27, 124.20, 124.06, 115.34, 95.66, 82.18, 79.61, 78.54, 78.16, 75.64, 68.11, 67.94, 65.61, 60.98, 59.17, 50.26, 47.48, 46.95, 46.33, 43.57, 40.77, 39.53, 38.75, 38.11, 33.45, 31.82, 30.51, 28.05, 26.93, 24.68, 23.49, 17.83, 17.27, 15.75, 9.39; MS (MALDI) cal for $C_{41}H_{59}FO_9$ $[M+Na]^+$ 737.403532, found $[M+ Na]^+$ 737.403716.

Preparation of 7h

Compound **5** (0.33 g, 0.54 mmol) and 1,4-di(2,2,2-trichloroacetimidatemethyl)benzene (0.26 g, 0.61 mmol) were dissolved in 15 mL dry CH_2Cl_2 with some molecular sieve under Ar. Then $BF_3{\cdot}(C_2H_5)_2O$ (0.13 mL, 1.02 mmol) was added at room temperature. The mixture was stirred for 20 h, then diluted with CH_2Cl_2 (15 mL) and washed with saturated sodium bicarbonate solution (3 × 10 mL). The combined organic layers were dried with Na_2SO_4 and evaporated under reduced pressure. The residue was purified by column chromatography on silica gel (200–300 mesh) to afford pure **7h** (0.32 g, yield 68%). TLC (ethyl acetate : petroleum ether = 1:2, v:v). ^{1}H NMR (400 MHz, $CDCl_3$) δ: 9.70(s, 1H, N-H), 7.28(m, 4H, $C_{3''}$-H, $C_{4''}$-H, $C_{6''}$-H, $C_{7''}$-H,), 6.83(s, 1H, C_{13}-H), 4.83(s, 1H, $C_{1'}$-H), 4.76(m, 1H, C_{21}-H), 4.59(s, 2H, $C_{1''}$-H), 4.58(m, 2H, $C_{8''}$-H), 4.17(m, 1H, C_9-H), 3.69(m, 1H, $C_{2'}$-H), 3.62(m, 1H, C_{17}-H), 3.62-3.55(m, 5H, $C_{3'}$-OCH_2-, $C_{4'}$-OCH_3), 3.54(m, 1H, $C_{5'}$-H), 3.53-3.52(m, 4H, C_4-H, $C_{2'}$-OCH_3), 3.50-3.49(m, 3H, C_{16}-H, one of C_2-H, C_3-H), 3.44(m, 1H,

$C_{3'}$-H), 3.03(m, 1H, $C_{4'}$-H), 2.89(m, 1H, C_{12}-H), 2.77(m, 1H, C_7-H), 2.55(m, 1H, one of C_8-H), 2.31(dd, 1H, *J*=14Hz, one of C_2-H), 2.21(m, 2H, C_{10}-H), 1.77-1.49(m, 11H, C_5-H, C_6-H, one of C_8-H, C_{18}-H, one of C_{19}-H, one of C_{20}-H, C_{22}-H), 1.23-1.21(m, 11H, one of C_{19}-H, one of C_{20}-H, $C_{6'}$-H, C_{16}-CH_3, $C_{3'}$-OC-CH_3), 1.01(m, 1H, C_{11}-H), 0.77(t, 3H, C_{23}-H); ^{13}C NMR (176 MHz, CDCl3) δ: 202.70, 172.47, 171.10, 149.17, 144.73, 136.07, 135.62, 128.44, 128.00, 127.79, 127.35, 99.38, 95.76, 82.13, 79.63, 78.45, 78.33, 76.16, 75.69, 67.91, 65.61, 60.97, 60.34, 59.17, 58.96, 50.22, 47.01, 46.31, 43.49, 40.74, 39.55, 38.75, 38.11, 34.16, 33.36, 30.57, 27.99, 26.88, 24.64, 20.99, 17.80, 15.69, 14.16, 9.37; MS (MALDI) cal for $C_{44}H_{62}Cl_3NO_{10}$ $[M+Na]^+$ 892.333151, found $[M+ Na]^+$ 892.333331.

Preparation of 7i

Compound **5** (0.38 g, 0.63 mmol) and 3-methoxybenzyl-2,2,2-trichloroacetimidate (0.21 g, 0.75 mmol) were dissolved in 15 mL dry CH_2Cl_2 with some molecular sieve under Ar. Then $BF_3{\cdot}(C_2H_5)_2O$ (0.12 mL, 1.01 mmol) was added at room temperature. The mixture was stirred for 24 h, then diluted with CH_2Cl_2 (15 mL) and washed with saturated sodium bicarbonate solution (3 × 10 mL). The combined organic layers were dried with Na_2SO_4 and evaporated under reduced pressure. The residue was purified by column chromatography on silica gel (200–300 mesh) to afford pure **7i** (0.27 g, yield 59%). TLC (ethyl acetate : petroleum ether = 1:2, v:v). ^{1}H NMR (400 MHz, $CDCl_3$) δ: 7.65 (m, 1H, $C_{6''}$-H), 7.45 (m, 1H, $C_{7''}$-H), 7.20-7.16 (m, 2H, $C_{3''}$-H, $C_{5''}$-H), 6.84 (s, 1H, C_{13}-H), 5.21 (s, 2H, $C_{1''}$-H), 4.73 (s, 1H, $C_{1'}$-H), 4.59 (m, 1H, C_{21}-H), 4.14 (m, 1H, C_9-H), 3.73 (s, 3H, $C_{4''}$-OCH_3), 3.71 (m, 1H, $C_{2'}$-H), 3.65 (m, 1H, C_{17}-H), 3.63-3.57 (m, 5H, $C_{3'}$-OCH_2-, $C_{4'}$-OCH_3), 3.55 (m, 1H, $C_{5'}$-H), 3.52 (m, 1H, C_4-H), 3.49 (s, 3H, $C_{2'}$-OCH_3), 3.46 (m, 1H, $C_{3'}$-H), 3.42-3.40 (m, 3H, C_{16}-H, one of C_2-H, C_3-H), 3.03 (m, 1H, $C_{4'}$-H), 2.86 (m, 1H, C_{12}-H), 2.51 (m, 1H, C_7-H), 2.29 (m, 1H, one of C_2-H), 2.18 (m, 2H, C_{10}-H), 1.96 (m, 1H, one of C_8-H), 1.78-1.42 (m, 11H, C_5-H, C_6-H, one of C_8-H, C_{18}-H, one of C_{19}-H, one of C_{20}-H, C_{22}-H), 1.34-1.23 (m, 11H, one of C_{19}-H, one of C_{20}-H, $C_{6'}$-H, C_{16}-CH_3, $C_{3'}$-OC-CH_3), 1.04 (m, 1H, C_{11}-H), 0.90 (t, 3H, C_{23}-H); 13C NMR (101 MHz, CDCl3) δ 202.42, 171.57, 162.70, 148.20, 143.84, 139.12, 129.94, 119.07, 112.28, 112.12, 94.66, 81.17, 79.62, 78.60, 75.23,

74.62, 70.80, 66.93, 64.59, 59.95, 58.15, 54.18, 52.43, 49.25, 46.02, 45.32, 42.56, 39.76, 38.53, 37.74, 37.11, 32.43, 30.90, 29.54, 28.67, 27.03, 23.67, 21.67, 18.14, 16.83, 14.73, 8.37; MS (MALDI) cal for $C_{42}H_{62}O_{10}$ $[M+Na]^+$ 749.423519, found $[M+ Na]^+$ 749.423777.

Preparation of 7j

Compound **5** (0.36 g, 0.59 mmol) and D-cellobioseoctaacetate-2,2,2-trichloroacetimidate (0.53 g, 0.68 mmol) were dissolved in 15 mL dry CH_2Cl_2 with some molecular sieve under Ar. Then $BF_3{\cdot}(C_2H_5)_2O$ (0.13 mL, 1.02 mmol) was added at room temperature. The mixture was stirred for 12 h, then diluted with CH_2Cl_2 (15 mL) and washed with saturated sodium bicarbonate solution (3 × 10 mL). The combined organic layers were dried with Na_2SO_4 and evaporated under reduced pressure. The residue was purified by column chromatography on silica gel (200–300 mesh) to afford pure **7j** (0.47 g, yield 65%). TLC (ethyl acetate : petroleum ether = 1:2, v:v). ^1H NMR (400 MHz, $CDCl_3$) δ: 6.89 (s, 1H, C_{13}-H), 5.17 (m, 2H, $C_{1''}$-H, $C_{6''}$-H), 5.07 (m, 1H, $C_{8''}$-H), 4.92 (m, 2H, $C_{2''}$-H, $C_{7''}$-H), 4.80 (s, 1H, $C_{1'}$-H), 4.61-4.57 (m, 2H, C_{21}-H, $C_{3''}$-H), 4.39 (m, 1H, $C_{9''}$-H), 4.21 (m, 1H, C_9-H), 4.14-4.03 (m, 6H, $C_{5''}$-H, $C_{10''}$-H, $C_{5''}$-CH_2-, $C_{10''}$-CH_2-), 3.79 (m, 1H, $C_{4''}$-H), 3.71 (m, 1H, $C_{2'}$-H), 3.60 (m, 1H, C_{17}-H), 3.56-3.49 (m, 10H, $C_{3'}$-OCH_2-, $C_{4'}$-OCH_3, $C_{5'}$-H, C_4-H, $C_{2'}$-OCH_3), 3.44 (m, 1H, $C_{3'}$-H), 3.12-3.07 (m, 3H, C_{16}-H, one of C_2-H, C_3-H), 2.93 (m, 1H, $C_{4'}$-H), 2.81 (m, 1H, C_{12}-H), 2.35 (m, 1H, one of C_2-H), 2.32 (m, 1H, C_7-H), 2.24 (m, 2H, C_{10}-H), 2.04-1.98 (m, 21H, -OAc×5, -C-OAc×2), 1.92 (m, 1H, one of C_8-H), 1.61-1.47 (m, 11H, C_5-H, C_6-H, one of C_8-H, C_{18}-H, one of C_{19}-H, one of C_{20}-H, C_{22}-H), 1.24-1.14 (m, 11H, one of C_{19}-H, one of C_{20}-H, $C_{6'}$-H, C_{16}-CH_3, $C_{3'}$-OC-CH_3), 1.03 (m, 1H, C_{11}-H), 0.81 (t, 3H, C_{23}-H); ^{13}C NMR (101 MHz, $CDCl_3$) δ 202.31, 172.20, 170.80, 170.25, 169.98, 169.64, 169.30, 169.10, 168.84, 149.55, 144.52, 101.42, 100.56, 95.69, 82.00, 79.53, 78.32, 76.42, 76.16, 75.52, 72.76, 72.57, 72.45, 71.91, 71.71, 71.45, 67.78, 65.34, 61.82, 61.43, 60.75, 60.13, 58.99, 50.00, 46.93, 46.29, 43.18, 40.71, 39.41, 38.63, 37.94, 33.85, 33.02, 30.16, 28.09, 26.82, 24.42, 21.95, 20.92, 20.81, 20.61, 20.53, 20.44, 20.33, 20.30, 17.67, 16.19, 15.59, 14.04, 9.26; MS (MALDI) cal for $C_{60}H_{88}O_{26}$ $[M+Na]^+$ 1247.545604, found $[M+ Na]^+$ 1247.545361.

Preparation of 7k

Compound **5** (0.34 g, 0.56 mmol) and β-D-ribofuranose-1,2,3,5-tetraacetate-2,2,2-trichloroacetimidate (0.26 g, 0.62 mmol) were dissolved in 15 mL dry CH_2Cl_2 with some molecular sieve under Ar. Then $BF_3{\cdot}(C_2H_5)_2O$ (0.12 mL, 1.01 mmol) was added at room temperature. The mixture was stirred for 16 h, then diluted with CH_2Cl_2 (15 mL) and washed with saturated sodium bicarbonate solution (3 × 10 mL). The combined organic layers were dried with Na_2SO_4 and evaporated under reduced pressure. The residue was purified by column chromatography on silica gel (200–300 mesh) to afford pure **7k** (0.32 g, yield 67%). TLC (ethyl acetate : petroleum ether = 1:3, v:v). ^1H NMR (400 MHz, $CDCl_3$) δ: 6.80 (s, 1H, C_{13}-H), 6.71 (m, 1H, $C_{1''}$-H), 5.40-4.80 (m, 3H, $C_{2''}$-H, $C_{3''}$-H, $C_{4''}$-H), 4.74 (s, 1H, $C_{1'}$-H), 4.57 (m, 1H, C_{21}-H), 4.33-4.02 (m, 4H, $C_{1''}$-H, C_9-H, $C_{4''}$-CH_2-), 3.69-3.54 (m, 7H, $C_{2'}$-H, C_{17}-H, $C_{3'}$-OCH2-, $C_{4'}$-OCH_3), 3.50-3.42 (m, 6H, $C_{5'}$-H, C_4-H, $C_{2'}$-OCH_3, $C_{3'}$-H), 3.08-2.99 (m, 3H, C_{16}-H, one of C_2-H, C_3-H), 2.86 (m, 1H, $C_{4'}$-H), 2.74 (m, 1H, C_{12}-H), 2.52 (m, 1H, C_7-H), 2.28 (dd, J_1=6.6Hz, J_2=1.4Hz, 1H, one of C_2-H), 2.16 (m, 2H, C_{10}-H), 2.04-1.97 (m, 6H, $C_{2''}$-OAc, $C_{3''}$-OAc), 1.74 (m, 3H, $C_{4''}$-OAc), 1.66 (m, 1H, one of C_8-H), 1.56-1.29 (m, 11H, C_5-H, C_6-H, one of C_8-H, C_{18}-H, one of C_{19}-H, one of C_{20}-H, C_{22}-H), 1.18-1.06 (m, 11H, one of C_{19}-H, one of C_{20}-H, $C_{6'}$-H, C_{16}-CH_3, $C_{3'}$-OC-CH_3), 0.96 (m, 1H, C_{11}-H), 0.75 (t, *J=7.6Hz*, 3H, C_{23}-H); ^{13}C NMR (101 MHz, $CDCl_3$) δ 201.73, 171.51, 170.14, 169.53, 168.75, 148.16, 143.78, 98.40, 94.79, 81.29, 81.18, 80.99, 78.66, 77.50, 75.19, 74.72, 67.49, 66.95, 64.66, 64.58, 59.98, 59.38, 58.20, 49.26, 48.16, 46.05, 45.34, 42.53, 39.78, 38.59, 37.79, 33.20, 32.40, 29.62, 27.03, 25.92, 23.67, 20.03, 19.85, 19.70, 19.59, 16.83, 14.75, 14.71, 8.39; MS (MALDI) cal for $C_{45}H_{68}O_{16}$ $[M+Na]^+$ 887.439947, found $[M+ Na]^+$ 887.440211.

Preparation of 7l

Compound **5** (0.33 g, 0.54 mmol) and β-D-glucosepentaacetate-2,2,2-trichloro-acetimidate (0.28 g, 0.45 mmol) were dissolved in 15 mL dry CH_2Cl_2 with some molecular sieve under Ar. Then $BF_3{\cdot}(C_2H_5)_2O$ (0.14 mL, 1.03 mmol) was added at room temperature. The mixture was stirred for 18 h, then diluted with CH_2Cl_2 (15 mL) and washed with saturated sodium bicarbonate solution (3 × 10 mL). The combined

organic layers were dried with Na_2SO_4 and evaporated under reduced pressure. The residue was purified by column chromatography on silica gel (200–300 mesh) to afford pure **7l** (0.32 g, yield 63%). TLC (ethyl acetate : petroleum ether = 1:2, v:v). ^1H NMR (400 MHz, $CDCl_3$) δ: 6.82 (s, 1H, C_{13}-H), 5.26 (m, 4H, $C_{1''}$-H, $C_{2''}$-H, $C_{3''}$-H, $C_{4''}$-H,), 4.92 (m, 1H, $C_{5''}$-H), 4.73 (s, 1H, $C_{1'}$-H), 4.59 (m, 1H, C_{21}-H), 4.15 (m, 1H, C_9-H), 3.65 (m, 1H, $C_{2'}$-H), 3.55 (m, 1H, C_{17}-H), 3.53-3.36 (m, 11H, $C_{3'}$-OCH_2-, $C_{4'}$-OCH_3, $C_{5'}$-H, C_4-H, $C_{2'}$-OCH_3, $C_{3'}$-H), 3.29 (m, 1H, one of C_2-H), 3.08-3.00 (m, 2H, C_{16}-H, C_3-H), 2.88 (m, 1H, $C_{4'}$-H), 2.75 (m, 1H, C_{12}-H), 2.50 (m, 1H, C_7-H), 2.36-2.12 (m, 3H, one of C_2-H, C_{10}-H), 2.03-1.27 (m, 23H, one of C_8-H, one of C_{19}-H, one of C_{20}-H, $C_{6'}$-H, C_{16}-CH_3, $C_{3'}$-OC-CH_3, C_5-H, C_6-H, one of C_8-H, C_{18}-H, one of C_{19}-H, one of C_{20}-H, C_{22}-H), 1.04 (d, 2H, $J=6.8Hz$, $C_{1''}$-CH_2-), 0.95 (m, 1H, C_{11}-H), 0.74 (t, 3H, $J=7.6Hz$, C_{23}-H); ^{13}C NMR (101 MHz, $CDCl_3$) δ 201.54, 172.40, 170.99, 170.48, 163.49, 149.30, 145.01, 95.78, 95.71, 82.10, 79.57, 78.40, 78.36, 76.33, 75.75, 75.58, 74.86, 74.68, 67.83, 65.44, 60.85, 60.26, 59.05, 53.46, 50.04, 46.42, 45.57, 43.19, 40.90, 39.47, 38.70, 37.96, 32.86, 32.45, 29.89, 28.15, 26.88, 24.42, 21.46, 20.92, 20.91, 20.59, 20.40, 17.73, 15.95, 15.65, 14.10, 9.29; MS (MALDI) cal for $C_{48}H_{72}O_{18}$ $[M+Na]^+$ 959.461086, found $[M+ Na]^+$ 959.461240.

Preparation of 8b

Compound **5** (0.36 g, 0.59 mmol), propionyl chloride (0.05 g, 0.64 mmol) and 4-dimethylaminopyridine (DMAP, 0.17 g, 1.39 mmol) were added to 15 mL CH_2Cl_2 under Ar, then the mixture was heated to reflux. After about 8 h, the mixture was diluted with CH_2Cl_2 (15 mL) and washed with saturated sodium bicarbonate solution (3 × 10 mL). The combined organic layers were dried with Na_2SO_4 and evaporated under reduced pressure. The residue was purified by column chromatography on silica gel (200–300 mesh) to afford pure **8b** (0.34 g, yield 87%). TLC (ethyl acetate : petroleum ether = 1:3, v:v). ^1H NMR (400 MHz, $CDCl_3$) δ: 6.75 (s, 1H, C_{13}-H), 4.86 (s, 1H, $C_{1'}$-H), 4.67 (m, 1H, C_{21}-H), 4.51 (m, 1H, C_9-H), 4.07 (m, 1H, $C_{2'}$-H), 3.58 (m, 1H, C_{17}-H), 3.50-3.31 (m, 11H, $C_{3'}$-OCH_2-, $C_{4'}$-OCH_3, $C_{5'}$-H, C_4-H, $C_{2'}$-OCH_3, $C_{3'}$-H), 3.24 (m, 1H, C_{16}-H), 3.00-2.95 (m, 2H, one of C_2-H, C_3-H), 2.81 (m, 1H, $C_{4'}$-H), 2.68 (m, 1H, C_{12}-H), 2.44 (m, 1H, one of C_2-H), 2.25-2.14 (m, 4H, C_7-H, C_{10}-H, one of

C_8-H), 1.68-1.32 (m, 11H, C_5-H, C_6-H, one of C_8-H, C_{18}-H, one of C_{19}-H, one of C_{20}-H, C_{22}-H), 1.13-0.96 (m, 17H, one of C_{19}-H, one of C_{20}-H, $C_{6'}$-H, C_{16}-CH_3, $C_{3'}$-OC-CH_3, C_{11}-H, $C_{2''}$-H, $C_{3''}$-H), 0.67 (t, *J*=7.4Hz, 3H, C_{23}-H); ^{13}C NMR (101 MHz, $CDCl_3$) δ 201.41, 173.70, 172.34, 149.05, 145.00, 95.73, 82.07, 79.55, 78.37, 75.59, 74.53, 67.80, 65.37, 60.75, 58.97, 50.02, 47.74, 46.35, 45.54, 43.20, 40.83, 39.44, 38.66, 37.95, 32.82, 32.47, 29.93, 28.07, 27.56, 27.03, 24.40, 21.37, 17.65, 15.94, 15.57, 9.22, 8.81; MS (MALDI) cal for $C_{37}H_{58}O_{10}$ $[M+Na]^+$ 685.392219, found $[M+ Na]^+$ 685.392352.

Preparation of 8c

Compound **5** (0.36 g, 0.59 mmol), 2-thiophenoyl chloride (0.11 g, 0.75 mmol) and DMAP (0.17 g, 1.39 mmol) were added to 15 mL CH_2Cl_2 under Ar, then the mixture was heated to reflux. After about 11 h, the mixture was diluted with CH_2Cl_2 (15 mL) and washed with saturated sodium bicarbonate solution (3 × 10 mL). The combined organic layers were dried with Na_2SO_4 and evaporated under reduced pressure. The residue was purified by column chromatography on silica gel (200–300 mesh) to afford pure **8c** (0.31 g, yield 74%). TLC (ethyl acetate : petroleum ether = 1:4, v:v). ^{1}H NMR (400 MHz, $CDCl_3$) δ: 7.74 (d, 1H, C3”-H), 7.50 (d, 1H, C5”-H), 7.04 (m, 1H, C4”-H), 6.86 (s, 1H, C_{13}-H), 4.74 (s, 1H, $C_{1'}$-H), 4.61 (m, 1H, C_{21}-H), 4.14 (m, 1H, C_9-H), 3.65 (m, 1H, $C_{2'}$-H), 3.55 (m, 1H, C_{17}-H), 3.50-3.48 (m, 6H, $C_{3'}$-OCH_2-, $C_{4'}$-OCH_3, $C_{5'}$-H), 3.44-3.38 (m, 8H, C_4-H, $C_{2'}$-OCH_3, $C_{3'}$-H, C_{16}-H, one of C_2-H, C_3-H), 3.08 (m, 1H, $C_{4'}$-H), 2.77 (m, 1H, C_{12}-H), 2.51 (m, 1H, C_7-H), 2.31 (s, 1H, one of C_2-H), 2.21 (m, 2H, C_{10}-H), 1.89 (m, 1H, one of C_8-H), 1.65-1.39 (m, 11H, C_5-H, C_6-H, one of C_8-H, C_{18}-H, one of C_{19}-H, one of C_{20}-H, C_{22}-H), 1.23-1.17 (m, 11H, one of C_{19}-H, one of C_{20}-H, $C_{6'}$-H, C_{16}-CH_3, $C_{3'}$-OC-CH_3), 0.97 (m, 1H, C_{11}-H), 0.74 (t, 3H, C_{23}-H); ^{13}C NMR (101 MHz, $CDCl_3$) δ: 201.34, 172.43, 161.63, 149.33, 145.02, 133.78, 133.35, 132.36, 127.75, 95.85, 82.17, 79.62, 78.46, 76.32, 75.72, 67.89, 65.46, 60.86, 59.08, 50.13, 47.85, 46.41, 45.81, 43.33, 40.90, 39.53, 38.74, 38.06, 32.94, 32.58, 30.02, 28.13, 26.91, 24.50, 21.43, 17.76, 16.15, 15.67, 9.32; MS (MALDI) cal for $C_{39}H_{56}O_{10}S$ $[M+Na]^+$ 739.348640, found $[M+ Na]^+$ 739.348619.

Preparation of 8d

Compound **5** (0.34 g, 0.56 mmol), 3-fluorobenzoyl chloride (0.09 g, 0.57 mmol) and DMAP (0.09 g, 0.74 mmol) were added to 15 mL CH_2Cl_2 under Ar, then the mixture was heated to reflux. After about 5 h, the mixture was diluted with CH_2Cl_2 (15 mL) and washed with saturated sodium bicarbonate solution (3 × 10 mL). The combined organic layers were dried with Na_2SO_4 and evaporated under reduced pressure. The residue was purified by column chromatography on silica gel (200–300 mesh) to afford pure **8d** (0.34 g, yield 83%). TLC (ethyl acetate : petroleum ether = 1:3, v:v). ^{1}H NMR (400 MHz, $CDCl_3$) δ: 7.79-7.17 (d, 4H, $C_{3''}$-H, $C_{4''}$-H, $C_{6''}$-H, $C_{7''}$-H), 6.85 (s, 1H, C_{13}-H), 4.76 (s, 1H, $C_{1'}$-H), 4.61 (m, 1H, C_{21}-H), 4.14 (m, 1H, C_9-H), 4.02 (m, 1H, $C_{2'}$-H), 3.64 (m, 1H, C_{17}-H), 3.56-3.49 (m, 7H, $C_{3'}$-OCH_2-, $C_{4'}$-OCH_3, $C_{5'}$-H, C_4-H), 3.43-3.40 (m, 4H, $C_{2'}$-OCH_3, $C_{3'}$-H), 3.11-3.05 (m, 3H, C_{16}-H, one of C_2-H, C_3-H), 2.89 (m, 1H, $C_{4'}$-H), 2.77 (m, 1H, C_{12}-H), 2.50 (s, 1H, one of C_2-H), 2.31 (m, 1H, C_7-H), 2.19 (m, 2H, C_{10}-H), 1.95 (m, 1H, one of C_8-H), 1.73-1.38 (m, 11H, C_5-H, C_6-H, one of C_8-H, C_{18}-H, one of C_{19}-H, one of C_{20}-H, C_{22}-H), 1.24-1.16 (m, 11H, one of C_{19}-H, one of C_{20}-H, $C_{6'}$-H, C_{16}-CH_3, $C_{3'}$-OC-CH_3), 0.94 (m, 1H, C_{11}-H), 0.73 (t, 3H, C_{23}-H); ^{13}C NMR (101 MHz, $CDCl_3$) δ: 201.32, 172.49, 168.92, 163.65, 149.43, 145.00, 130.04, 129.96, 125.72, 116.83, 116.61, 95.74, 82.22, 79.54, 78.45, 76.02, 75.72, 67.85, 65.48, 60.80, 58.98, 50.13, 47.82, 46.38, 45.75, 43.31, 40.88, 39.52, 38.69, 38.00, 34.85, 32.56, 30.01, 28.09, 26.87, 24.45, 21.39, 17.66, 16.14, 15.56, 9.25; MS (MALDI) cal for $C_{41}H_{57}FO_{10}$ $[M+Na]^+$ 751.382797, found $[M+ Na]^+$ 751.382804.

Preparation of 8e

Compound **5** (0.38 g, 0.63 mmol), 2,4-difluorobenzoyl chloride (0.15 g, 0.85 mmol) and DMAP (0.18 g, 1.47 mmol) were added to 15 mL CH_2Cl_2 under Ar, then the mixture was heated to reflux. After about 4 h, the mixture was diluted with CH_2Cl_2 (15 mL) and washed with saturated sodium bicarbonate solution (3 × 10 mL). The combined organic layers were dried with Na_2SO_4 and evaporated under reduced pressure. The residue was purified by column chromatography on silica gel (200–300 mesh) to afford pure **8e** (0.41 g, yield 87%). TLC (ethyl acetate : petroleum ether =

1:4, v:v). ^{1}H NMR (400 MHz, $CDCl_3$) *δ*: 7.92 (m, 1H, $C_{7''}$-H), 6.86-6.76 (m, 3H, $C_{4''}$-H, $C_{6''}$-H, C_{13}-H), 4.74 (s, 1H, $C_{1'}$-H), 4.61 (m, 1H, C_{21}-H), 4.14 (m, 1H, C_9-H), 3.65-3.24 (m, 13H, $C_{2'}$-H, C_{17}-H, $C_{3'}$-OCH_2-, $C_{4'}$-OCH_3, $C_{5'}$-H, C_4-H, $C_{2'}$-OCH_3, $C_{3'}$-H), 3.10-2.90 (m, 3H, C_{16}-H, one of C_2-H, C_3-H), 2.76 (m, 1H, $C_{4'}$-H), 2.51 (s, 1H, one of C_2-H), 2.35-1.93 (m, 3H, C_7-H, C_{10}-H), 1.77-1.44 (m, 13H, C_{12}-H, one of C_8-H, C_5-H, C_6-H, one of C_8-H, C_{18}-H, one of C_{19}-H, one of C_{20}-H, C_{22}-H), 1.19-1.13 (m, 11H, one of C_{19}-H, one of C_{20}-H, $C_{6'}$-H, C_{16}-CH_3, $C_{3'}$-OC-CH_3), 0.95 (m, 1H, C_{11}-H), 0.74 (m, 3H, C_{23}-H); ^{13}C NMR (101 MHz, $CDCl_3$) δ 201.45, 172.53, 166.87, 163.16, 161.51, 149.44, 144.97, 134.02, 133.91, 111.68, 105.22, 95.80, 82.18, 79.65, 78.47, 76.43, 75.66, 67.92, 65.53, 60.96, 59.15, 50.16, 46.49, 45.71, 43.33, 40.92, 39.57, 38.76, 38.07, 33.02, 32.71, 30.12, 28.16, 26.94, 24.53, 21.32, 20.64, 17.79, 16.43, 15.71, 9.37; MS (MALDI) cal for $C_{41}H_{56}F_2O_{10}$ $[M+Na]^+$ 769.373375, found $[M+Na]^+$ 769.373368.

Preparation of 8f

Compound **5** (0.51 g, 0.84 mmol), 4-nitrobenzoyl chloride (0.21 g, 1.13 mmol) and DMAP (0.19 g, 1.56 mmol) were added to 15 mL CH_2Cl_2 under Ar, then the mixture was heated to reflux. After about 2 h, the mixture was diluted with CH_2Cl_2 (15 mL) and washed with saturated sodium bicarbonate solution (3 × 10 mL). The combined organic layers were dried with Na_2SO_4 and evaporated under reduced pressure. The residue was purified by column chromatography on silica gel (200–300 mesh) to afford pure **8f** (0.56 g, yield 89%). TLC (ethyl acetate : petroleum ether = 1:3, v:v). ^{1}H NMR (400 MHz, $CDCl_3$) *δ*: 8.32-8.24 (m, 4H, $C_{3''}$-H, $C_{4''}$-H, $C_{6''}$-H, $C_{7''}$-H), 6.97 (s, 1H, C_{13}-H), 4.83 (s, 1H, $C_{1'}$-H), 4.72 (m, 1H, C_{21}-H), 4.24 (m, 1H, C_9-H), 3.74-3.47 (m, 13H, $C_{2'}$-H, C_{17}-H, $C_{3'}$-OCH_2-, $C_{4'}$-OCH_3, $C_{5'}$-H, C_4-H, $C_{2'}$-OCH_3, $C_{3'}$-H), 3.20-3.00 (m, 3H, C_{16}-H, one of C_2-H, C_3-H), 2.89 (m, 1H, $C_{4'}$-H), 2.61 (m, 1H, C_{12}-H), 2.42-2.21 (m, 3H, one of C_2-H, C_{10}-H), 1.89-1.49 (m, 13H, C_7-H, C_8-H, C_5-H, C_6-H, C_{18}-H, one of C_{19}-H, one of C_{20}-H, C_{22}-H), 1.27-1.20 (m, 11H, one of C_{19}-H, one of C_{20}-H, $C_{6'}$-H, C_{16}-CH_3, $C_{3'}$-OC-CH_3), 1.08 (m, 1H, C_{11}-H), 0.83 (t, *J*=7.2 Hz, 3H, C_{23}-H); ^{13}C NMR (101 MHz, $CDCl_3$) δ 200.99, 172.36, 164.03, 150.48, 149.48, 144.99, 135.58, 130.65, 123.50, 95.78, 82.11, 79.65, 78.40, 76.77, 76.20, 75.55, 67.86,

65.44, 60.89, 59.10, 50.14, 46.45, 45.65, 43.26, 40.90, 39.51, 38.73, 38.01, 32.85, 32.52, 30.04, 28.15, 26.91, 24.47, 22.59, 21.44, 20.61, 17.77, 16.20, 15.69, 9.35; MS (MALDI) cal for $C_{41}H_{57}NO_{12}$ $[M+Na]^+$ 778.377297, found $[M+ Na]^+$ 778.377181.

Preparation of 8g

Compound **5** (0.38 g, 0.63 mmol), 3-methylbenzoyl chloride (0.13 g, 0.84 mmol) and DMAP (0.14 g, 1.14 mmol) were added to 15 mL CH_2Cl_2 under Ar, then the mixture was heated to reflux. After about 9 h, the mixture was diluted with CH_2Cl_2 (15 mL) and washed with saturated sodium bicarbonate solution (3 × 10 mL). The combined organic layers were dried with Na_2SO_4 and evaporated under reduced pressure. The residue was purified by column chromatography on silica gel (200–300 mesh) to afford pure **8g** (0.37 g, yield 81%). TLC (ethyl acetate : petroleum ether = 1:3, v:v). ^{1}H NMR (400 MHz, $CDCl_3$) δ: 7.84 (d, 1H, $C_{7''}$-H); 7.79 (s, 1H, $C_{3''}$-H), 7.30-7.25 (m, 2H, $C_{5''}$-H, $C_{6''}$-H), 6.84 (s, 1H, C_{13}-H), 4.74 (s, 1H, $C_{1'}$-H), 4.61 (m, 1H, C_{21}-H), 4.13 (m, 1H, C_9-H), 3.65 (m, 1H, C_{17}-H), 3.57-3.38 (m, 12H, $C_{2'}$-H, $C_{3'}$-OCH_2-, $C_{4'}$-OCH_3, $C_{5'}$-H, C_4-H, $C_{2'}$-OCH_3, $C_{3'}$-H), 3.12-3.03 (m, 2H, C_{16}-H, one of C_2-H), 2.90 (m, 1H, $C_{4'}$-H), 2.78 (m, 1H, C_{12}-H), 2.50 (m, 1H, one of C_2-H), 2.33-2.28 (m, 6H, C_7-H, C_3-H, $C_{4''}$-CH_3, one of C_8-H), 2.17 (m, 2H, C_{10}-H), 1.78-1.40 (m, 11H, C_5-H, C_6-H, one of C_8-H, C_{18}-H, one of C_{19}-H, one of C_{20}-H, C_{22}-H), 1.20-1.10 (m, 11H, one of C_{19}-H, one of C_{20}-H, $C_{6'}$-H, C_{16}-CH_3, $C_{3'}$-OC-CH_3), 0.94 (m, 1H, C_{11}-H), 0.74 (t, J=7.2 Hz, 3H, C_{23}-H); ^{13}C NMR (101 MHz, $CDCl_3$) δ 200.63, 171.60, 165.18, 148.33, 144.15, 137.16, 132.70, 129.61, 129.11, 127.29, 126.26, 125.73, 94.80, 81.22, 78.66, 77.49, 74.68, 74.44, 66.94, 64.56, 60.00, 58.17, 49.16, 45.50, 44.92, 42.35, 39.96, 38.56, 37.79, 37.08, 32.00, 31.68, 29.07, 27.22, 25.96, 23.54, 20.56, 20.25, 16.82, 15.26, 14.74, 8.41; MS (MALDI) cal for $C_{42}H_{60}O_{10}$ $[M+Na]^+$ 747.407896, found $[M+ Na]^+$ 747.407790.

Preparation of 8h

Compound **5** (0.34 g, 0.56 mmol), 4-cyanobenzoyl chloride (0.12 g, 0.72 mmol) and DMAP (0.15 g, 1.23 mmol) were added to 15 mL CH_2Cl_2 under Ar, then the mixture was heated to reflux. After about 2 h, the mixture was diluted with CH_2Cl_2 (15 mL) and washed with saturated sodium bicarbonate solution (3 × 10 mL). The combined

organic layers were dried with Na_2SO_4 and evaporated under reduced pressure. The residue was purified by column chromatography on silica gel (200–300 mesh) to afford pure **8h** (0.37 g, yield 90%). TLC (ethyl acetate : petroleum ether = 2:5, v:v). ^{1}H NMR (400 MHz, $CDCl_3$) δ: 8.18 (d, 2H, $C_{3''}$-H, $C_{7''}$-H), 7.79 (d, 2H, $C_{4''}$-H, $C_{6''}$-H), 6.95 (s, 1H, C_{13}-H), 4.83 (s, 1H, $C_{1'}$-H), 4.71 (m, 1H, C_{21}-H), 4.24 (m, 1H, C_9-H), 3.75-3.47 (m, 13H, $C_{2'}$-H, C_{17}-H, $C_{3'}$-OCH_2-, $C_{4'}$-OCH_3, $C_{5'}$-H, C_4-H), 3.43-3.40 (m, 4H, $C_{2'}$-OCH_3, $C_{3'}$-H), 3.20-3.10 (m, 2H, C_{16}-H, one of C_2-H), 2.99 (m, 1H, C_3-H), 2.88 (m, 1H, $C_{4'}$-H), 2.59 (m, 1H, C_{12}-H), 2.48-2.04 (m, 4H, one of C_2-H, C_7-H, C_{10}-H), 1.84-1.50 (m, 12H, one of C_8-H, C_5-H, C_6-H, one of C_8-H, C_{18}-H, one of C_{19}-H, one of C_{20}-H, C_{22}-H), 1.28-1.19 (m, 11H, one of C_{19}-H, one of C_{20}-H, $C_{6'}$-H, C_{16}-CH_3, $C_{3'}$-OC-CH_3), 1.05 (m, 1H, C_{11}-H), 0.83 (t, J=7.6 Hz, 3H, C_{23}-H); ^{13}C NMR (101 MHz, $CDCl_3$) δ 201.00, 172.33, 164.26, 149.41, 144.99, 134.01, 132.20, 130.05, 117.80, 116.36, 95.87, 95.79, 82.10, 79.66, 78.39, 76.56, 76.18, 75.55, 67.85, 65.42, 60.88, 59.09, 50.12, 46.43, 45.65, 43.25, 40.90, 39.49, 38.73, 32.51, 31.81, 30.03, 28.14, 26.90, 24.46, 22.58, 21.44, 17.77, 16.18, 15.69, 14.05, 9.35; MS (MALDI) cal for $C_{42}H_{57}NO_{10}$ $[M+Na]^+$ 758.387468, found $[M+ Na]^+$ 758.387492.

Preparation of 8i

Compound **5** (0.41 g, 0.68 mmol), 4-methoxybenzoyl chloride (0.15 g, 0.88 mmol) and DMAP (0.16 g, 1.31 mmol) were added to 15 mL CH_2Cl_2 under Ar, then the mixture was heated to reflux. After about 8 h, the mixture was diluted with CH_2Cl_2 (15 mL) and washed with saturated sodium bicarbonate solution (3 × 10 mL). The combined organic layers were dried with Na_2SO_4 and evaporated under reduced pressure. The residue was purified by column chromatography on silica gel (200–300 mesh) to afford pure **8i** (0.40 g, yield 79%). TLC (ethyl acetate : petroleum ether = 1:3, v:v). ^{1}H NMR (400 MHz, $CDCl_3$) δ: 8.01 (d, 2H, $C_{3''}$-H, $C_{7''}$-H), 6.94-6.92 (m, 3H, $C_{4''}$-H, $C_{6''}$-H, C_{13}-H), 4.82 (s, 1H, $C_{1'}$-H), 4.70 (m, 1H, C_{21}-H), 4.23 (m, 1H, C_9-H), 3.86 (s, 3H, $C_{5''}$-OCH_3), 3.75-3.46 (m, 13H, $C_{2'}$-H, C_{17}-H, $C_{3'}$-OCH_2-, $C_{4'}$-OCH_3, $C_{5'}$-H, C_4-H, $C_{2'}$-OCH_3, $C_{3'}$-H), 3.21-2.98 (m, 3H, C_{16}-H, one of C_2-H, C_3-H), 2.87 (m, 1H, $C_{4'}$-H), 2.58 (m, 1H, C_{12}-H), 2.42-2.16 (m, 3H, one of C_2-H, C_7-H, one of C_{10}-H), 1.87-1.42 (m, 13H, one of C_{10}-H, one of C_8-H, C_5-H, C_6-H, one of C_8-H, C_{18}-H, one

of C_{19}-H, one of C_{20}-H, C_{22}-H), 1.28-1.17 (m, 11H, one of C_{19}-H, one of C_{20}-H, $C_{6'}$-H, C_{16}-CH_3, $C_{3'}$-OC-CH_3), 1.06 (m, 1H, C_{11}-H), 0.82 (t, J=7.6Hz, 3H, C_{23}-H); ^{13}C NMR (101 MHz, $CDCl_3$) δ 201.56, 172.46, 165.67, 163.35, 149.15, 145.13, 131.54, 122.63, 113.59, 95.80, 82.14, 79.67, 78.44, 76.36, 75.63, 75.04, 67.89, 65.47, 60.92, 59.13, 55.36, 53.46, 50.12, 46.45, 45.92, 43.31, 40.92, 39.52, 38.76, 38.04, 32.92, 32.66, 30.07, 28.19, 26.93, 24.52, 22.62, 21.53, 17.80, 16.17, 15.72, 9.38; MS (MALDI) cal for $C_{42}H_{60}O_{11}$ $[M+Na]^+$ 763.402783, found $[M+ Na]^+$ 763.402919.

Analytical data

^{1}H NMR of **1**

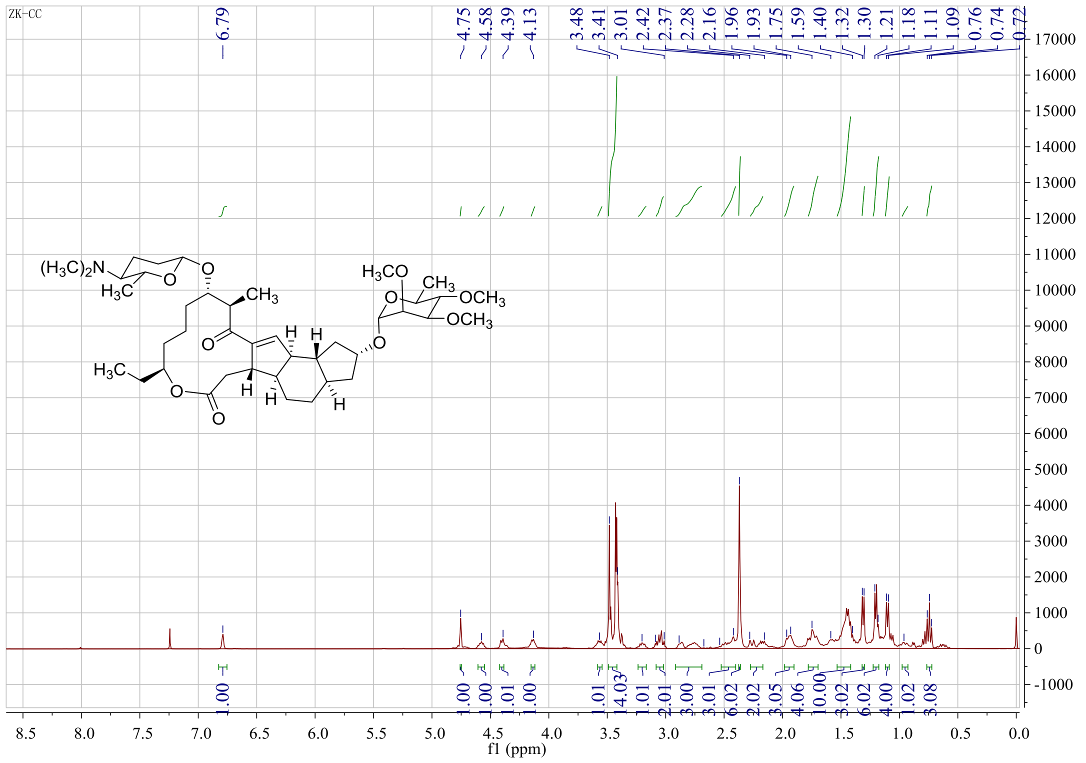

^{13}C NMR of **1**

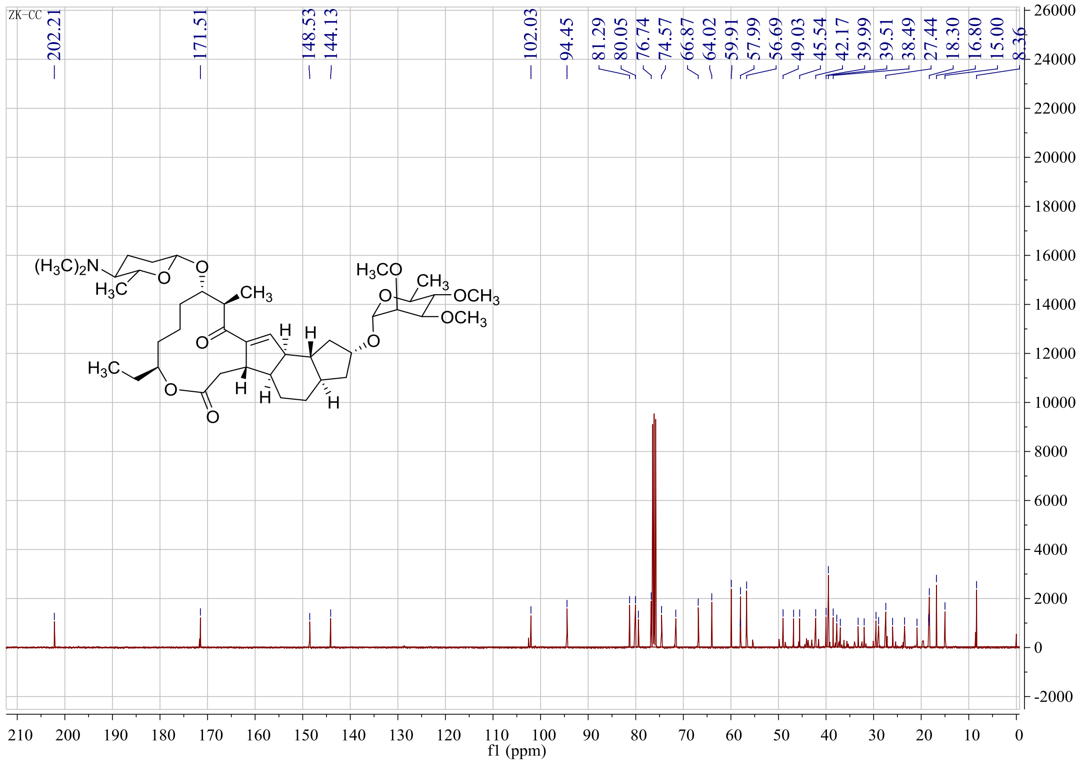

^{1}H NMR of **2**

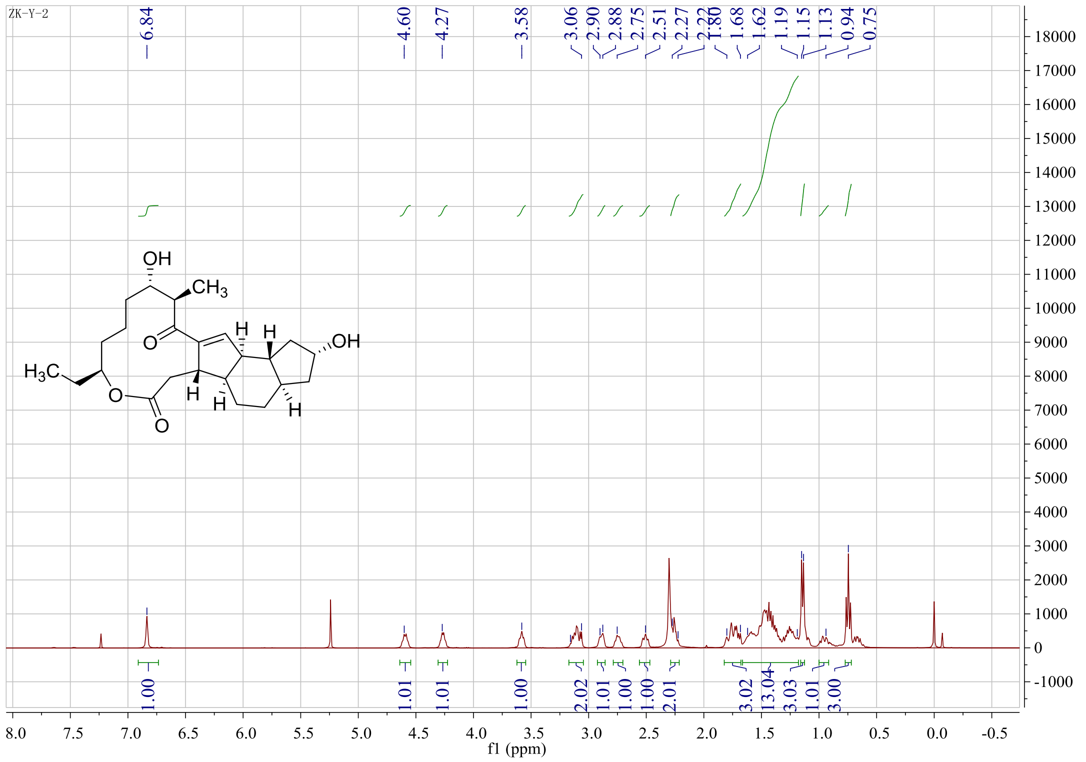

^{13}C NMR of **2**

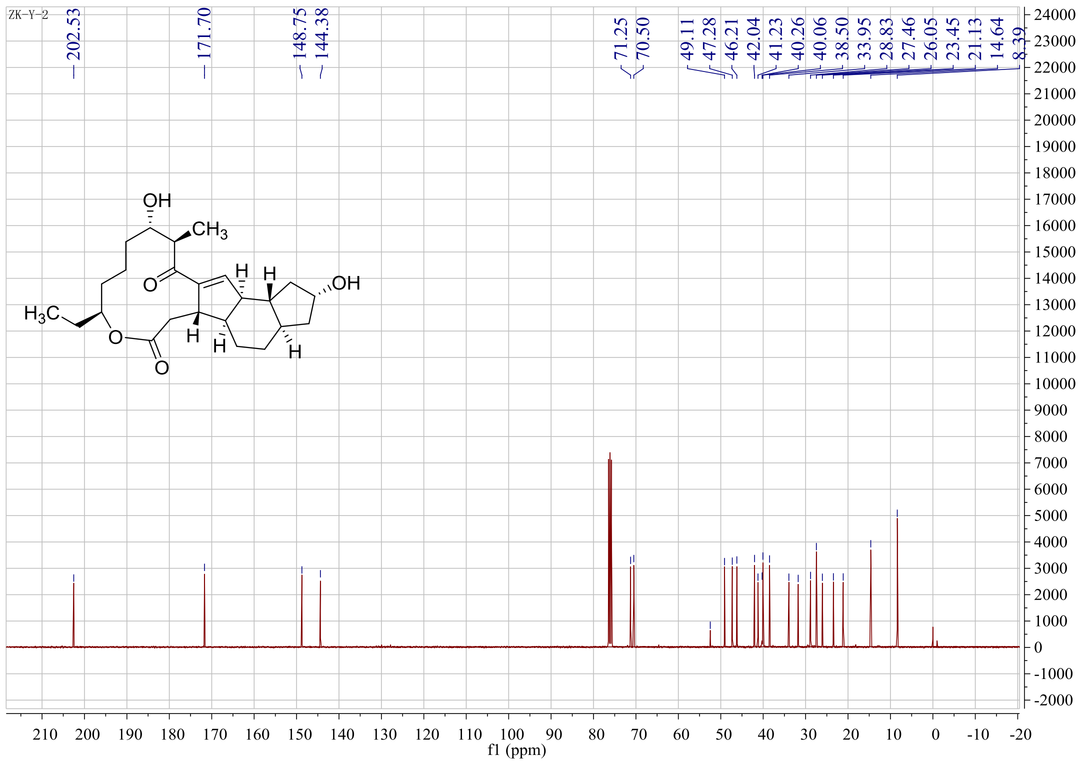

^{1}H NMR of **4**

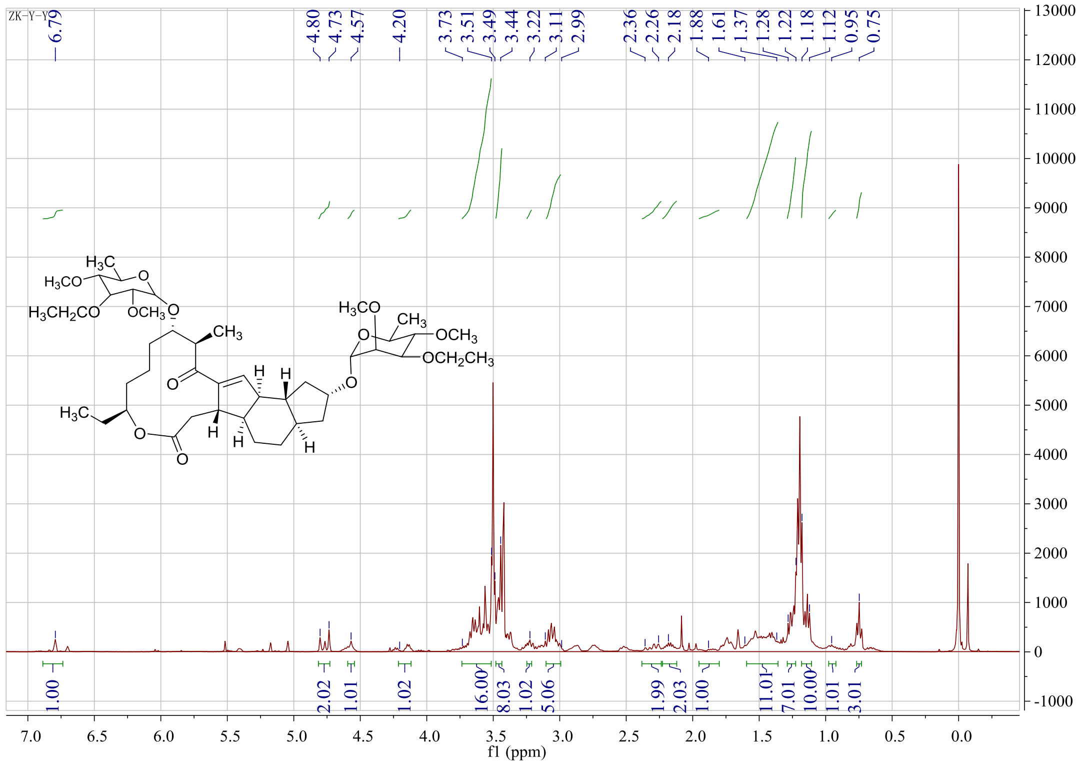

^{13}C NMR of **4**

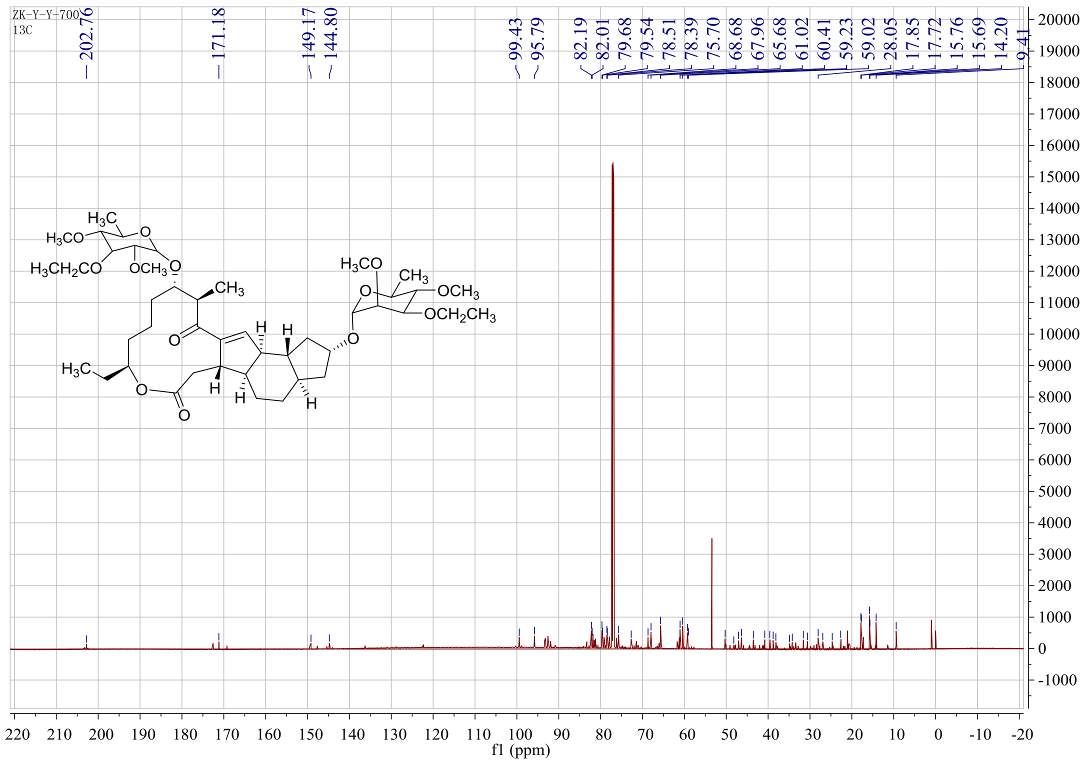

^{1}H NMR of **5**

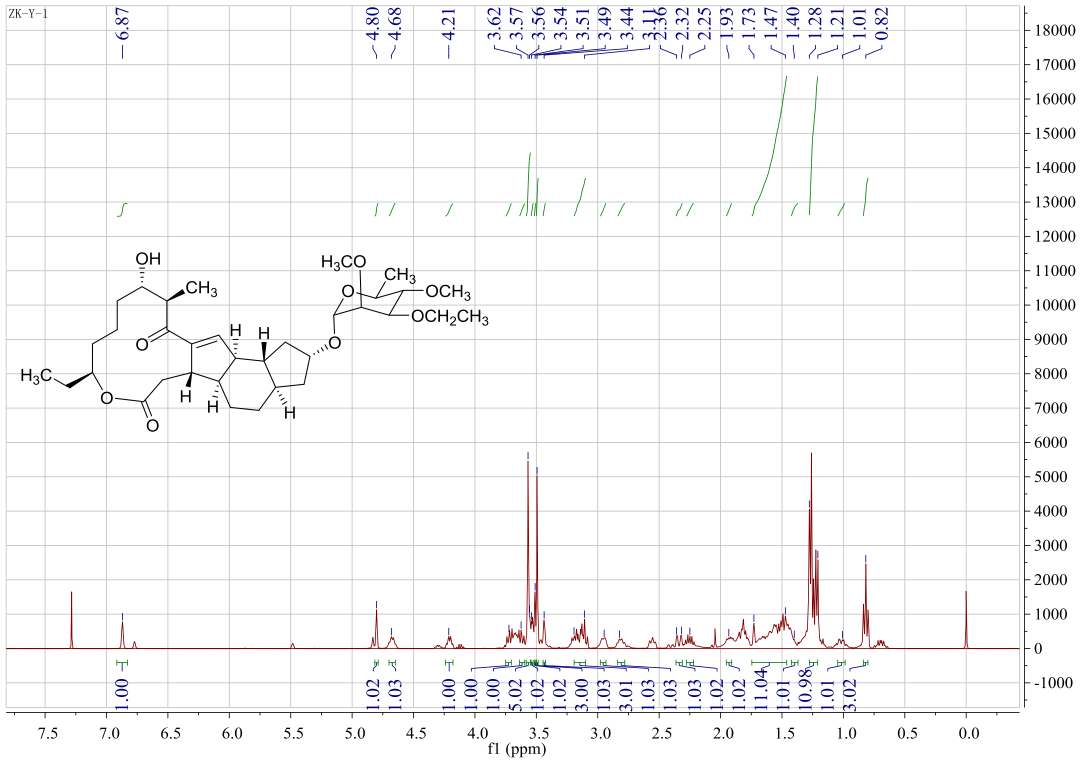

^{13}C NMR of **5**

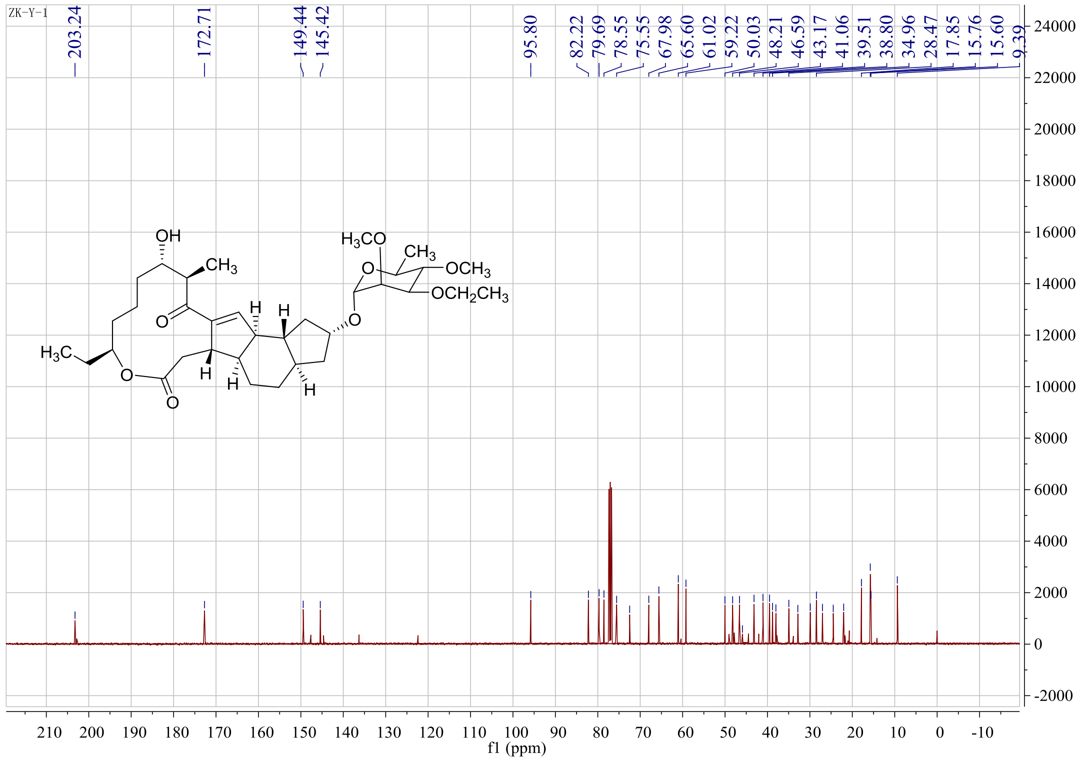

^{1}H NMR of **7a**

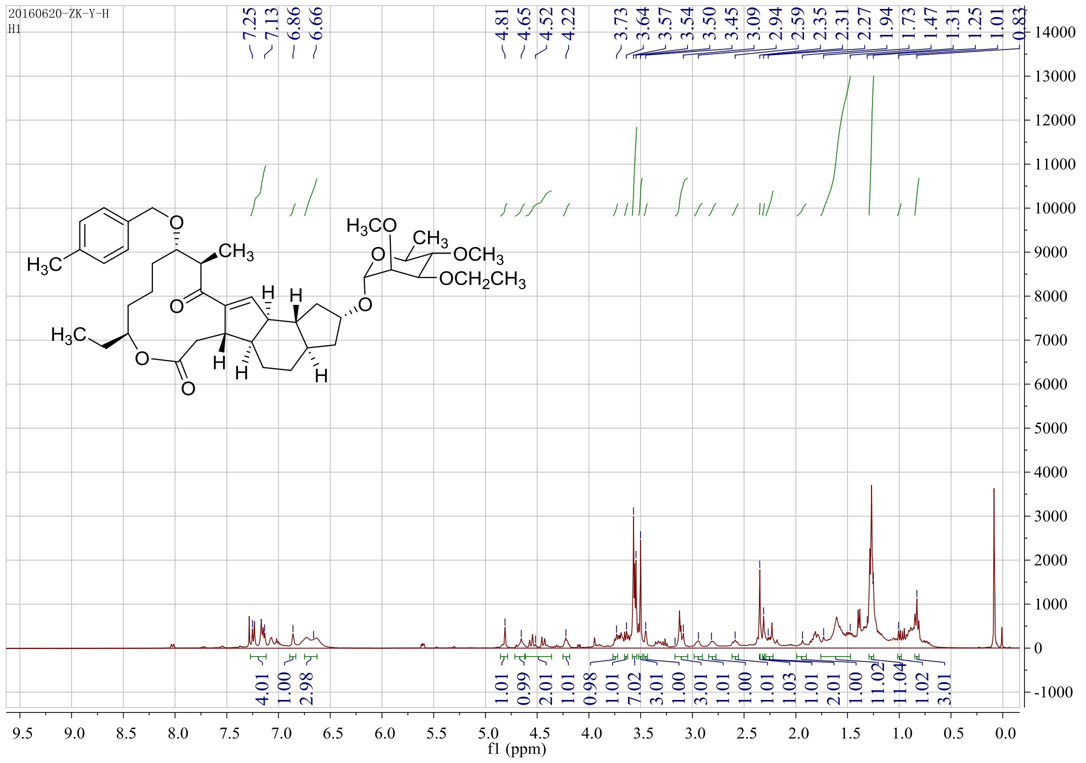

^{13}C NMR of **7a**

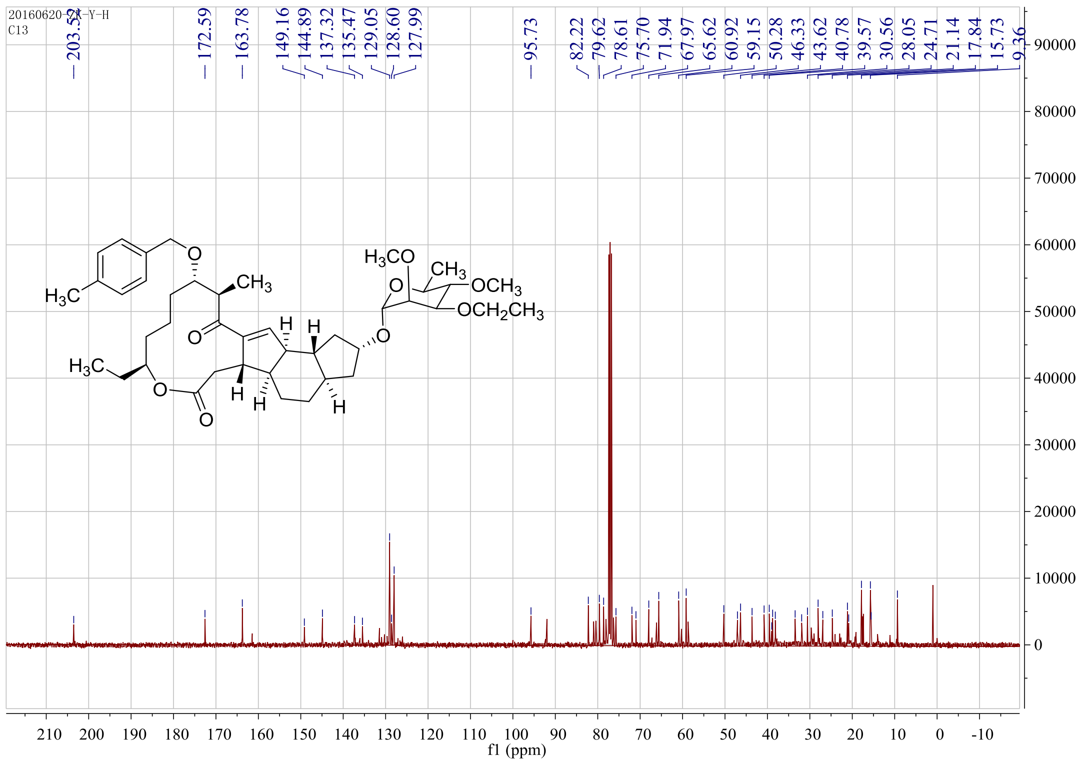

^{1}H NMR of **7b**

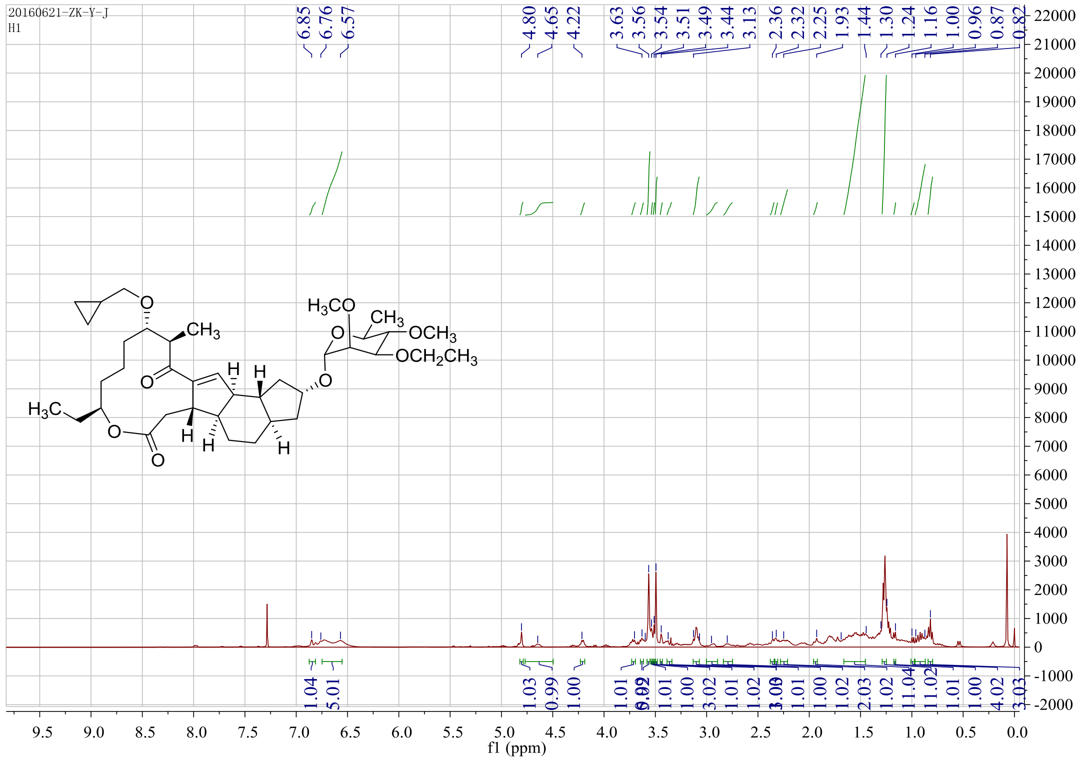

^{13}C NMR of **7b**

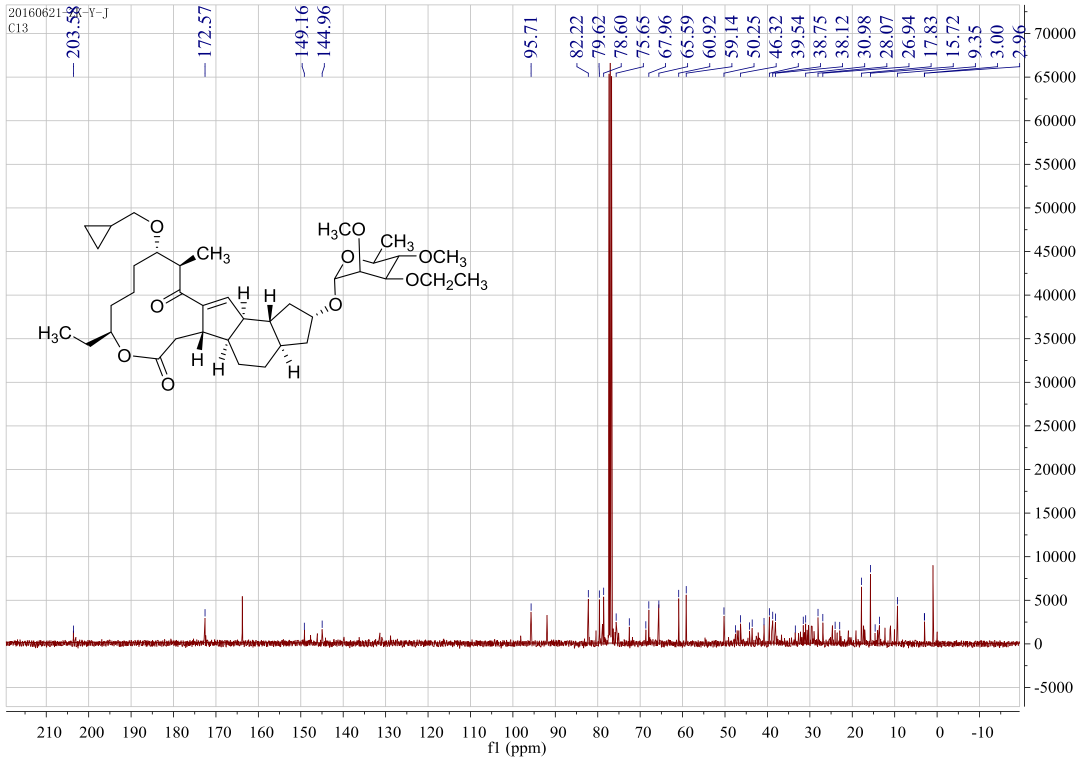

^{1}H NMR of **7c**

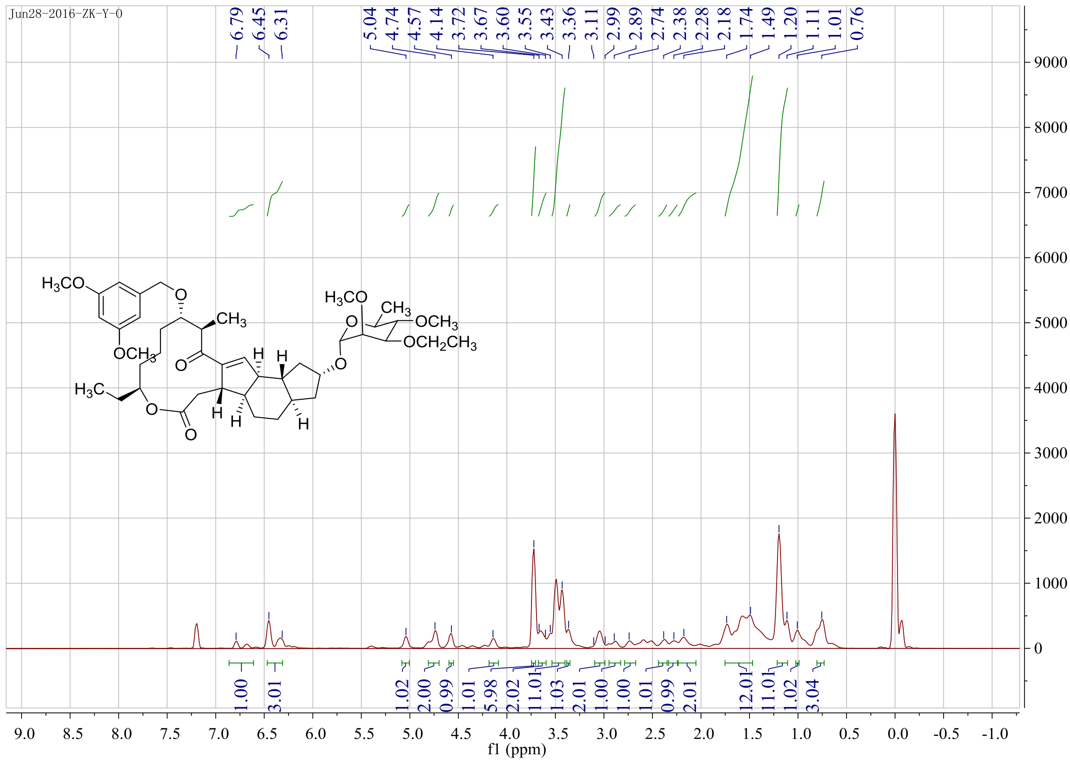

^{13}C NMR of **7c**

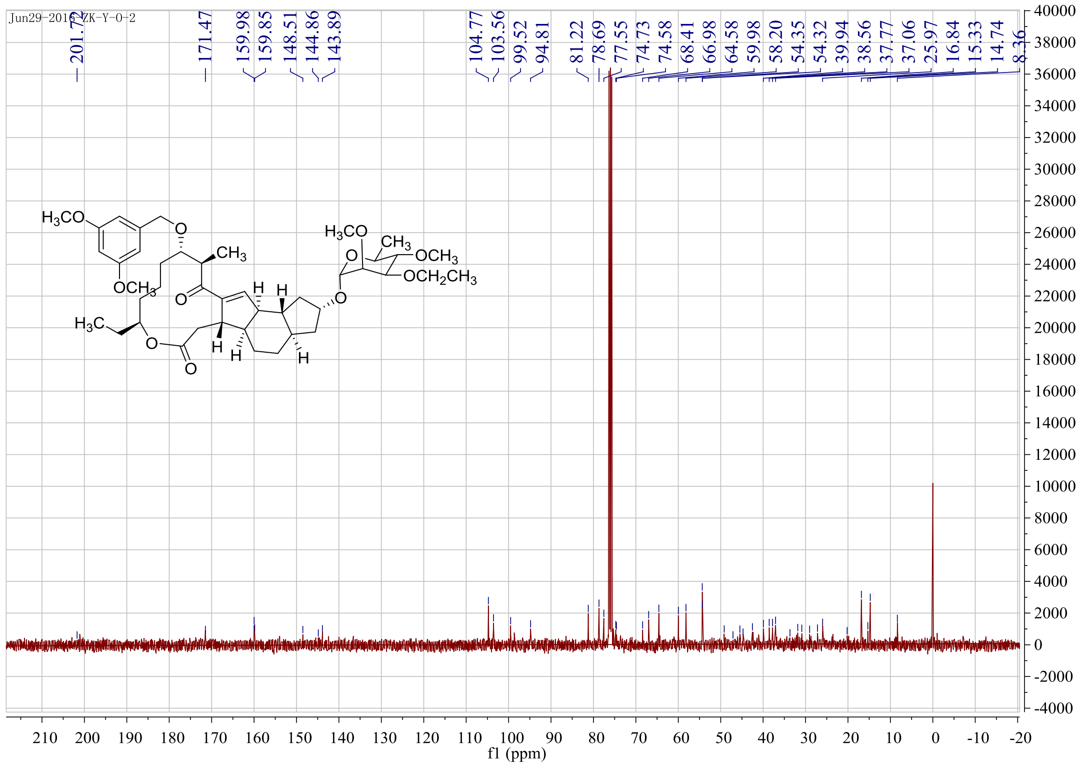

^{1}H NMR of **7d**

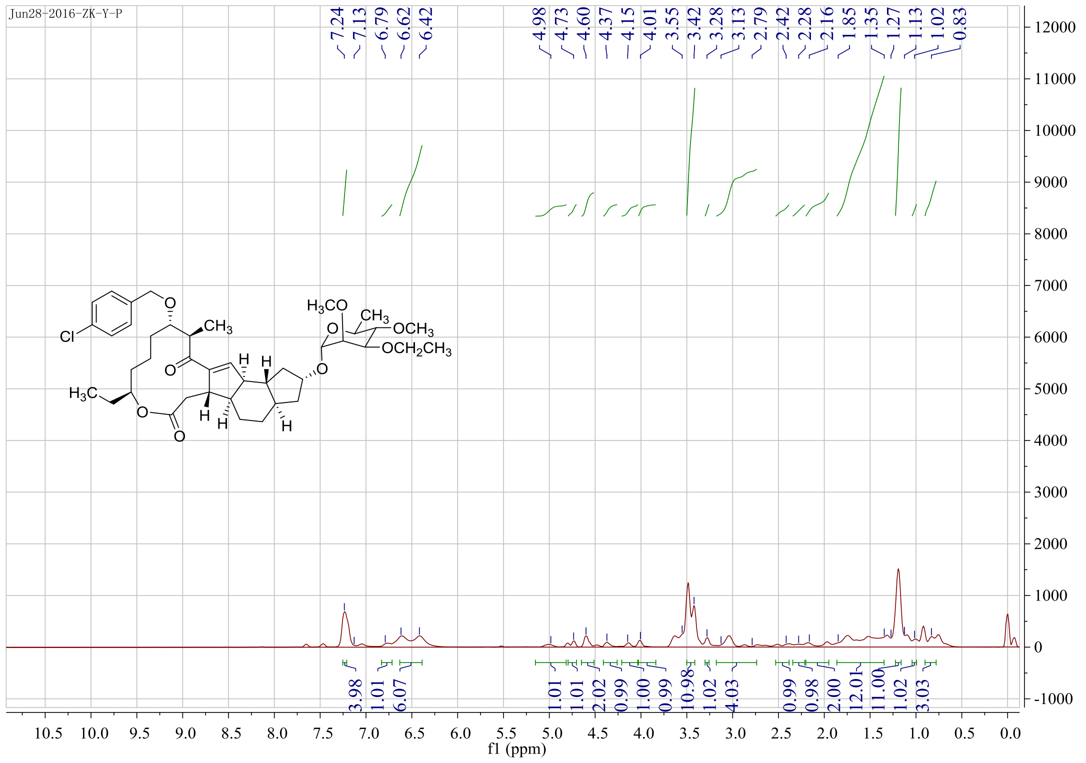

^{13}C NMR of **7d**

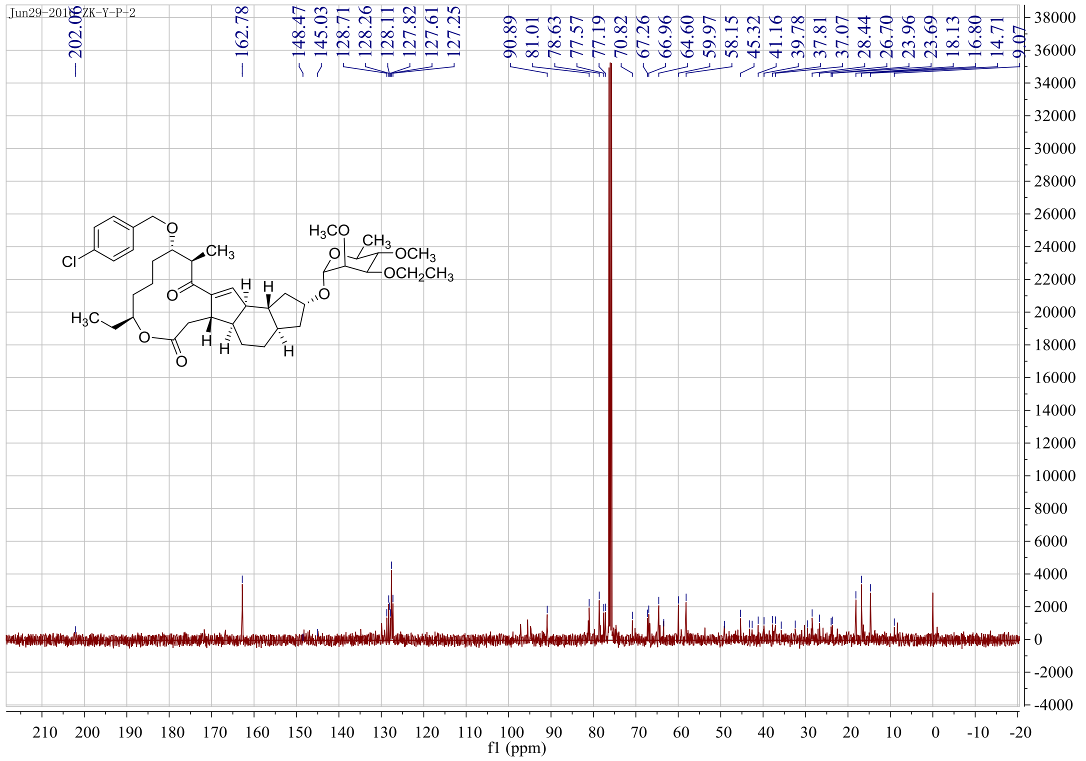

^{1}H NMR of **7e**

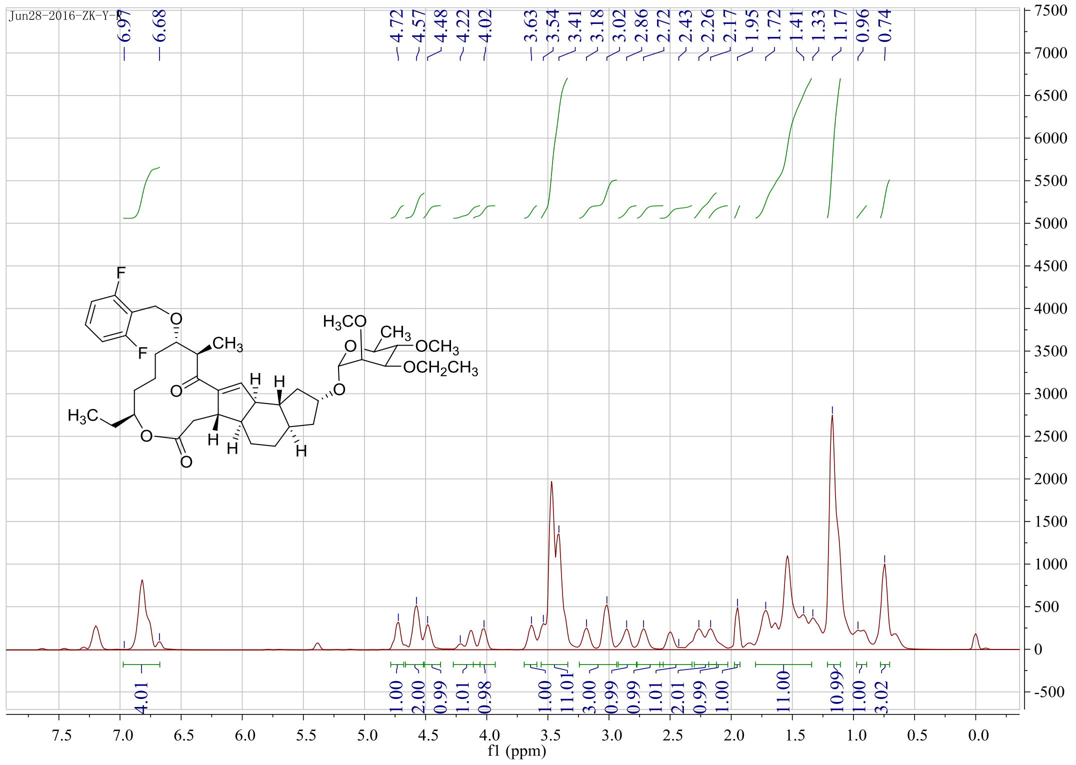

^{13}C NMR of **7e**

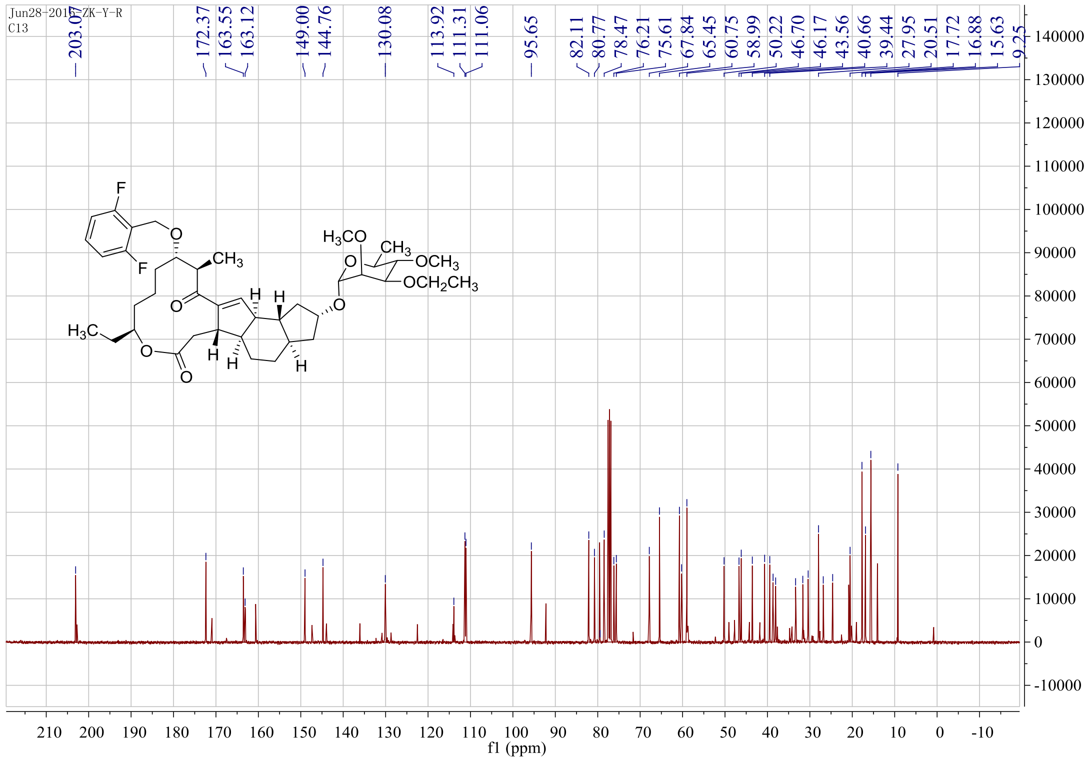

^{1}H NMR of **7f**

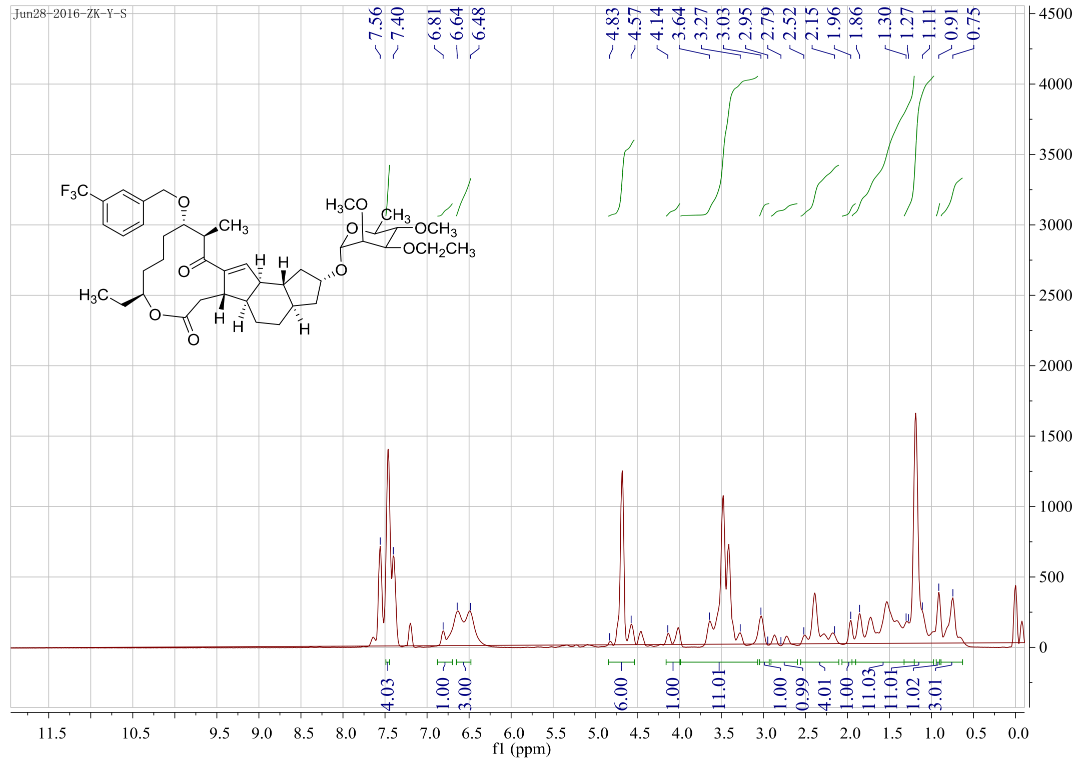

^{13}C NMR of **7f**

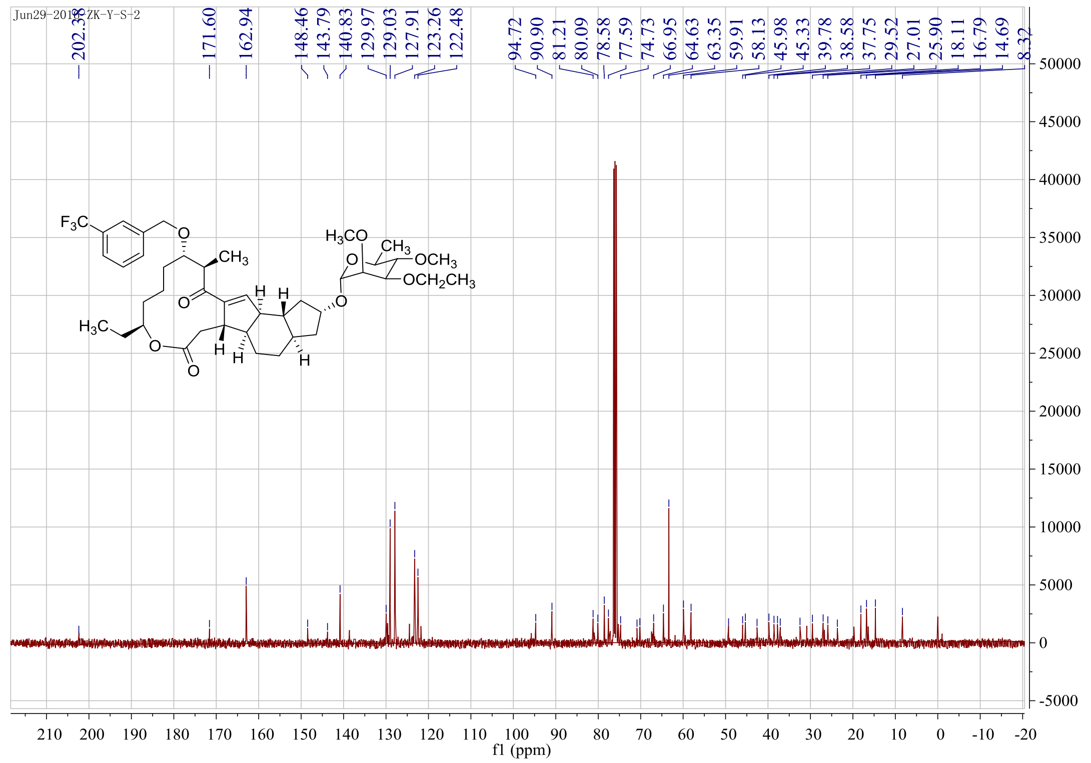

^{1}H NMR of **7g**

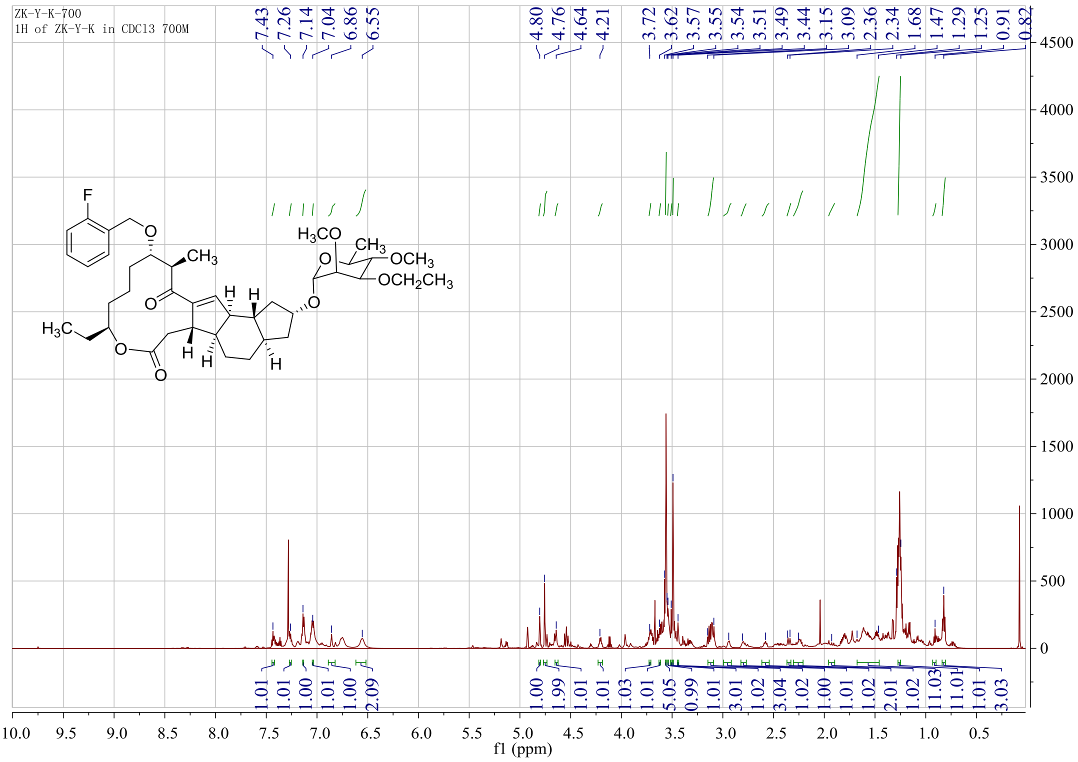

^{13}C NMR of **7g**

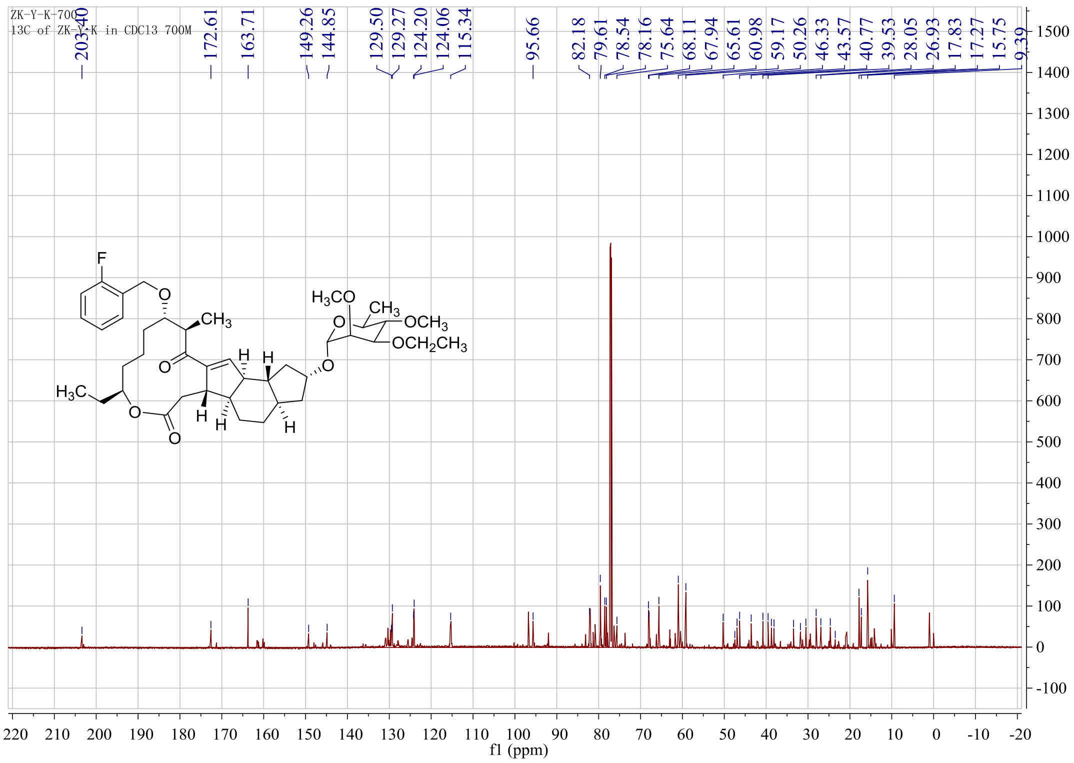

^{1}H NMR of **7h**

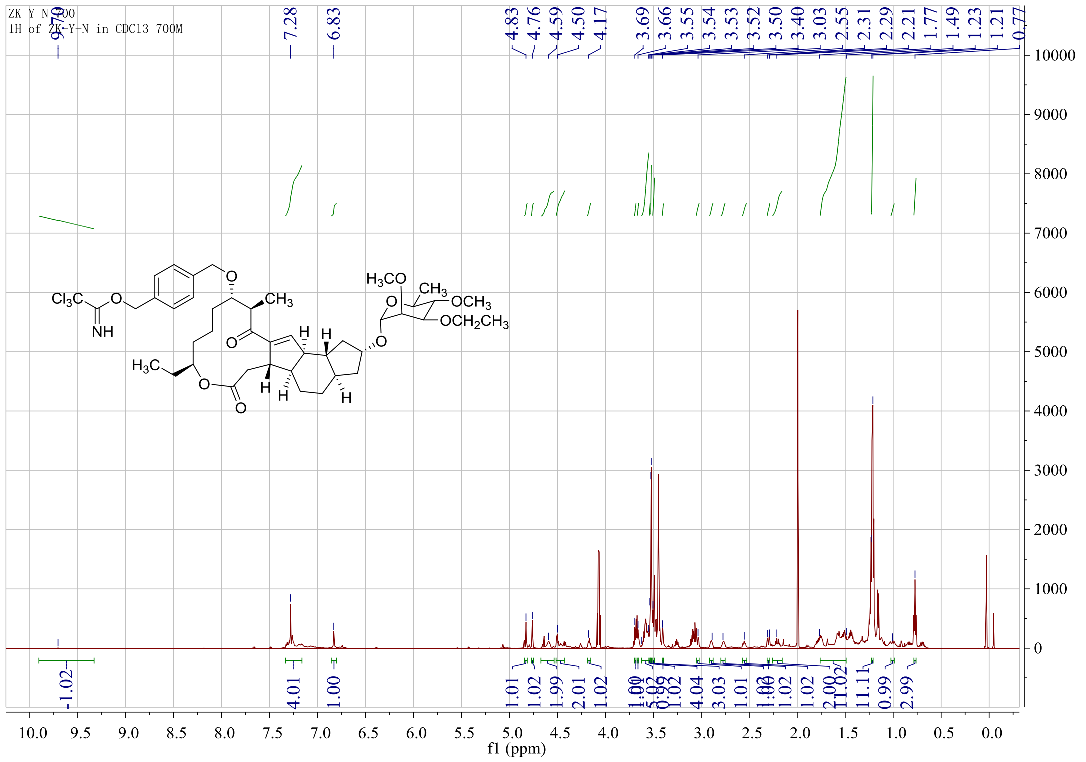

^{13}C NMR of **7h**

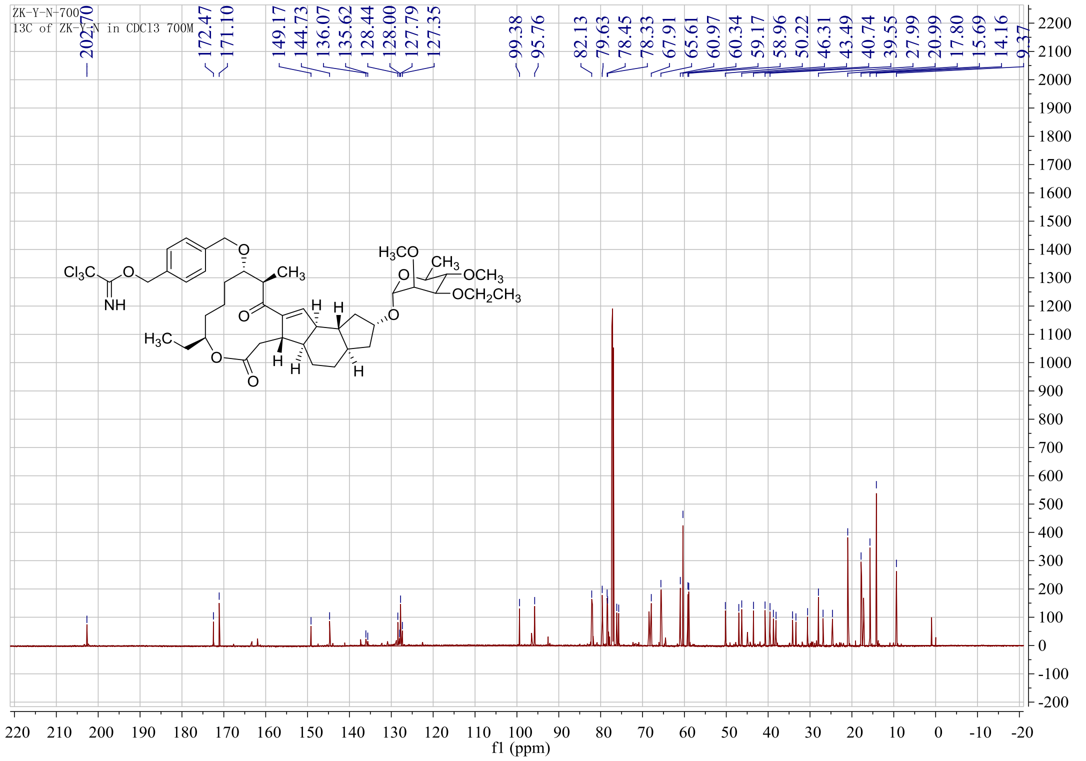

^{1}H NMR of **7i**

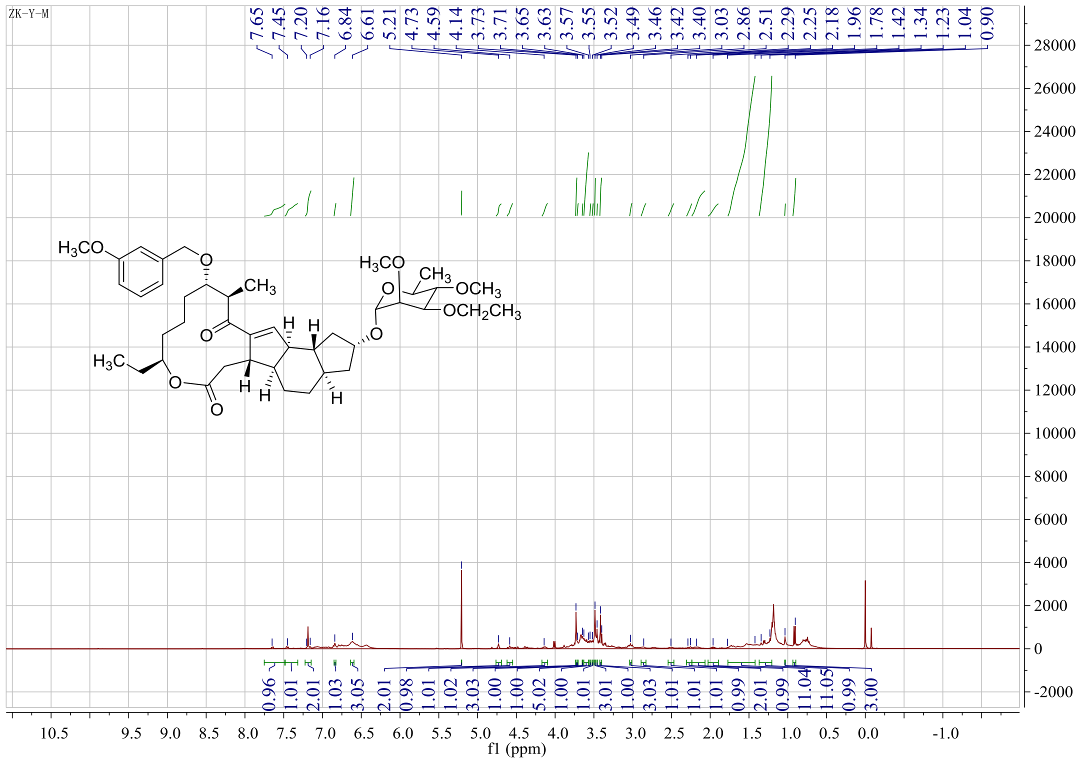

^{13}C NMR of **7i**

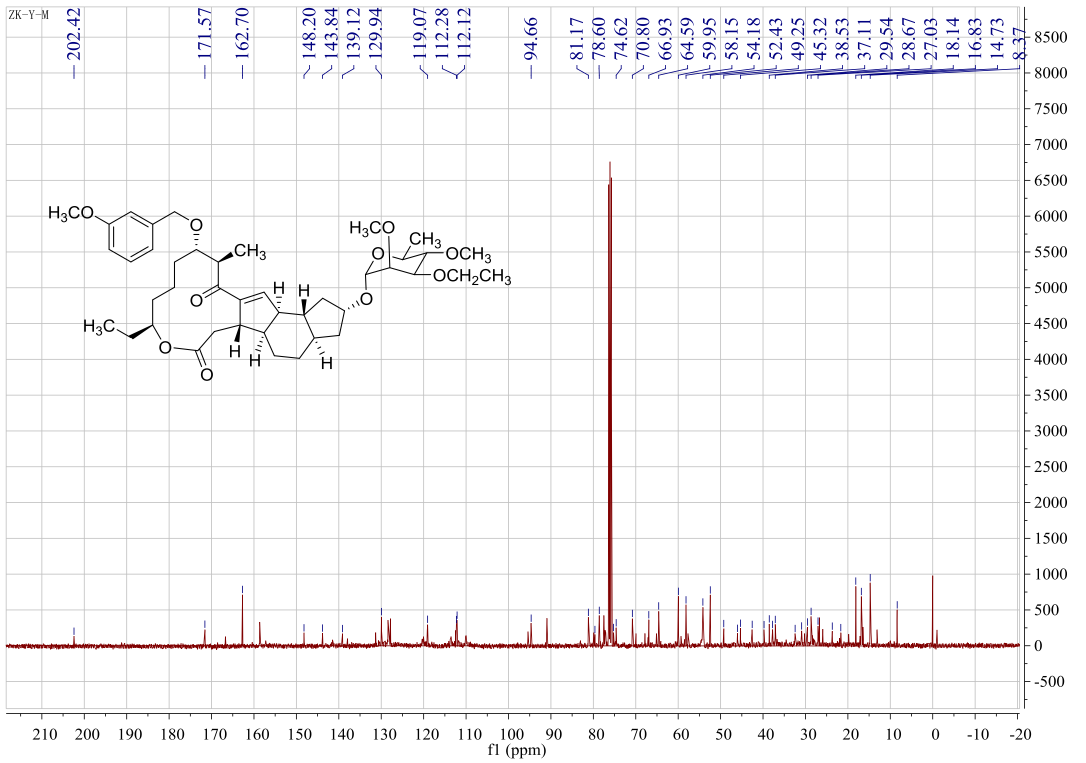

^{1}H NMR of **7j**

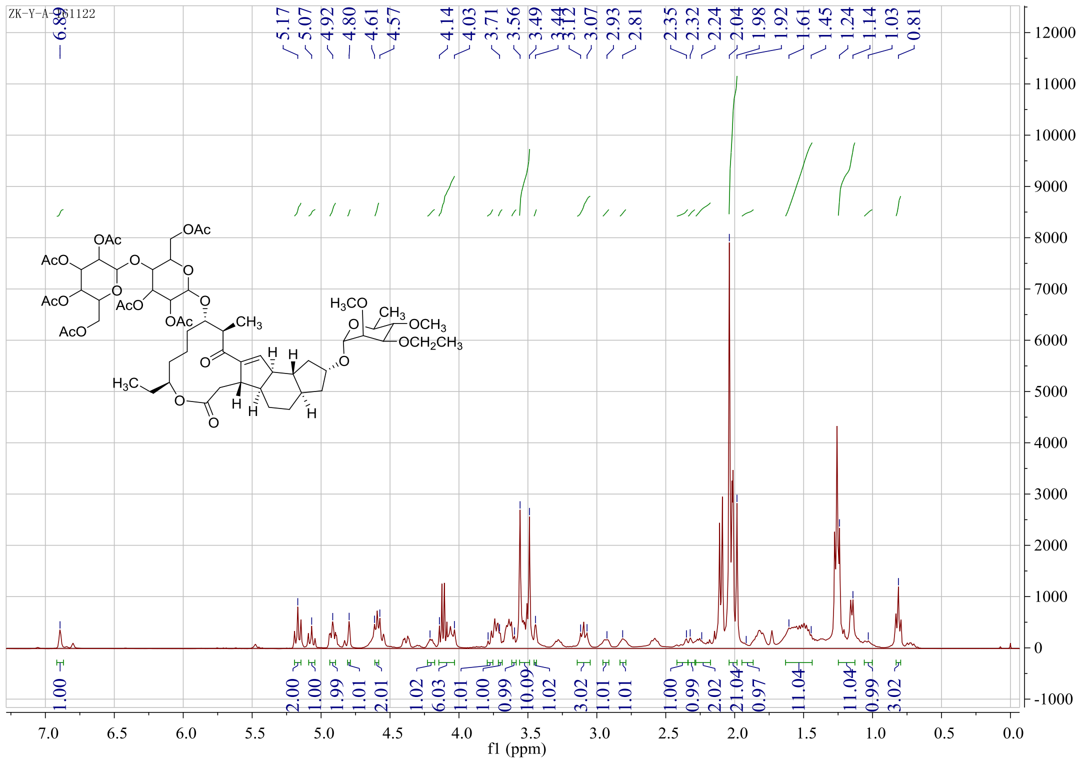

^{13}C NMR of **7j**

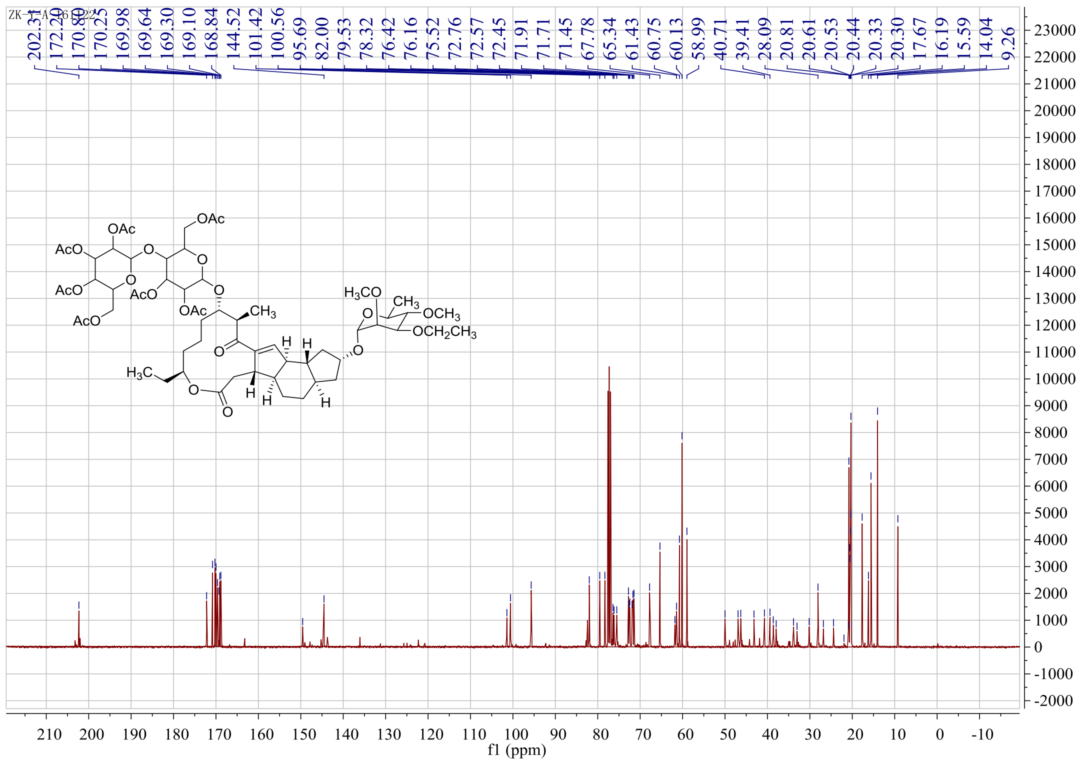

^{1}H NMR of **7k**

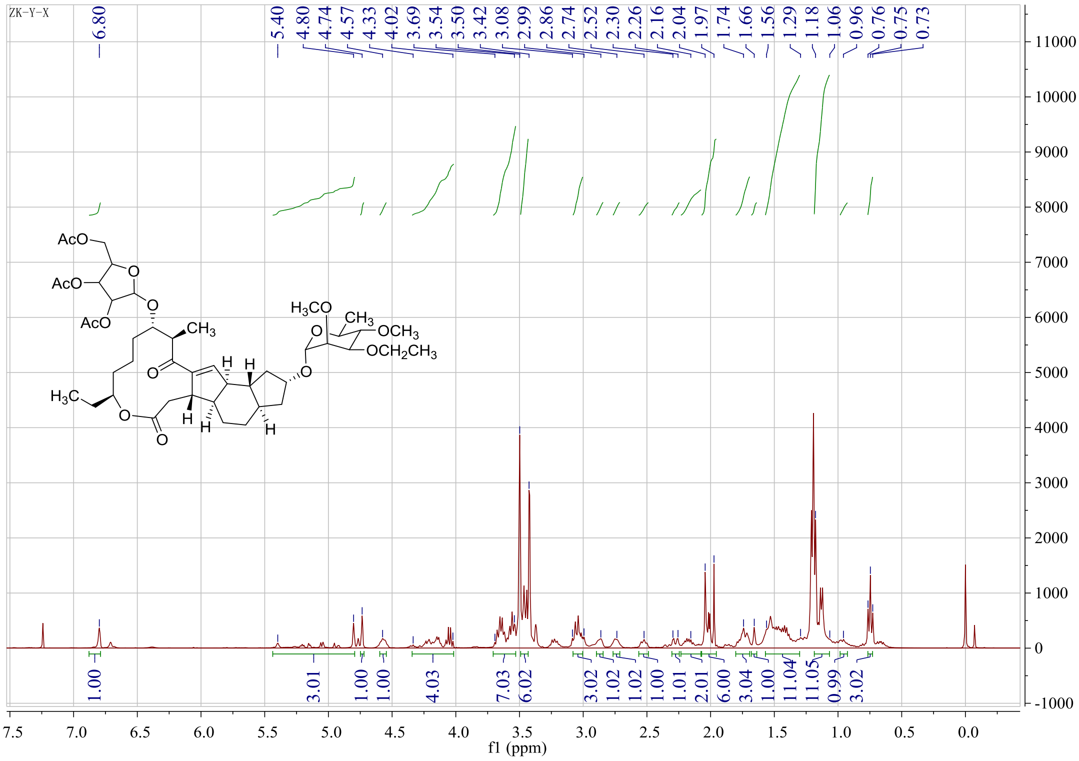

^{13}C NMR of **7k**

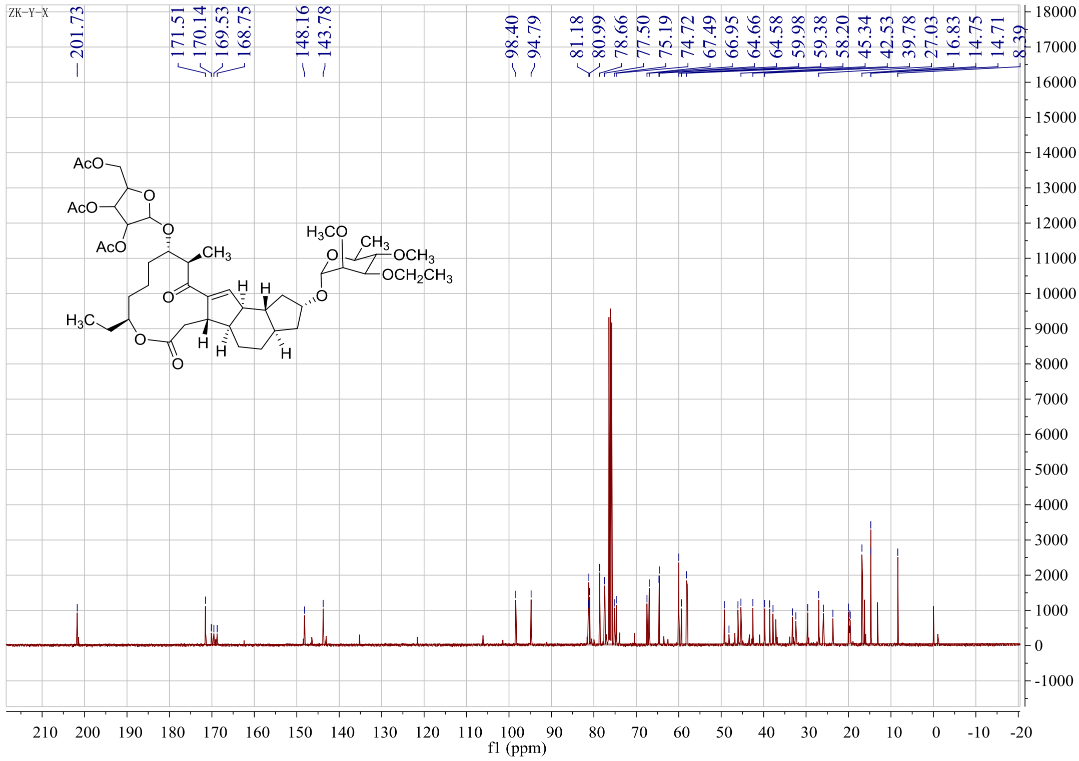

^{1}H NMR of **7l**

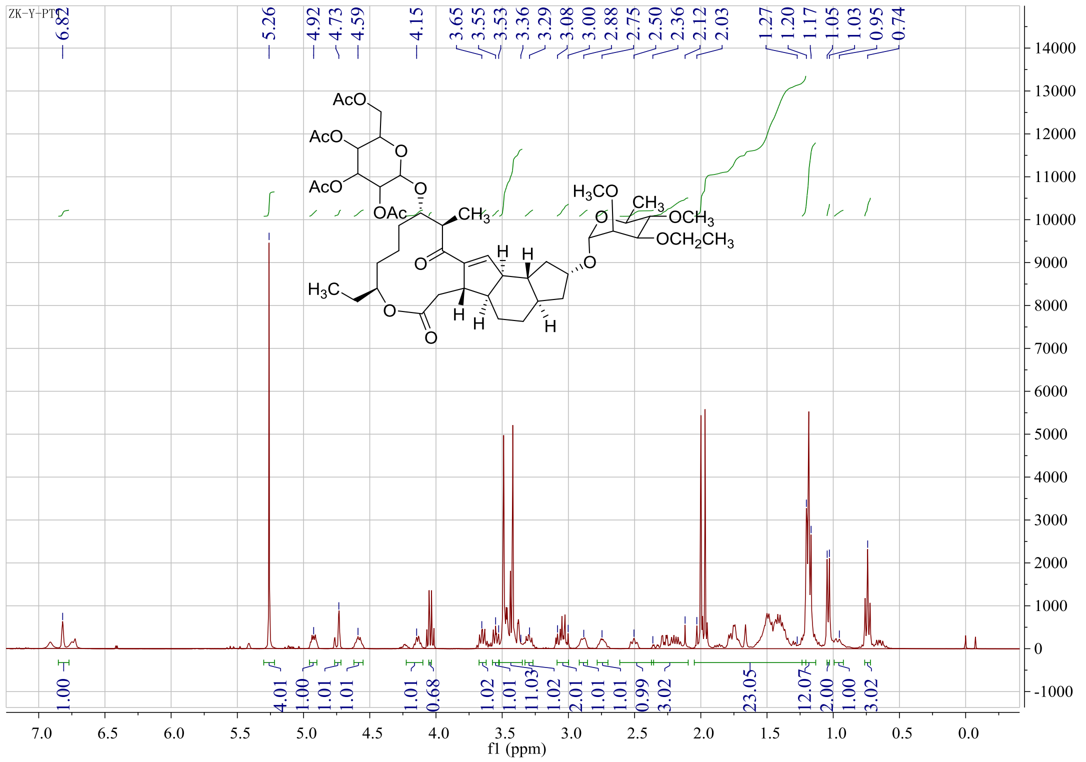

^{13}C NMR of **7l**

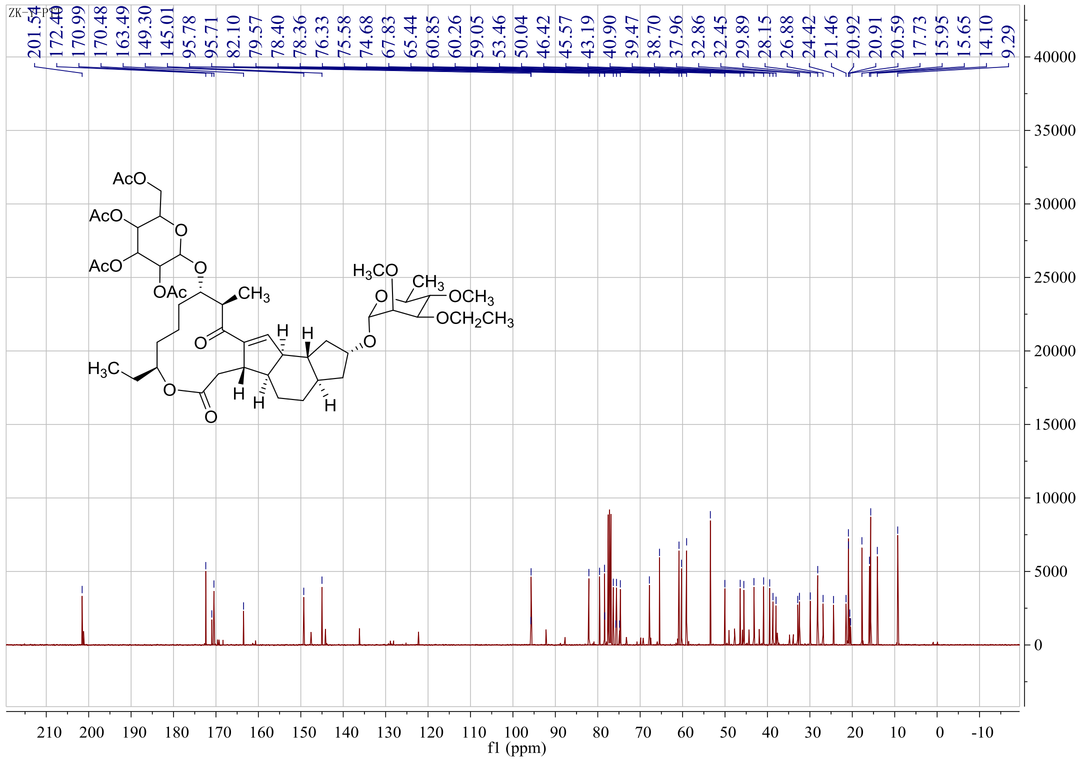

^{1}H NMR of **8a**

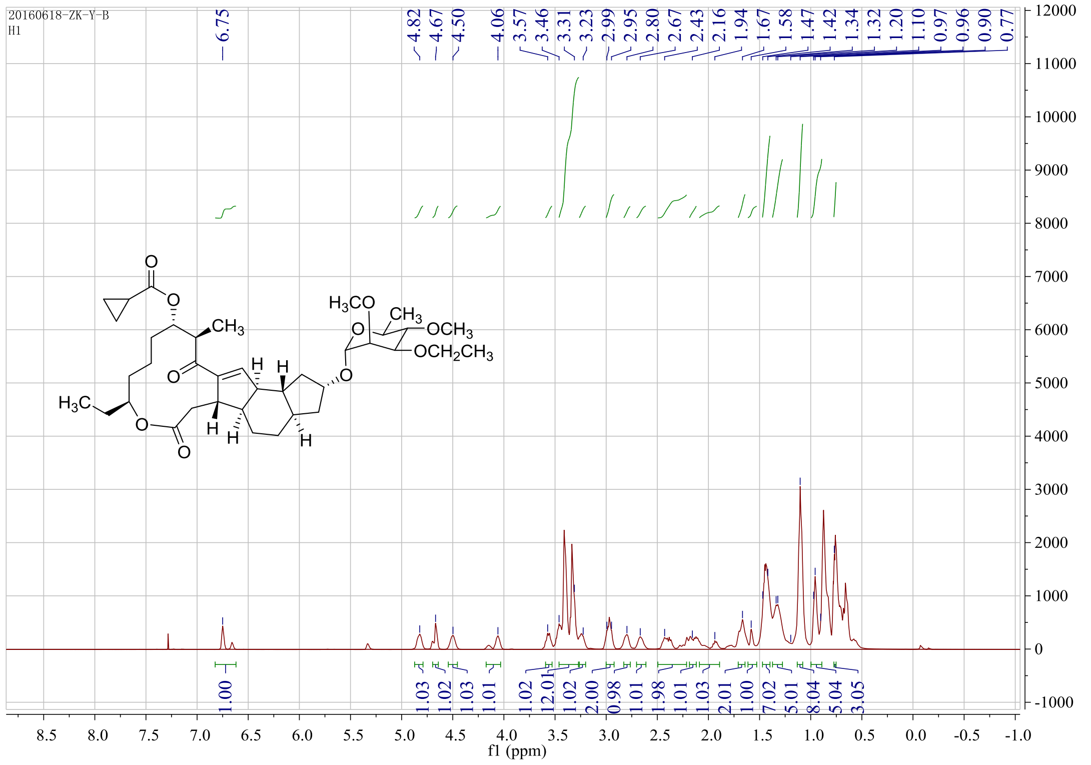

^{13}C NMR of **8a**

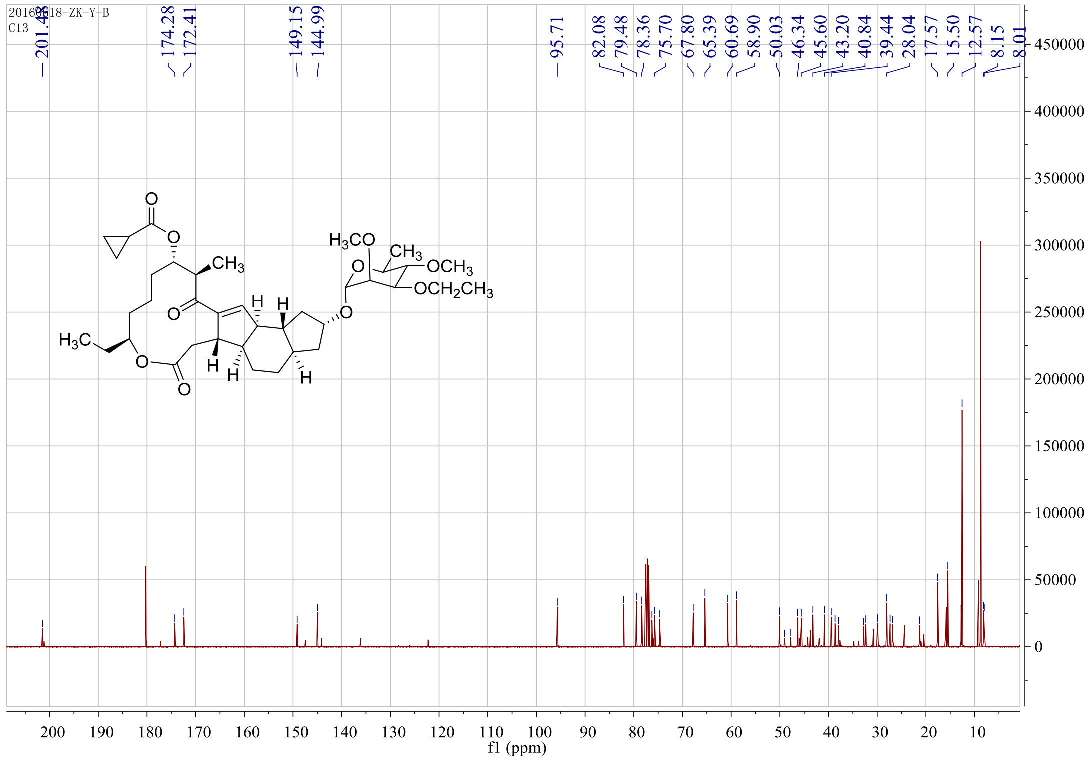

^{1}H NMR of **8b**

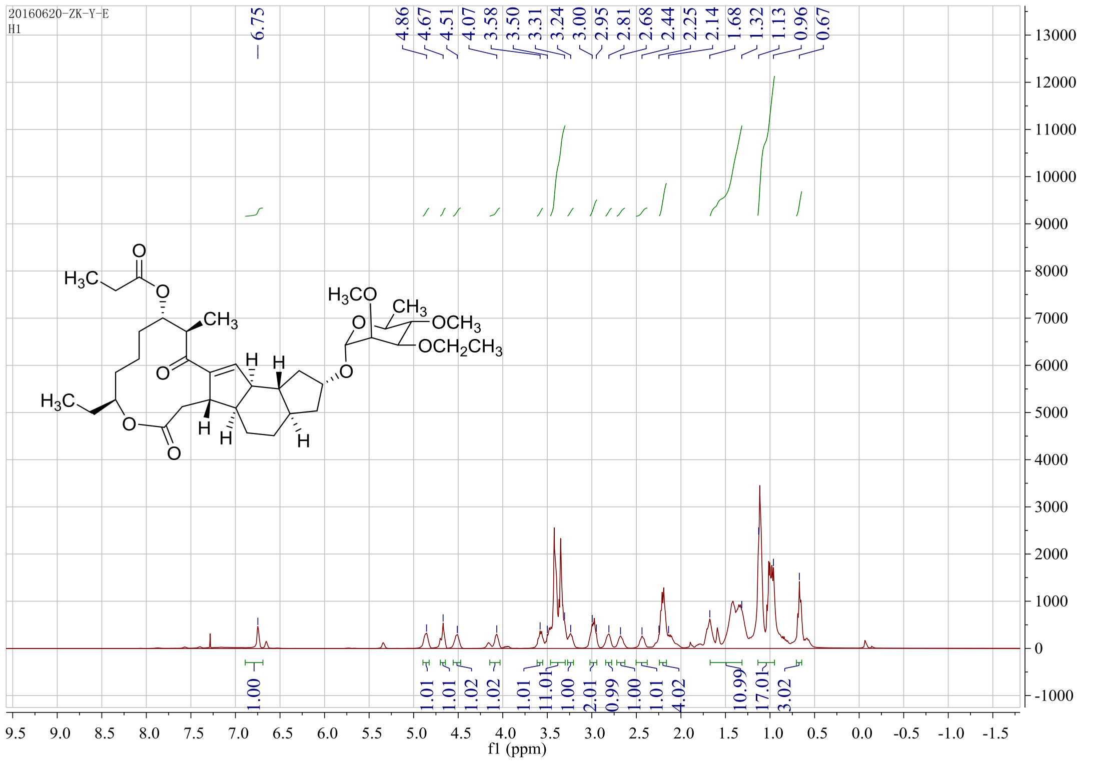

^{13}C NMR of **8b**

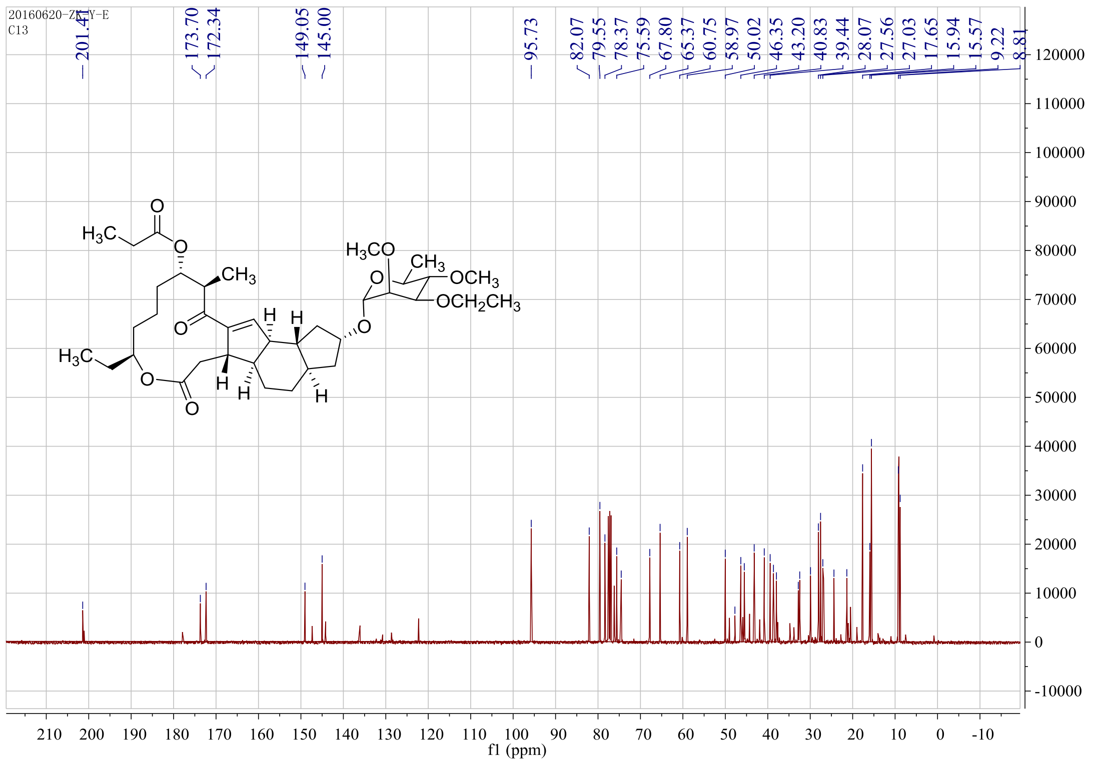

^{1}H NMR of **8c**

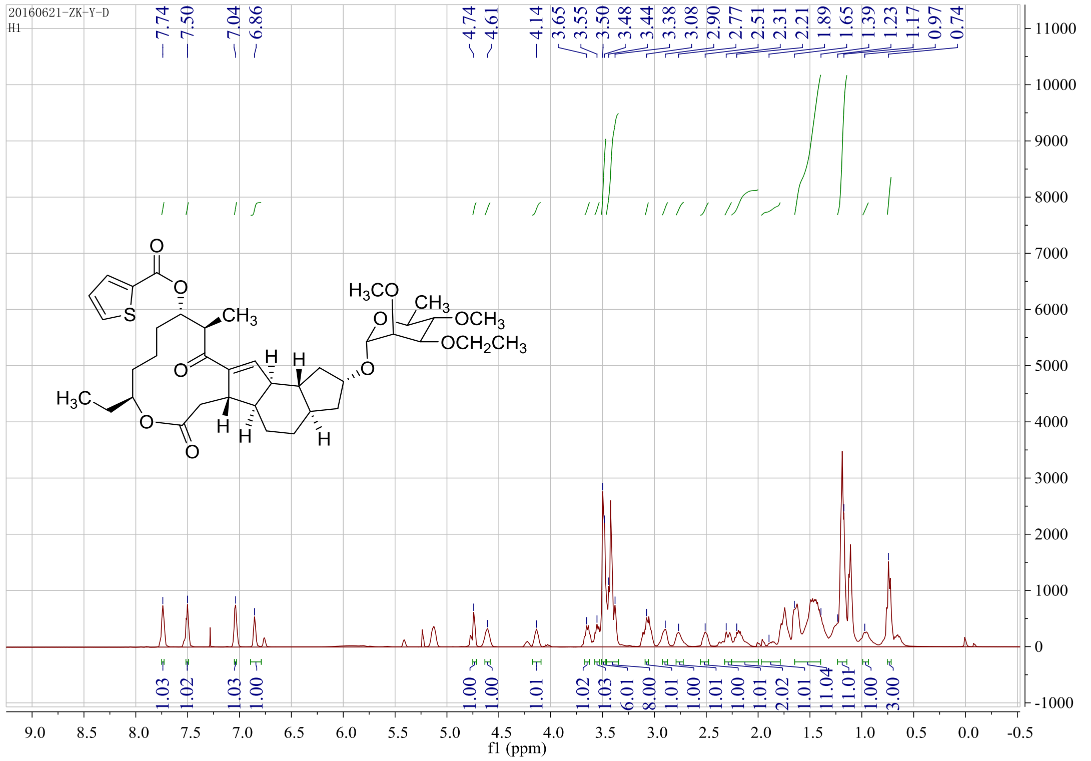

^{13}C NMR of **8c**

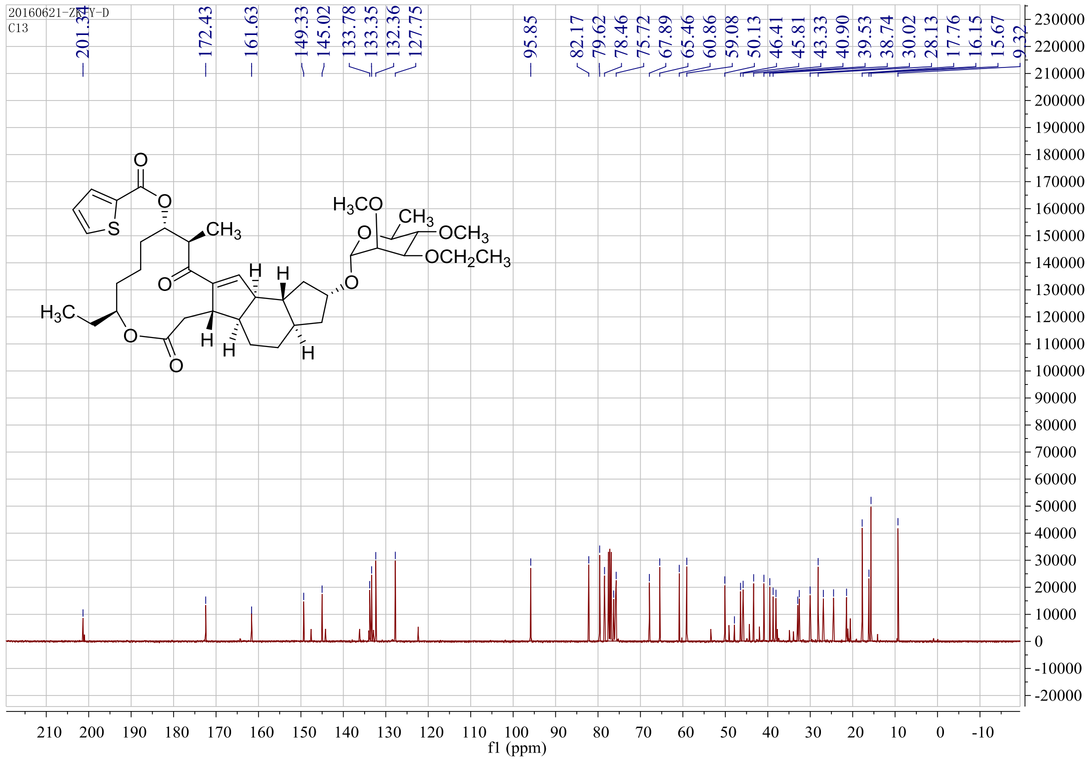

^{1}H NMR of **8d**

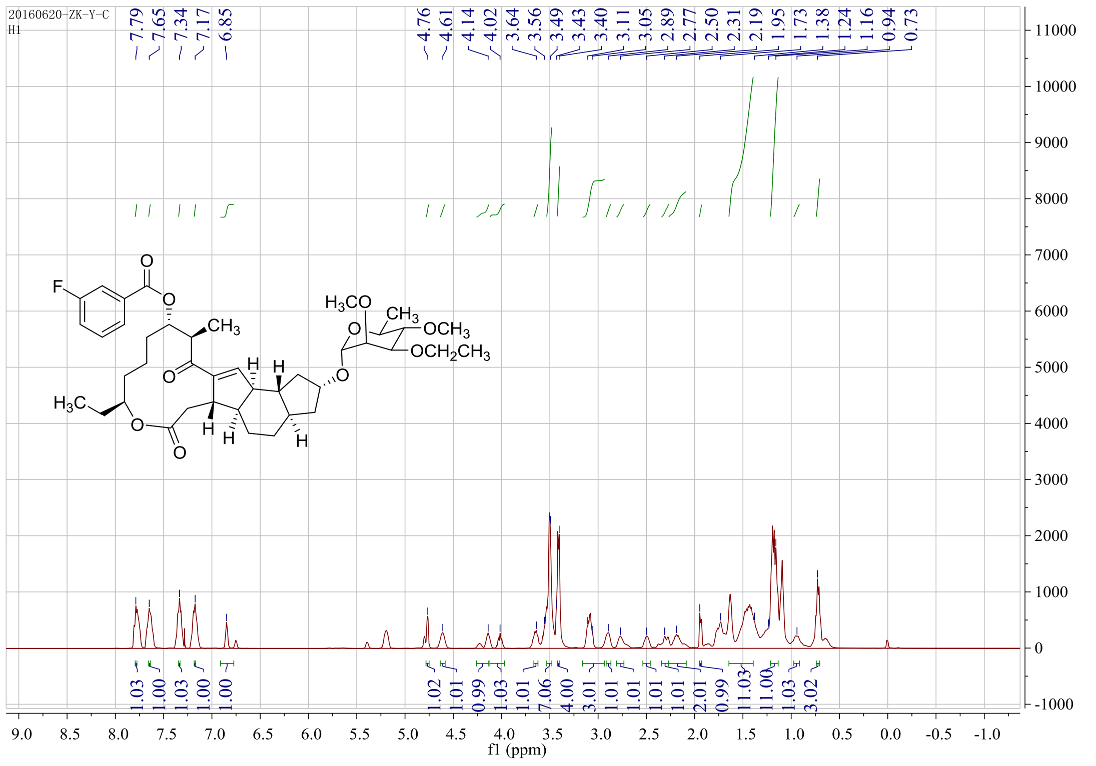

^{13}C NMR of **8d**

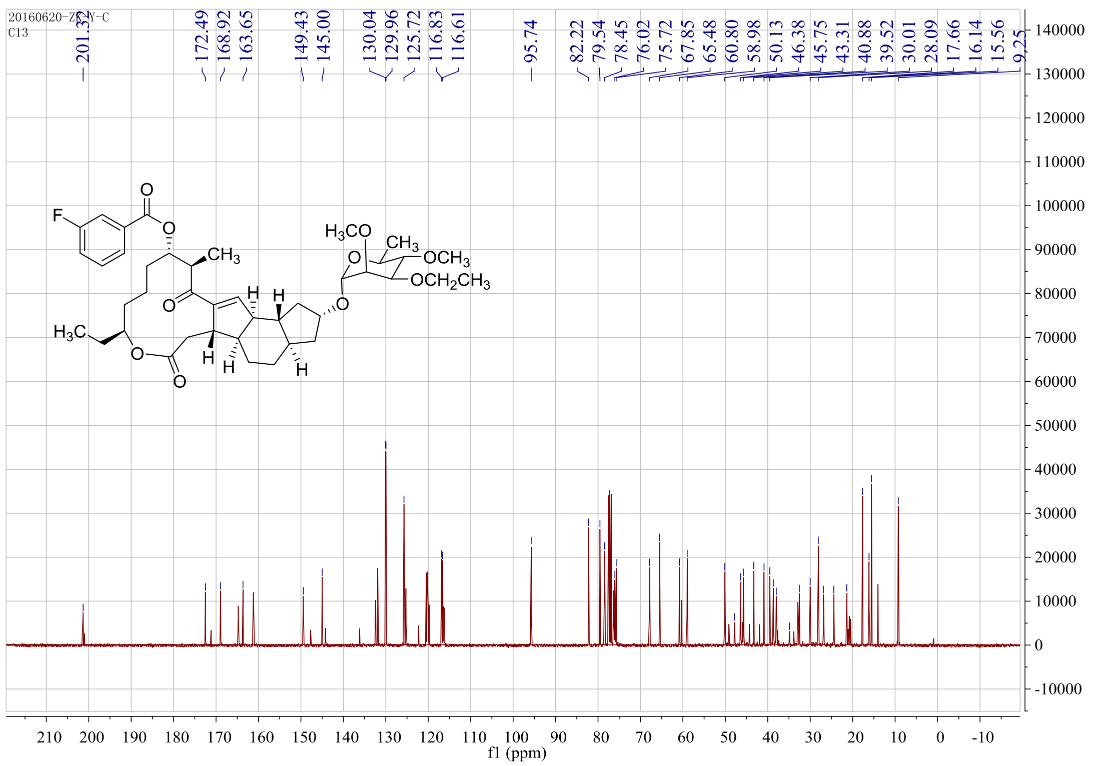

^{1}H NMR of **8e**

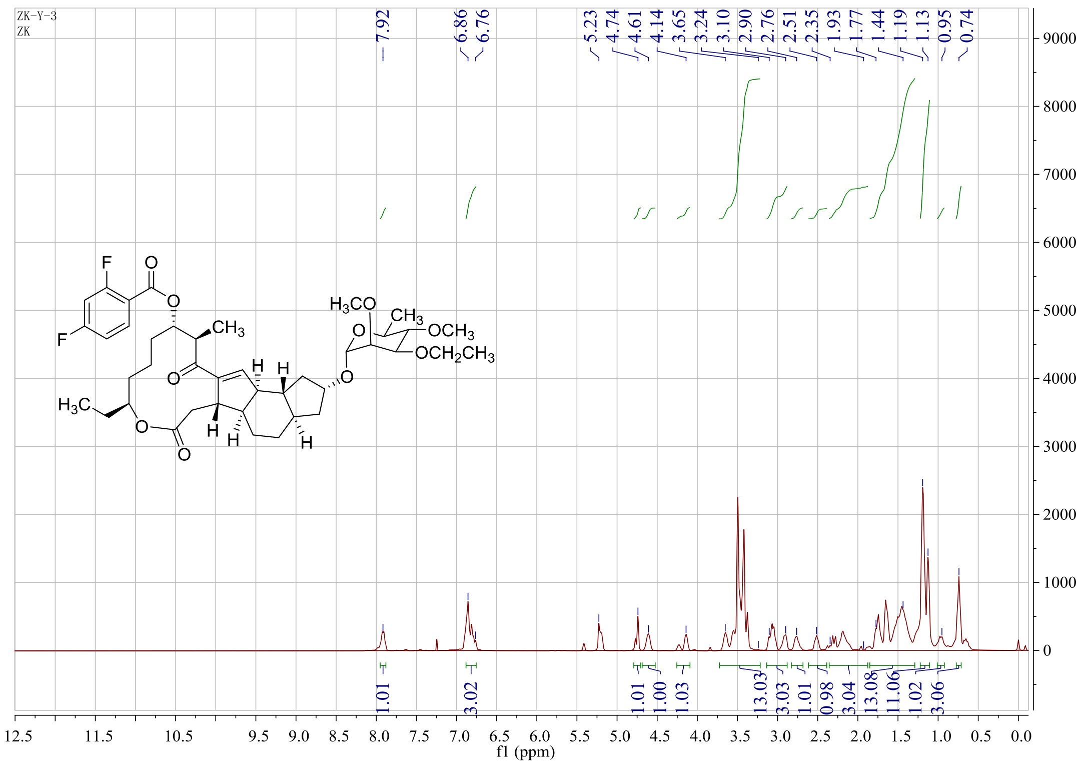

^{13}C NMR of **8e**

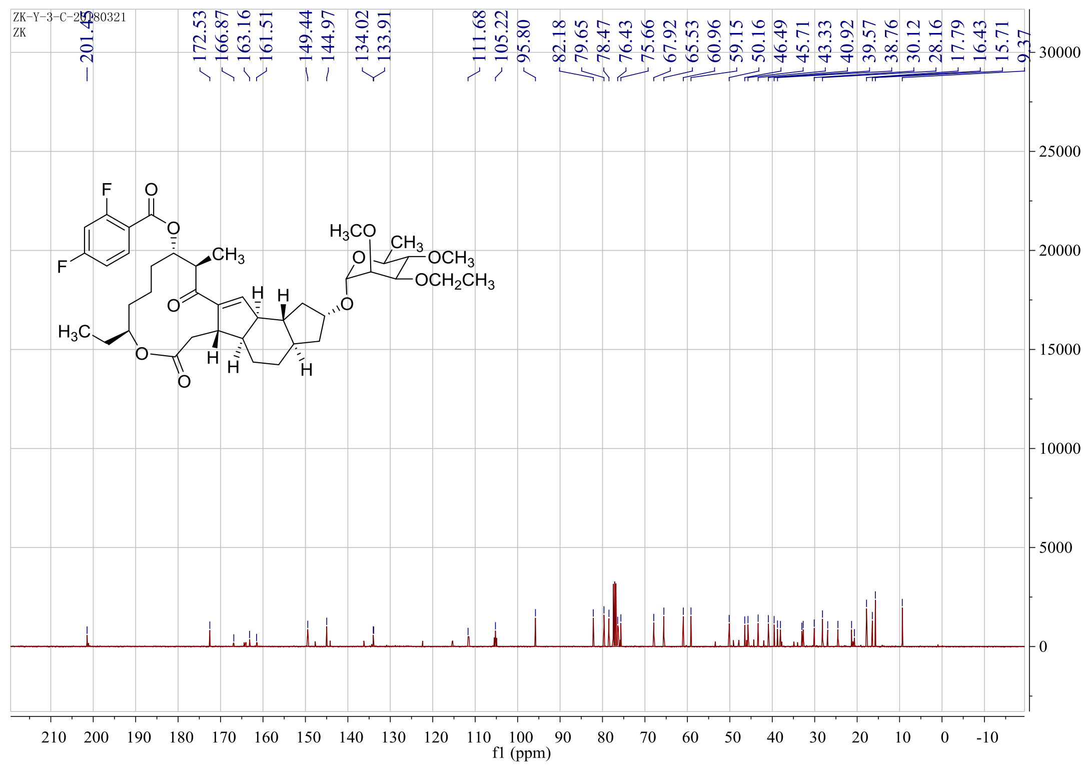

^{1}H NMR of **8f**

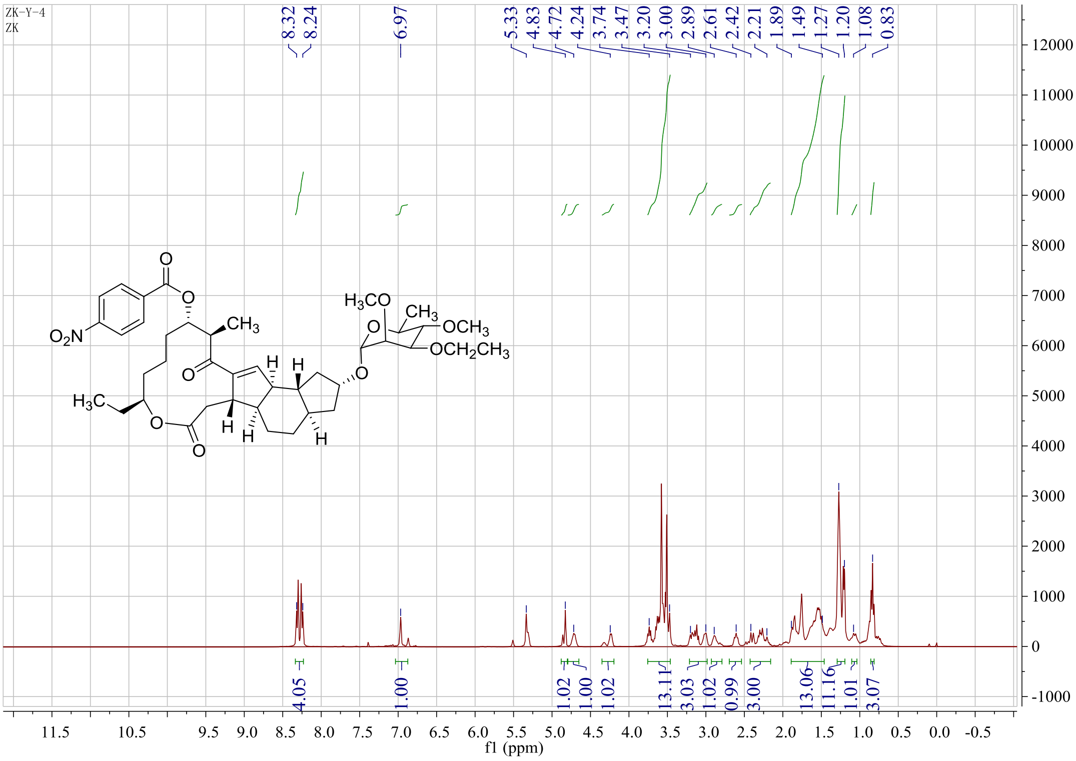

^{13}C NMR of **8f**

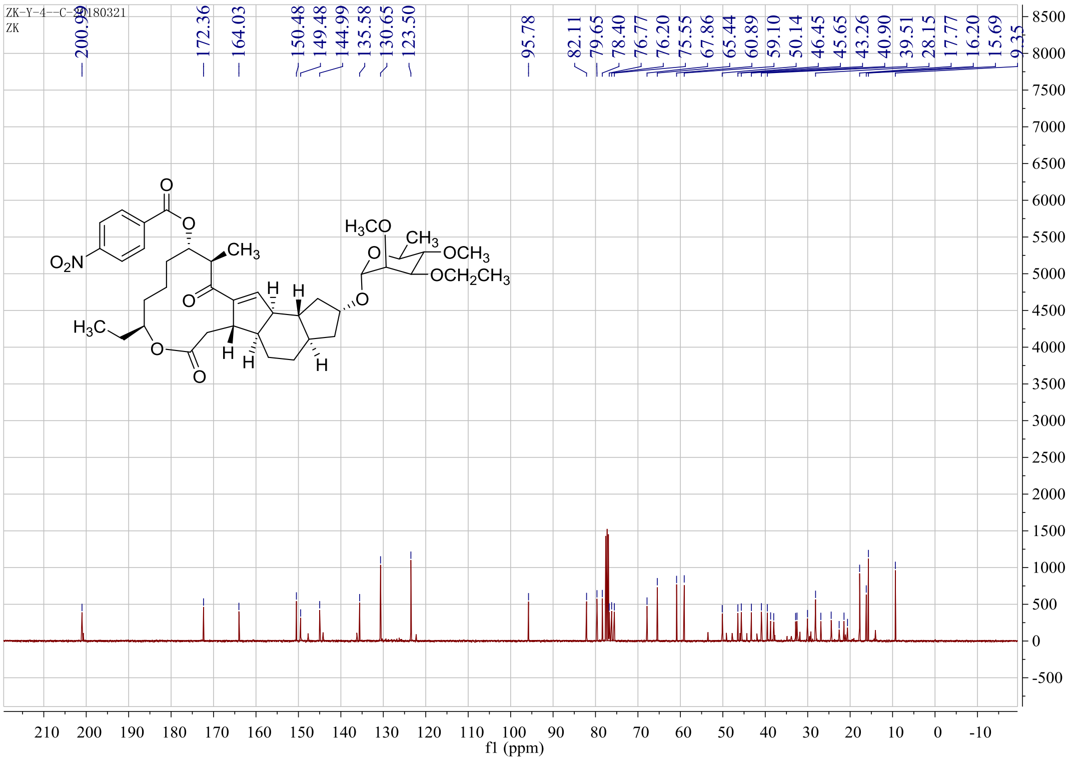

^{1}H NMR of **8g**

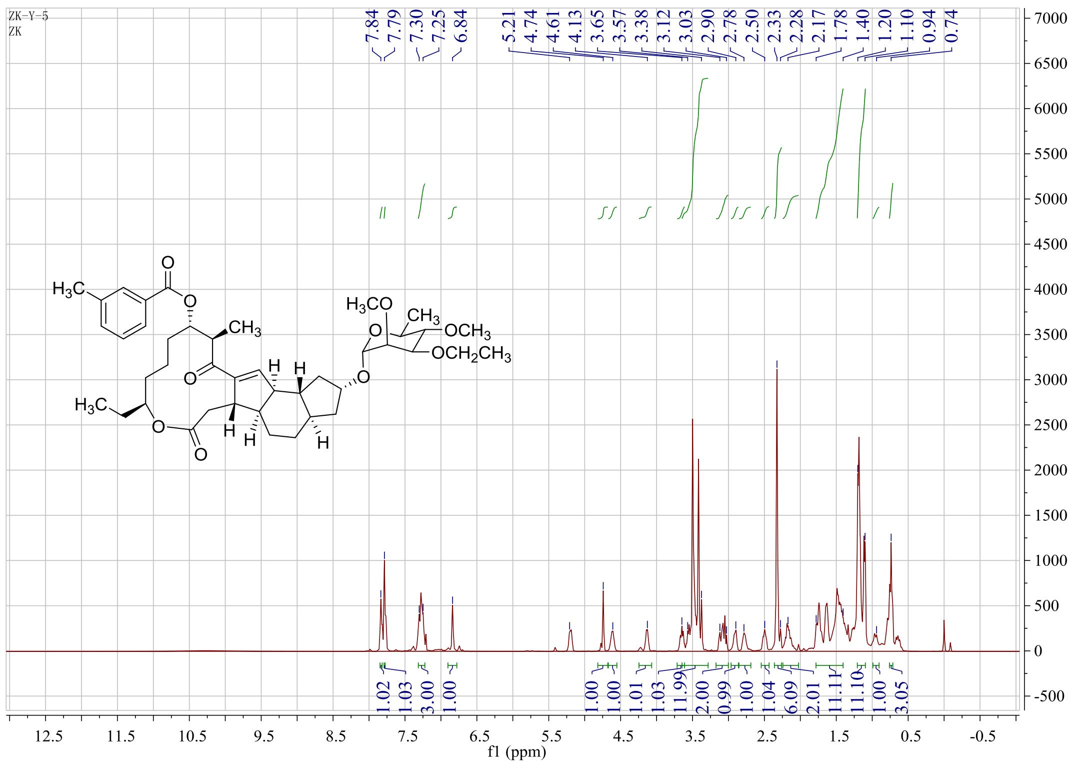

^{13}C NMR of **8g**

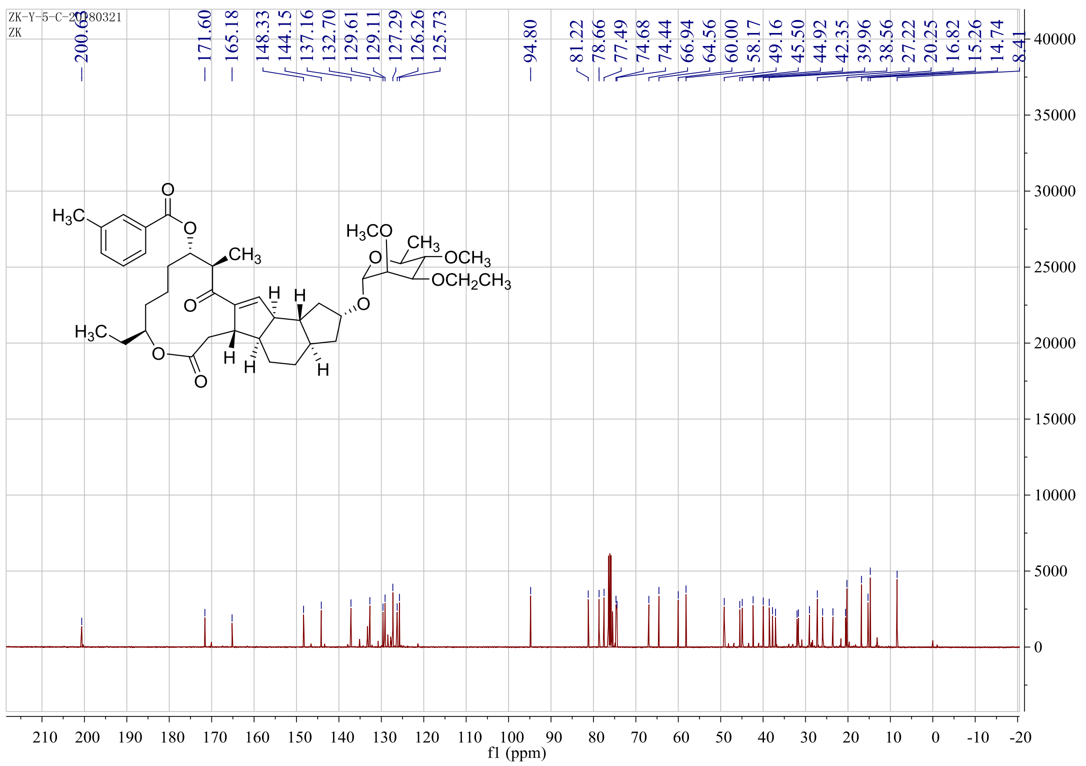

^{1}H NMR of **8h**

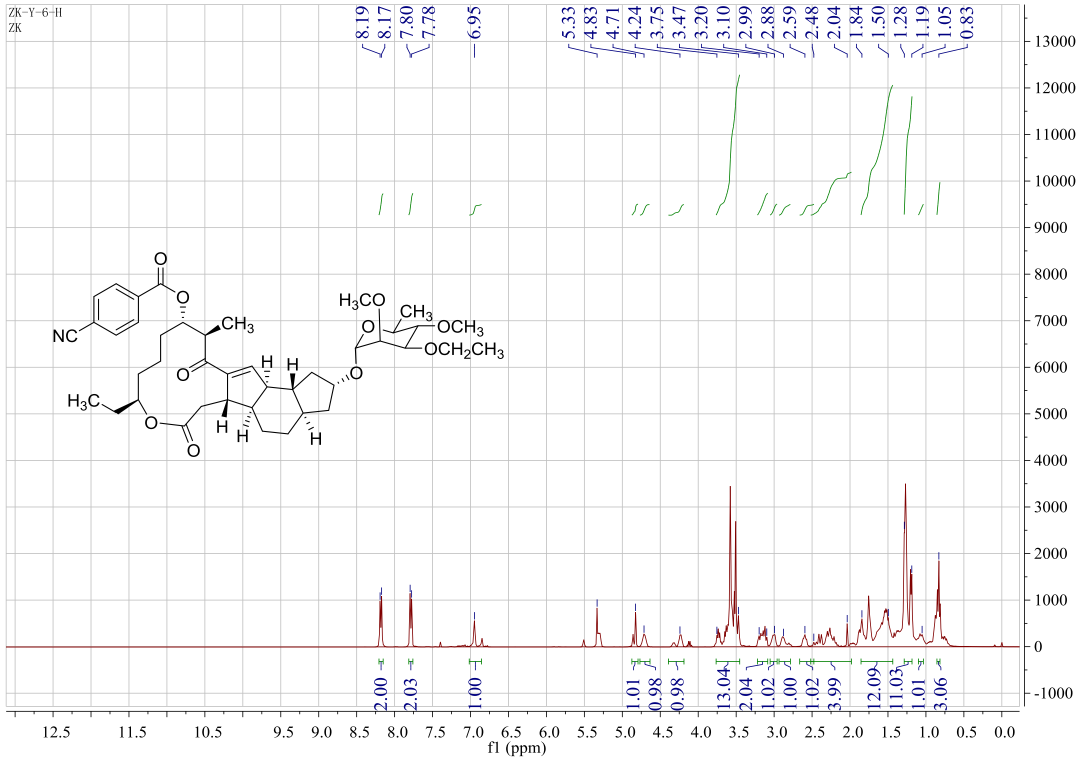

^{13}C NMR of **8h**

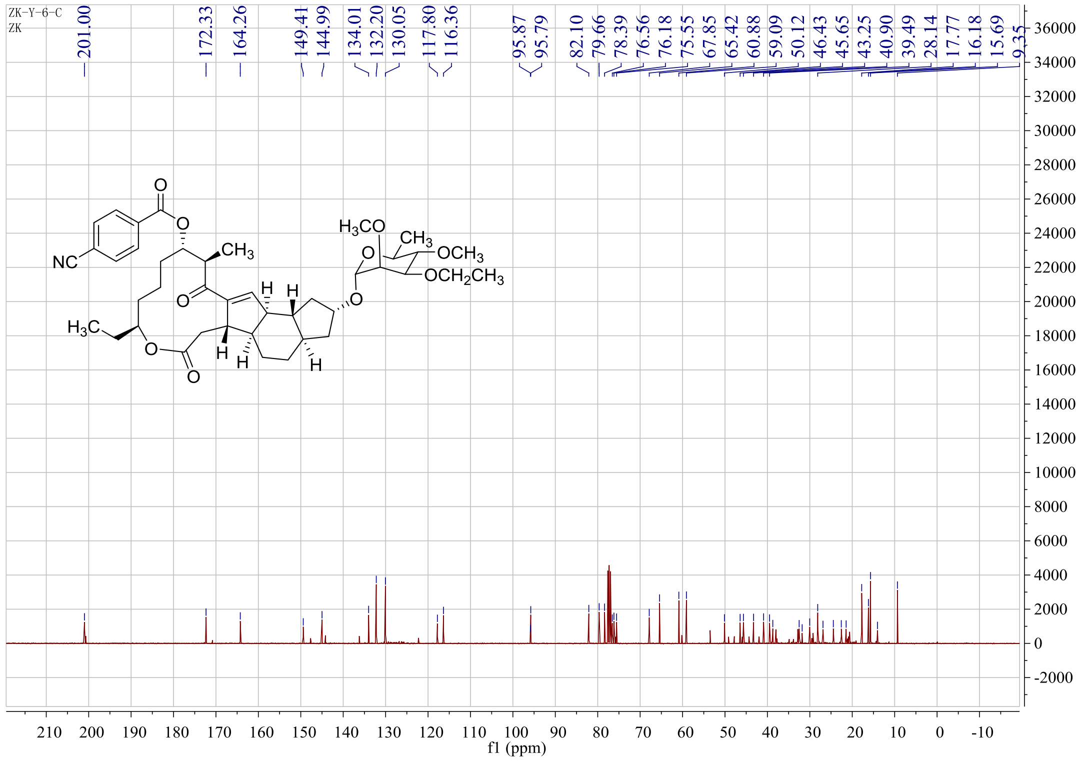

^{1}H NMR of **8i**

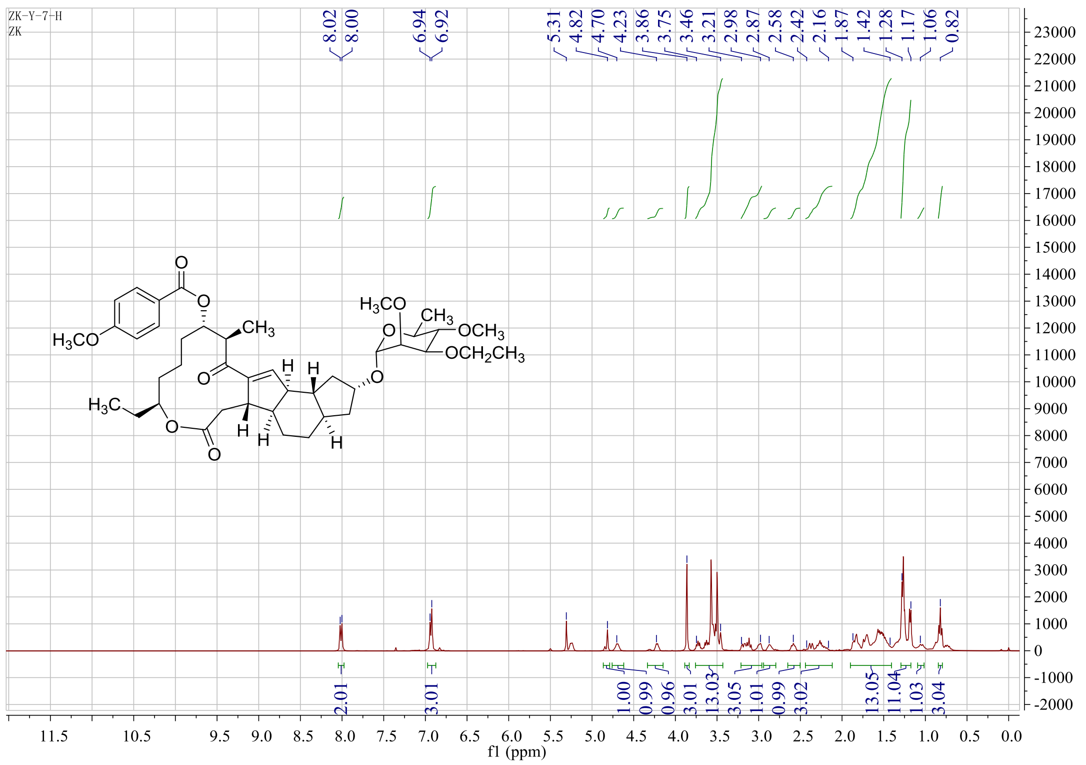

^{13}C NMR of **8i**

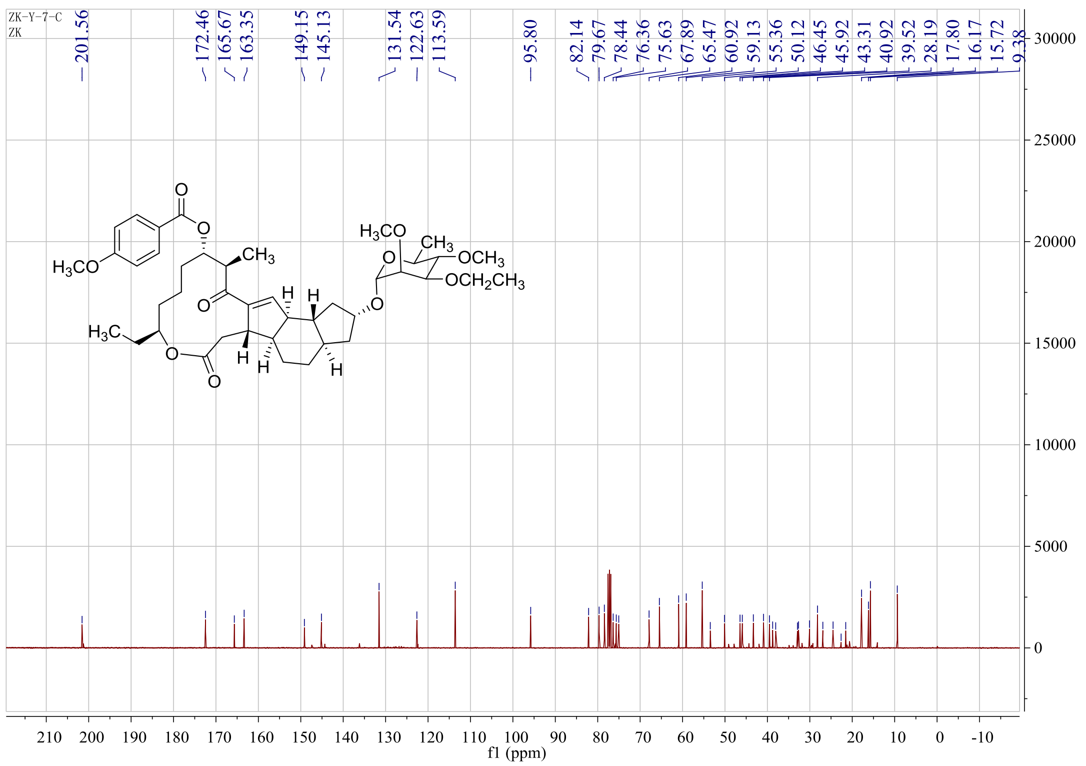

MS of **1**

MALDI,ZK-CC,20161223

Analysis Info

Analysis Name	D:\Data\MALDI\2016\1223\ZK-CC_0_E12_000001.d	Acquisition Date	12/23/2016 6:20:10 PM
Method	MALDI_P_100-3000	Operator	
Sample Name		Instrument	solariX
Comment			

Acquisition Parameter

Acquisition Mode	Single MS	Acquired Scans	5	Calibration Date	Fri Dec 23 05:39:24 2016
Polarity	Positive	No. of Cell Fills	1	Data Acquisition Size	2097152
Broadband Low Mass	202.1 m/z	No. of Laser Shots	10	Data Processing Size	4194304
Broadband High Mass	1300.0 m/z	Laser Power	28.4 lp	Apodization	Sine-Bell Multiplication
Source Accumulation	0.001 sec	Laser Shot Frequency	0.020 sec		
Ion Accumulation Time	1.000 sec				

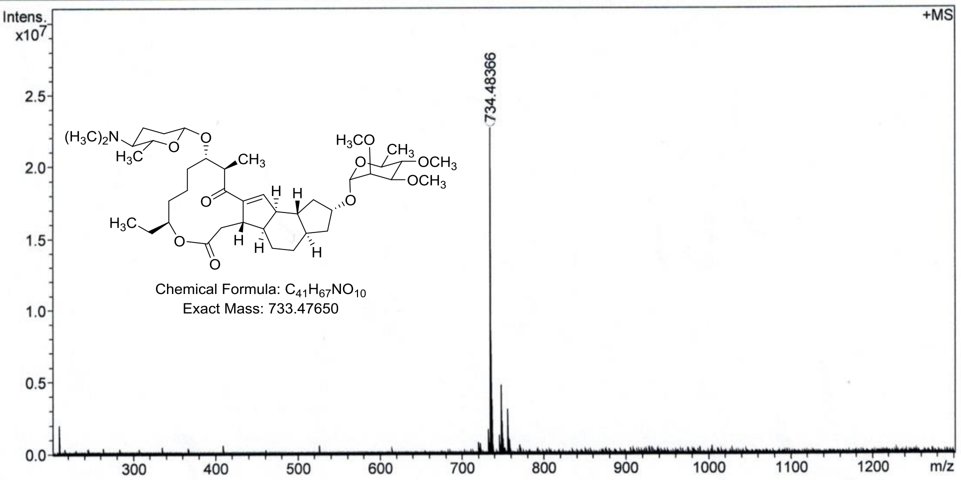

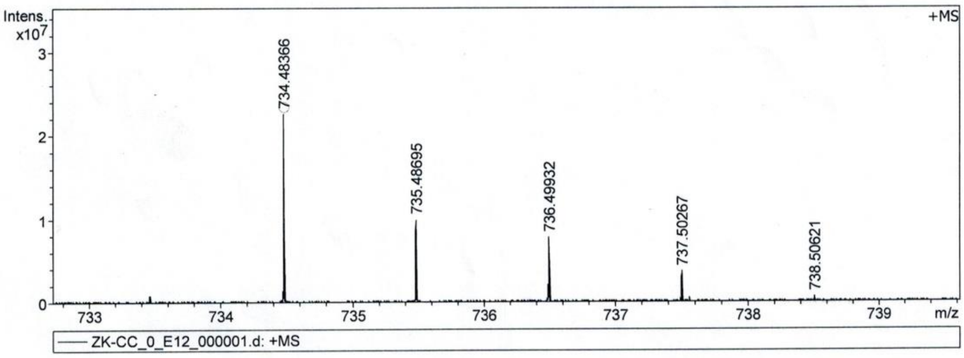

Meas. m/z	#	Ion Formula	Score	m/z	err [ppm]	Mean err [ppm]	mSigma	rdb	e⁻ Conf	N-Rule
734.483663	1	C41H68NO10	100.00	734.483774	-0.2	0.1	14.7	8.5	even	ok

MS of **2**

Acquisition Parameter

Acquisition Mode	Single MS	Acquired Scans	3	Calibration Date	Fri Dec 9 06:41:47 2016
Polarity	Positive	No. of Cell Fills	1	Data Acquisition Size	2097152
Broadband Low Mass	202.1 m/z	No. of Laser Shots	10	Data Processing Size	4194304
Broadband High Mass	700.0 m/z	Laser Power	34.6 lp	Apodization	Sine-Bell Multiplication
Source Accumulation	0.001 sec	Laser Shot Frequency	0.020 sec		
Ion Accumulation Time	0.300 sec				

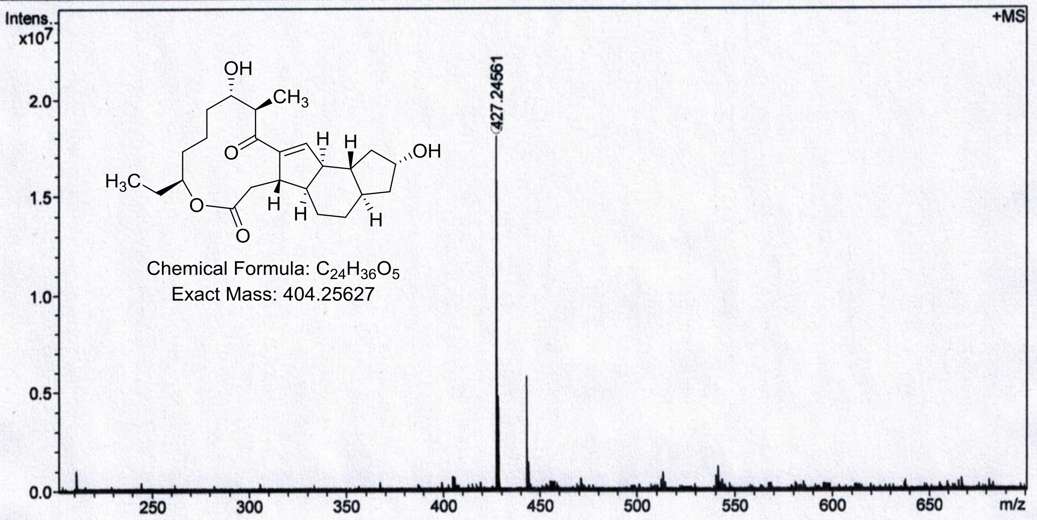

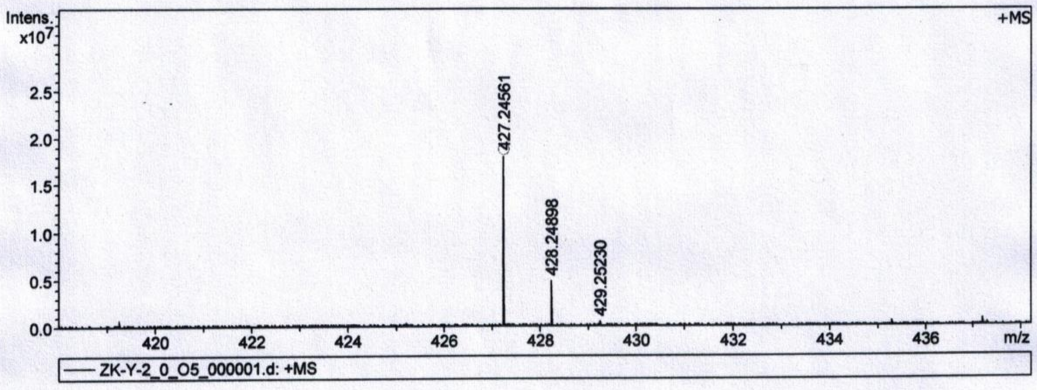

Meas. m/z	#	Ion Formula	Score	m/z	err [ppm]	Mean err [ppm]	mSigma	rdb	e⁻ Conf	N-Rule
427.245611	1	C24H36NaO5	100.00	427.245495	0.3	-0.3	7.7	6.5	even	ok

MS of **4**

Acquisition Parameter

Acquisition Mode	Single MS	Acquired Scans	4	Calibration Date	Fri Dec 9 06:41:47 2016
Polarity	Positive	No. of Cell Fills	1	Data Acquisition Size	2097152
Broadband Low Mass	202.1 m/z	No. of Laser Shots	10	Data Processing Size	4194304
Broadband High Mass	1500.0 m/z	Laser Power	34.6 lp	Apodization	Sine-Bell Multiplication
Source Accumulation	0.001 sec	Laser Shot Frequency	0.020 sec		
Ion Accumulation Time	0.300 sec				

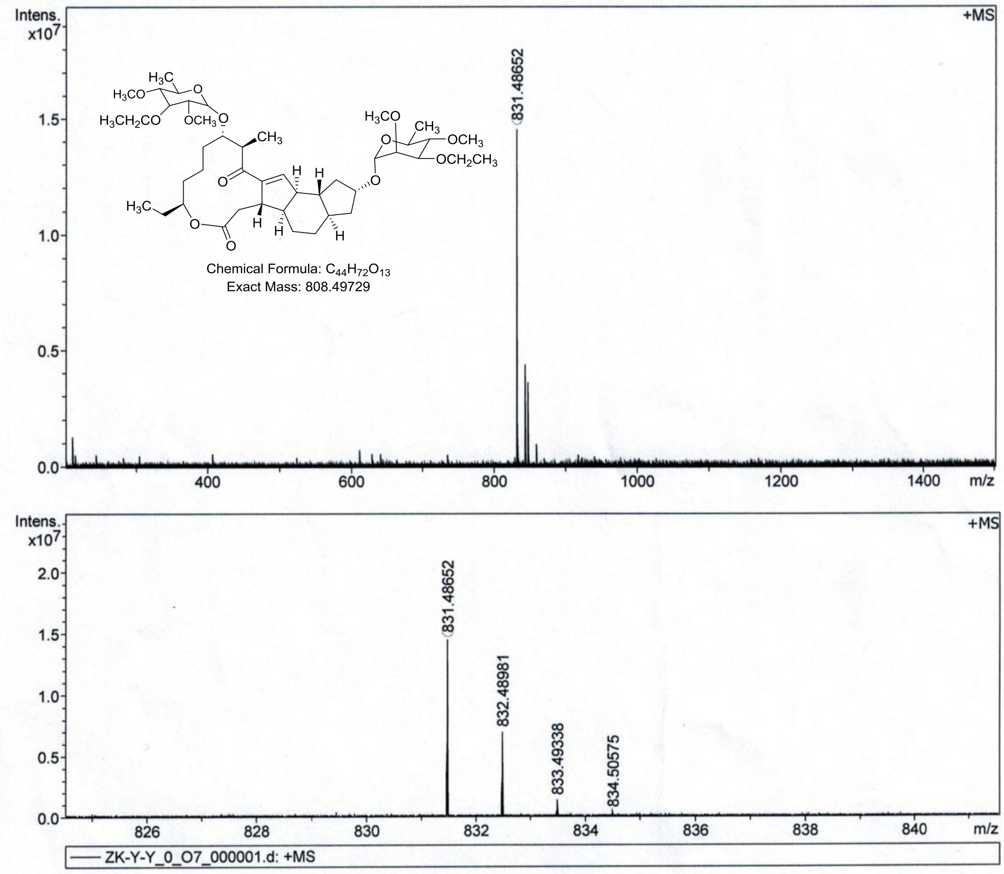

Meas. m/z	#	Ion Formula	Score	m/z	err [ppm]	Mean err [ppm]	mSigma	rdb	e⁻ Conf	N-Rule
831.486515	1	C44H72NaO13	100.00	831.486513	0.0	-0.0	23.2	8.5	even	ok

MS of **5**

Acquisition Parameter

Acquisition Mode	Single MS	Acquired Scans	11	Calibration Date	Tue Jan 3 05:07:39 2017
Polarity	Positive	No. of Cell Fills	1	Data Acquisition Size	2097152
Broadband Low Mass	202.1 m/z	No. of Laser Shots	10	Data Processing Size	4194304
Broadband High Mass	1000.0 m/z	Laser Power	35.2 lp	Apodization	Sine-Bell Multiplication
Source Accumulation	0.001 sec	Laser Shot Frequency	0.020 sec		
Ion Accumulation Time	0.300 sec				

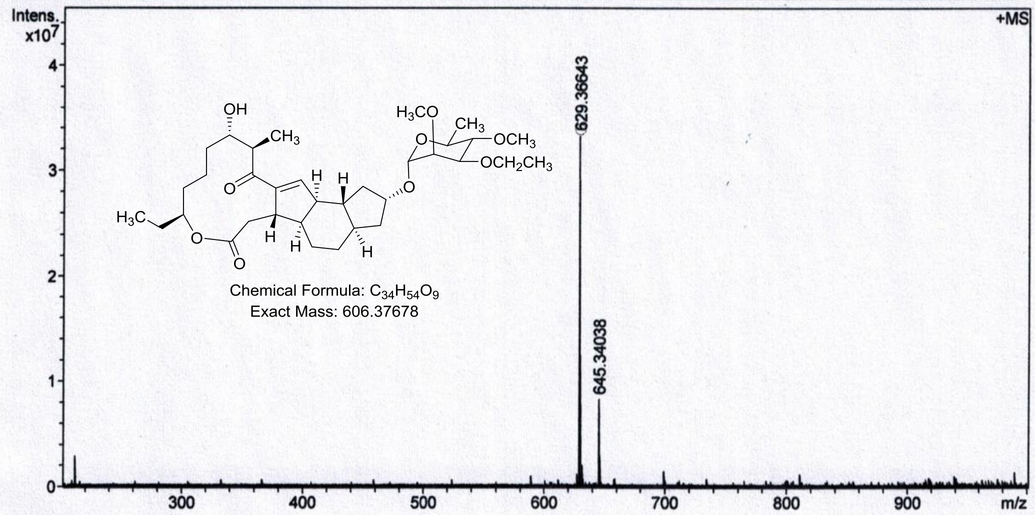

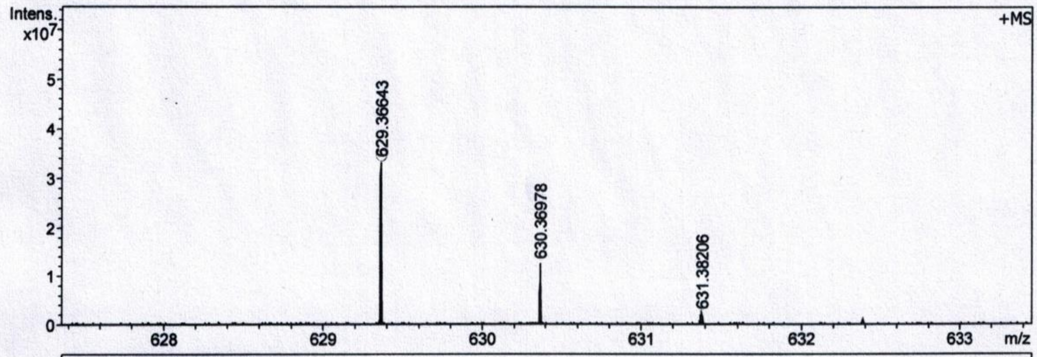

Meas. m/z	#	Ion Formula	Score	m/z	err [ppm]	Mean err [ppm]	mSigma	rdb	e⁻ Conf	N-Rule
629.366428	1	C34H54NaO9	100.00	629.366004	-0.7	-0.7	15.0	7.5	even	ok

MS of **spinetoram J**

MALDI,ZK-Y,20171010

Analysis Info

Analysis Name	D:\Data\MALDI\2017\1011\ZK-Y_0_N16_000002.d	Acquisition Date	10/11/2017 5:24:05 PM
Method	MALDI_P_100-3000	Operator	
Sample Name	MURU-N-ESI	Instrument	solariX
Comment			

Acquisition Parameter

Acquisition Mode	Single MS	Acquired Scans	17	Calibration Date	Wed Oct 11 05:04:55
Polarity	Positive	No. of Cell Fills	1	Data Acquisition Size	2097152
Broadband Low Mass	101.1 m/z	No. of Laser Shots	10	Data Processing Size	4194304
Broadband High Mass	1450.0 m/z	Laser Power	28.8 lp	Apodization	Sine-Bell Multiplication
Source Accumulation	0.001 sec	Laser Shot Frequency	0.020 sec		
Ion Accumulation Time	0.300 sec				

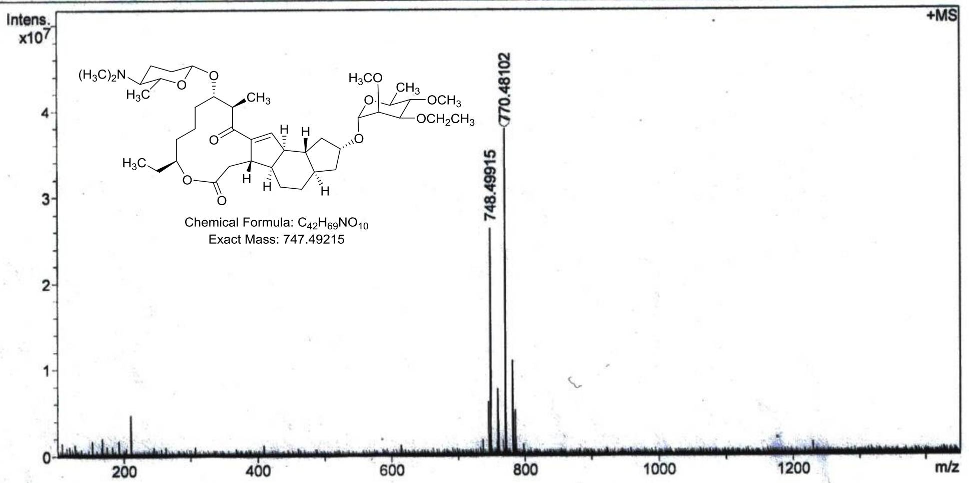

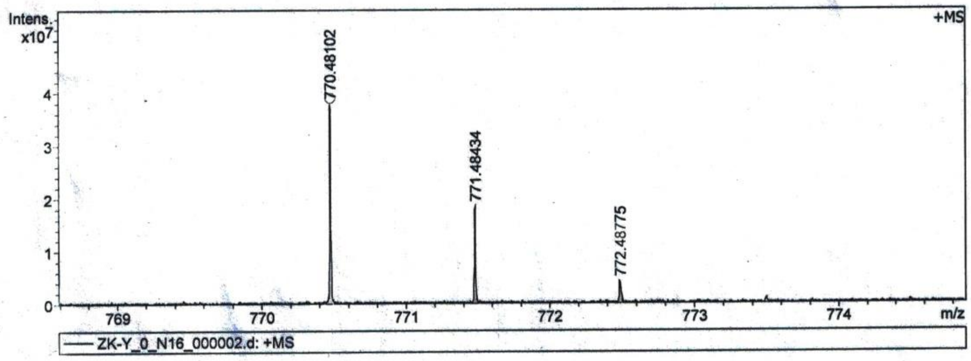

Meas. m/z	#	Ion Formula	Score	m/z	err [ppm]	Mean err [ppm]	mSigma	rdb	e⁻ Conf	N-Rule
770.481016	1	C42H69NNaO10	100.00	770.481368	-0.5	0.4	16.2	8.5	even	ok

MS of **7a**

Acquisition Parameter

Acquisition Mode	Single MS	Acquired Scans	2	Calibration Date	Fri Jun 3 05:53:25 2016
Polarity	Positive	No. of Cell Fills	1	Data Acquisition Size	2097152
Broadband Low Mass	202.1 m/z	No. of Laser Shots	10	Data Processing Size	4194304
Broadband High Mass	1300.0 m/z	Laser Power	28.2 lp	Apodization	Sine-Bell Multiplication
Source Accumulation	0.001 sec	Laser Shot Frequency	0.020 sec		
Ion Accumulation Time	0.800 sec				

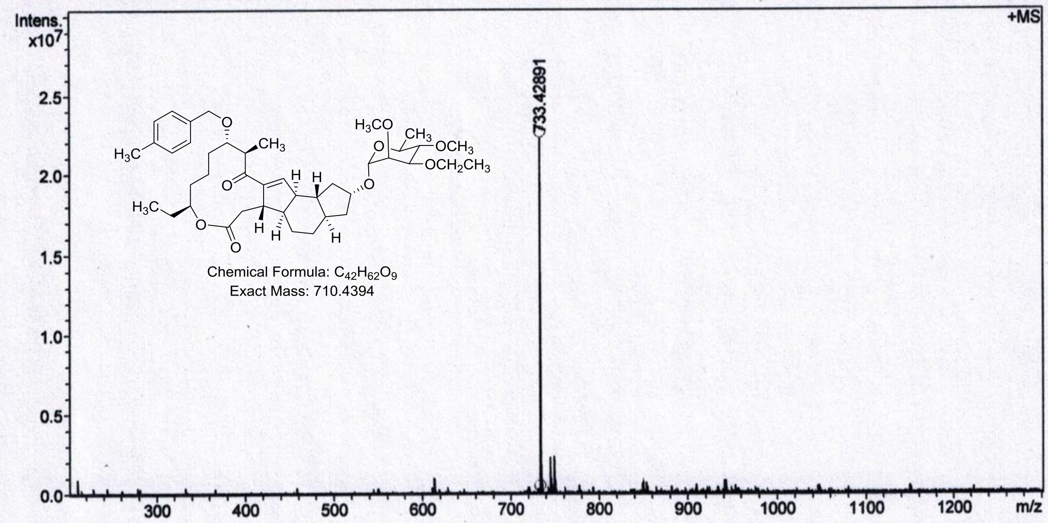

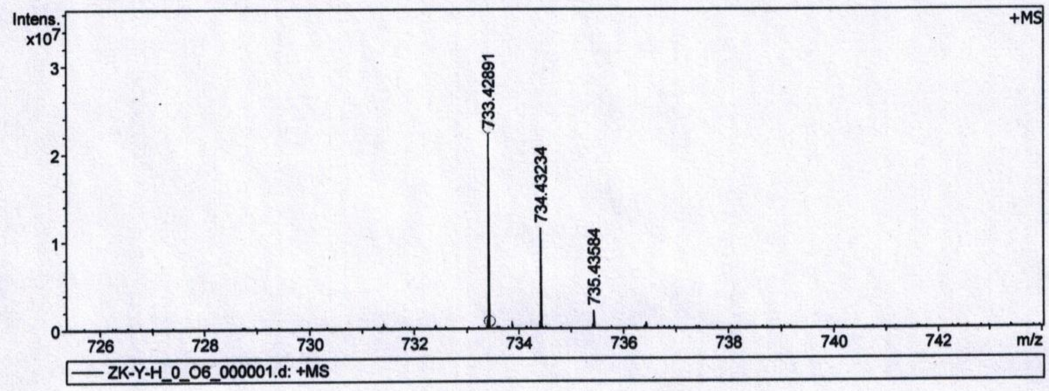

Meas. m/z	#	Ion Formula	Score	m/z	err [ppm]	Mean err [ppm]	mSigma	rdb	e⁻ Conf	N-Rule
733.428914	1	C42H62NaO9	100.00	733.428604	0.4	-0.5	27.9	11.5	even	ok
733.444150	1	C31H65N4O15	n.a.	733.444094	-0.1		n.a.	1.5	even	ok
	2	C30H59N11O10	n.a.	733.444089	-0.1		n.a.	7.0	odd	ok
	3	C32H61N8O11	n.a.	733.445431	1.7		n.a.	6.5	even	ok
	4	C33H67NO16	n.a.	733.445436	1.8		n.a.	1.0	odd	ok
	5	C29H63N7O14	n.a.	733.442751	-1.9		n.a.	2.0	odd	ok

MS of **7b**

Acquisition Parameter

Acquisition Mode	Single MS	Acquired Scans	3	Calibration Date	Fri Jun 3 05:53:25 2016
Polarity	Positive	No. of Cell Fills	1	Data Acquisition Size	2097152
Broadband Low Mass	202.1 m/z	No. of Laser Shots	10	Data Processing Size	4194304
Broadband High Mass	1300.0 m/z	Laser Power	28.2 lp	Apodization	Sine-Bell Multiplication
Source Accumulation	0.001 sec	Laser Shot Frequency	0.020 sec		
Ion Accumulation Time	0.800 sec				

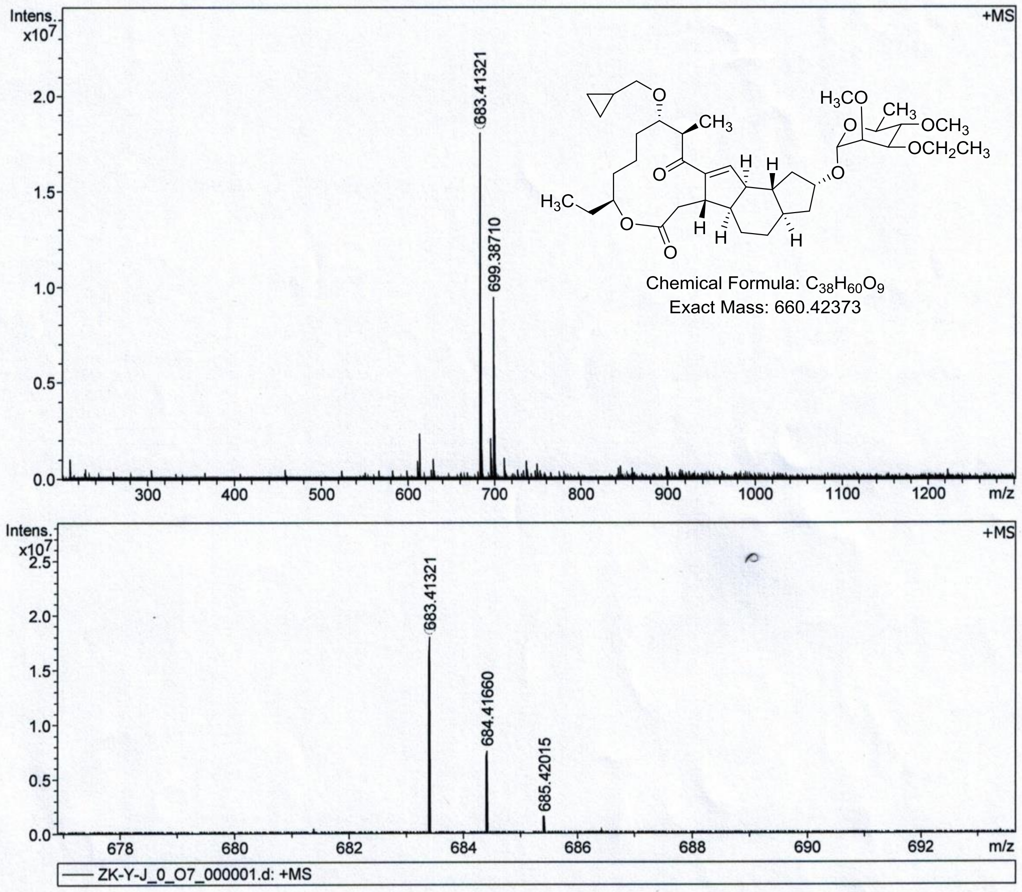

Meas. m/z	#	Ion Formula	Score	m/z	err [ppm]	Mean err [ppm]	mSigma	rdb	e⁻ Conf	N-Rule
683.413206	1	C38H60NaO9	100.00	683.412954	-0.4	-0.4	13.9	8.5	even	ok

MS of **7c**

Acquisition Parameter

Acquisition Mode	Single MS	Acquired Scans	2	Calibration Date	Thu Jun 23 05:30:01
Polarity	Positive	No. of Cell Fills	1	Data Acquisition Size	2096152
Broadband Low Mass	202.1 m/z	No. of Laser Shots	10	Data Processing Size	4194304
Broadband High Mass	1300.0 m/z	Laser Power	35.8 lp	Apodization	Sine-Bell Multiplication
Source Accumulation	0.001 sec	Laser Shot Frequency	0.020 sec		
Ion Accumulation Time	0.800 sec				

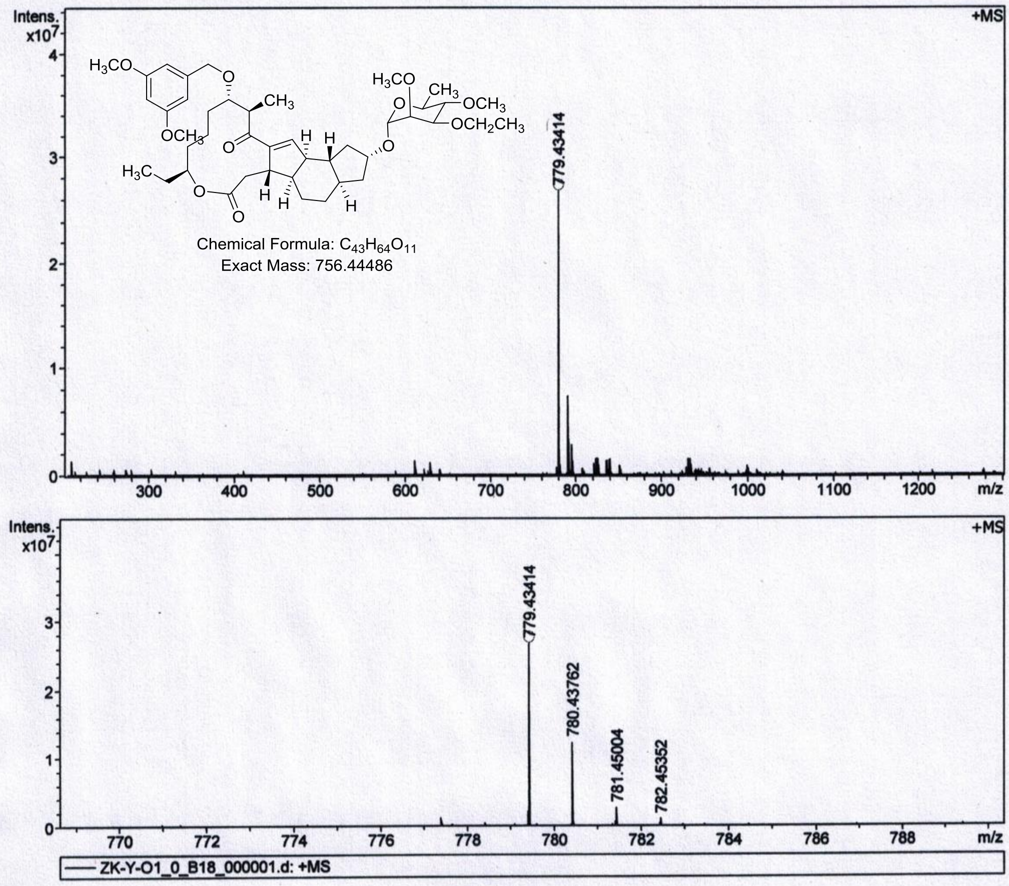

Meas. m/z	#	Ion Formula	Score	m/z	err [ppm]	Mean err [ppm]	mSigma	rdb	e⁻ Conf	N-Rule
779.434141	1	C43H64NaO11	100.00	779.434084	-0.1	-0.2	19.9	11.5	even	ok

MS of **7d**

Acquisition Parameter

Acquisition Mode	Single MS	Acquired Scans	3	Calibration Date	Thu Jun 23 05:30:01
Polarity	Positive	No. of Cell Fills	1	Data Acquisition Size	2096152
Broadband Low Mass	202.1 m/z	No. of Laser Shots	10	Data Processing Size	4194304
Broadband High Mass	1300.0 m/z	Laser Power	31.4 lp	Apodization	Sine-Bell Multiplication
Source Accumulation	0.001 sec	Laser Shot Frequency	0.020 sec		
Ion Accumulation Time	0.800 sec				

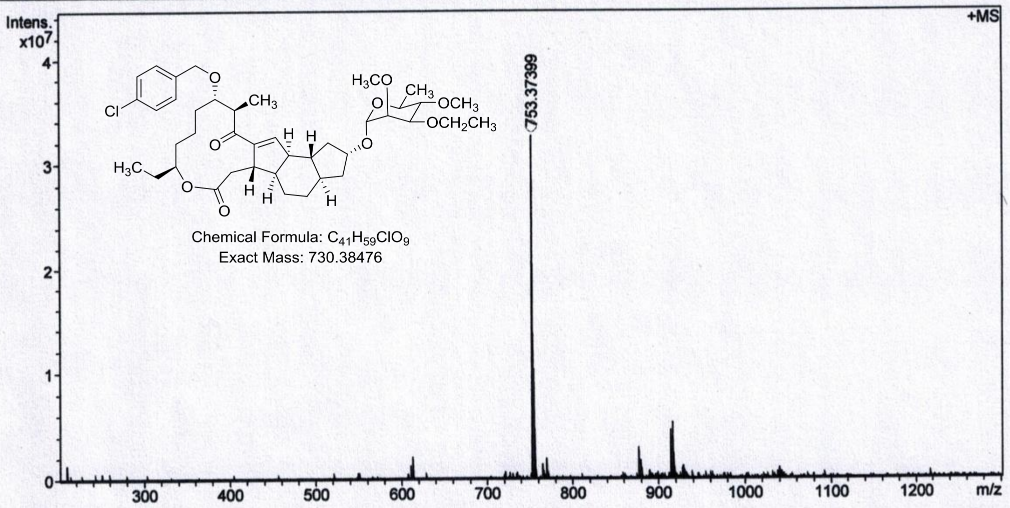

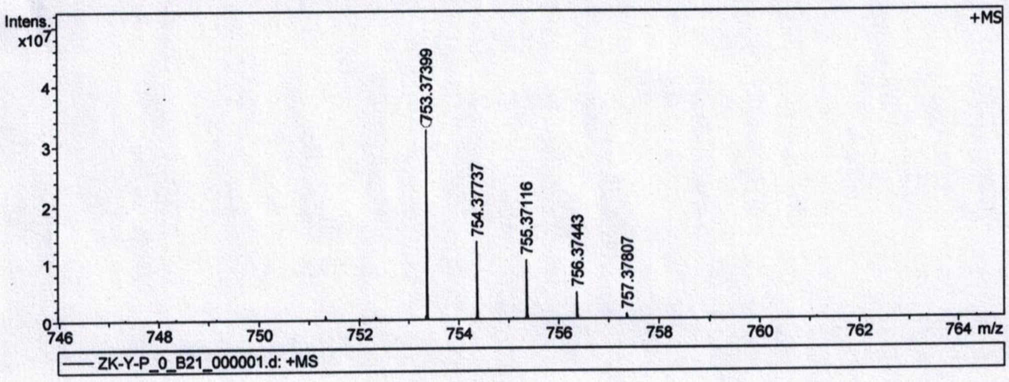

Meas. m/z	#	Ion Formula	Score	m/z	err [ppm]	Mean err [ppm]	mSigma	rdb	e⁻ Conf	N-Rule
753.373992	1	C41H59ClNaO9	100.00	753.373982	-0.0	0.7	53.4	11.5	even	ok

MS of **7e**

Acquisition Parameter

Acquisition Mode	Single MS	Acquired Scans	5	Calibration Date	Thu Jun 23 05:30:01
Polarity	Positive	No. of Cell Fills	1	Data Acquisition Size	2097152
Broadband Low Mass	202.1 m/z	No. of Laser Shots	10	Data Processing Size	4194304
Broadband High Mass	1300.0 m/z	Laser Power	31.4 lp	Apodization	Sine-Bell Multiplication
Source Accumulation	0.001 sec	Laser Shot Frequency	0.020 sec		
Ion Accumulation Time	0.800 sec				

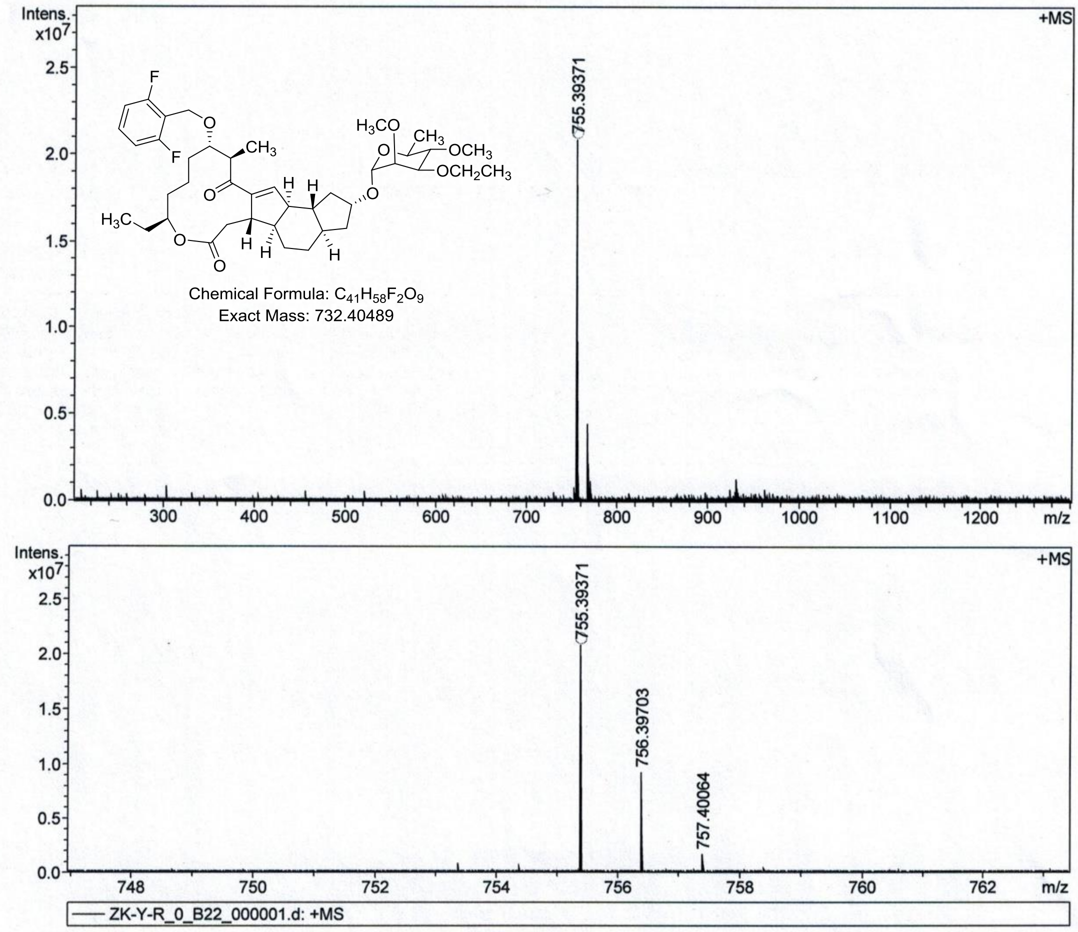

Meas. m/z	#	Ion Formula	Score	m/z	err [ppm]	Mean err [ppm]	mSigma	rdb	e⁻ Conf	N-Rule
755.393714	1	C41H58F2NaO9	100.00	755.394111	-0.5	0.5	18.3	11.5	even	ok

MS of **7f**

Acquisition Parameter

Acquisition Mode	Single MS	Acquired Scans	5	Calibration Date	Thu Jun 23 05:30:01
Polarity	Positive	No. of Cell Fills	1	Data Acquisition Size	2097152
Broadband Low Mass	202.1 m/z	No. of Laser Shots	10	Data Processing Size	4194304
Broadband High Mass	1300.0 m/z	Laser Power	37.0 lp	Apodization	Sine-Bell Multiplication
Source Accumulation	0.001 sec	Laser Shot Frequency	0.020 sec		
Ion Accumulation Time	0.800 sec				

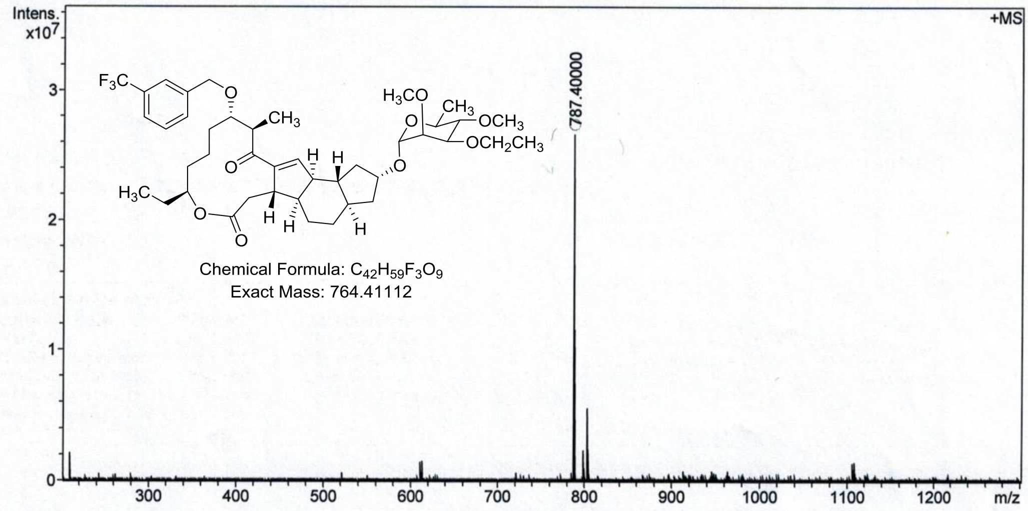

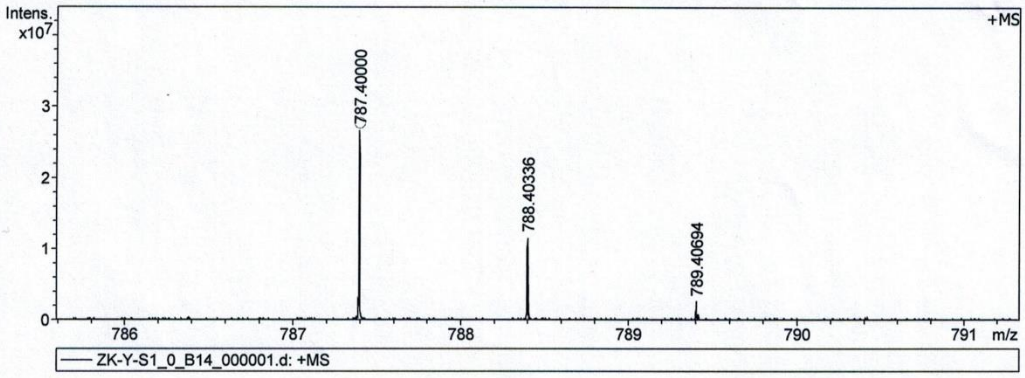

Meas. m/z	#	Ion Formula	Score	m/z	err [ppm]	Mean err [ppm]	mSigma	rdb	e⁻ Conf	N-Rule
787.400000	1	C42H59F3NaO9	100.00	787.400339	-0.4	0.4	17.9	11.5	even	ok

MS of **7g**

Acquisition Parameter

Acquisition Mode	Single MS	Acquired Scans	5	Calibration Date	Fri Jun 3 05:53:25 2016
Polarity	Positive	No. of Cell Fills	1	Data Acquisition Size	2097152
Broadband Low Mass	202.1 m/z	No. of Laser Shots	10	Data Processing Size	4194304
Broadband High Mass	1300.0 m/z	Laser Power	28.2 lp	Apodization	Sine-Bell Multiplication
Source Accumulation	0.001 sec	Laser Shot Frequency	0.020 sec		
Ion Accumulation Time	0.800 sec				

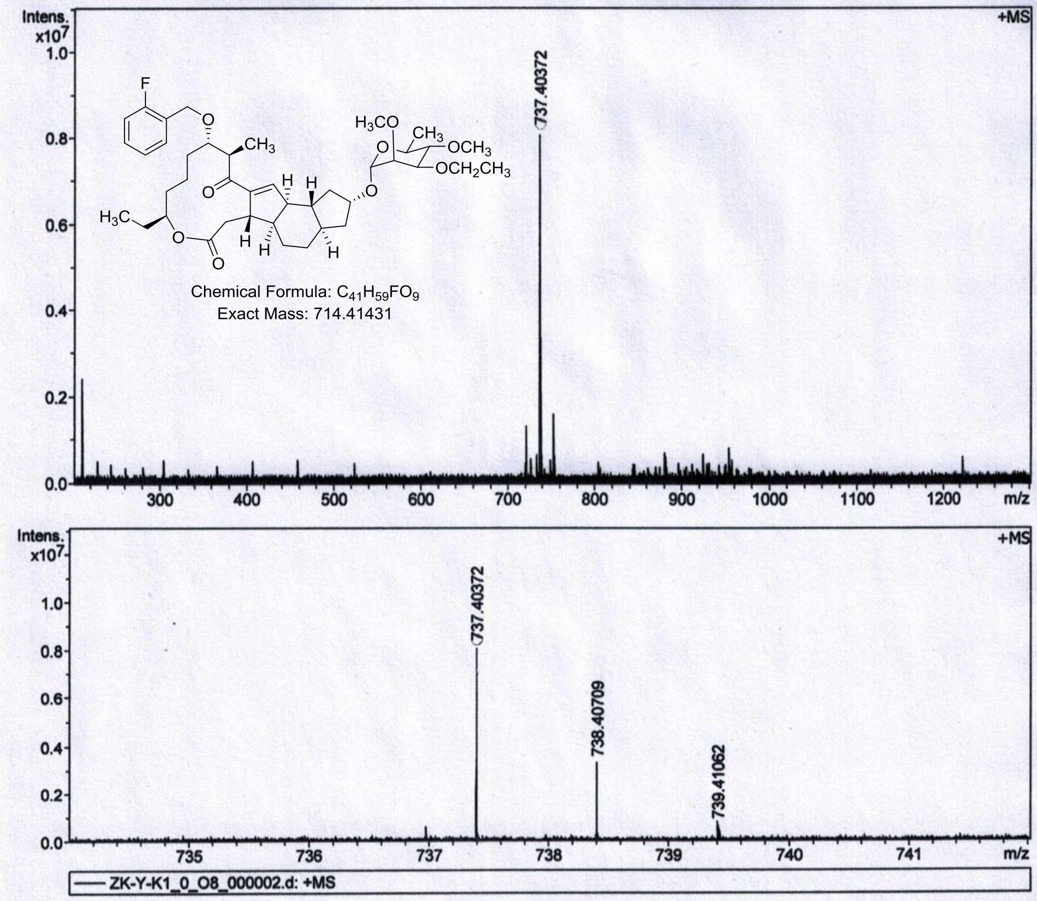

Meas. m/z	#	Ion Formula	Score	m/z	err [ppm]	Mean err [ppm]	mSigma	rdb	e⁻ Conf	N-Rule
737.403716	1	C41H59FNaO9	100.00	737.403532	-0.2	-0.3	25.9	11.5	even	ok

MS of **7h**

Acquisition Parameter

Acquisition Mode	Single MS	Acquired Scans	4	Calibration Date	Thu Jun 16 04:54:23
Polarity	Positive	No. of Cell Fills	1	Data Acquisition Size	[illegible]
Broadband Low Mass	202.1 m/z	No. of Laser Shots	10	Data Processing Size	4194304
Broadband High Mass	2000.0 m/z	Laser Power	32.2 lp	Apodization	Sine-Bell Multiplication
Source Accumulation	0.001 sec	Laser Shot Frequency	0.020 sec		
Ion Accumulation Time	0.800 sec				

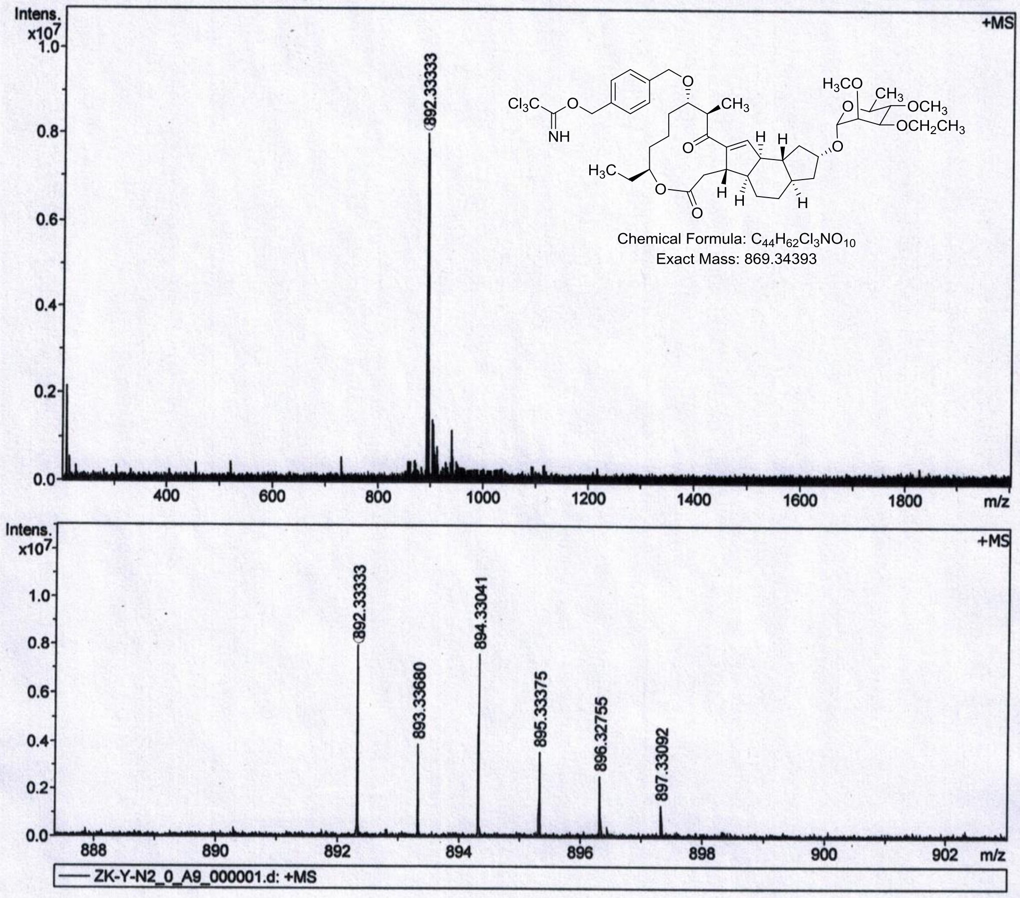

Meas. m/z	#	Ion Formula	Score	m/z	err [ppm]	Mean err [ppm]	mSigma	rdb	e⁻ Conf	N-Rule
892.333331	1	C44H62Cl3NNaO10	100.00	892.333151	-0.2	0.6	53.3	12.5	even	ok

MS of **7i**

Acquisition Parameter

Acquisition Mode	Single MS	Acquired Scans	5	Calibration Date	Fri Jun 3 05:53:25 2016
Polarity	Positive	No. of Cell Fills	1	Data Acquisition Size	2097152
Broadband Low Mass	202.1 m/z	No. of Laser Shots	10	Data Processing Size	4194304
Broadband High Mass	1300.0 m/z	Laser Power	28.2 lp	Apodization	Sine-Bell Multiplication
Source Accumulation	0.001 sec	Laser Shot Frequency	0.020 sec		
Ion Accumulation Time	0.800 sec				

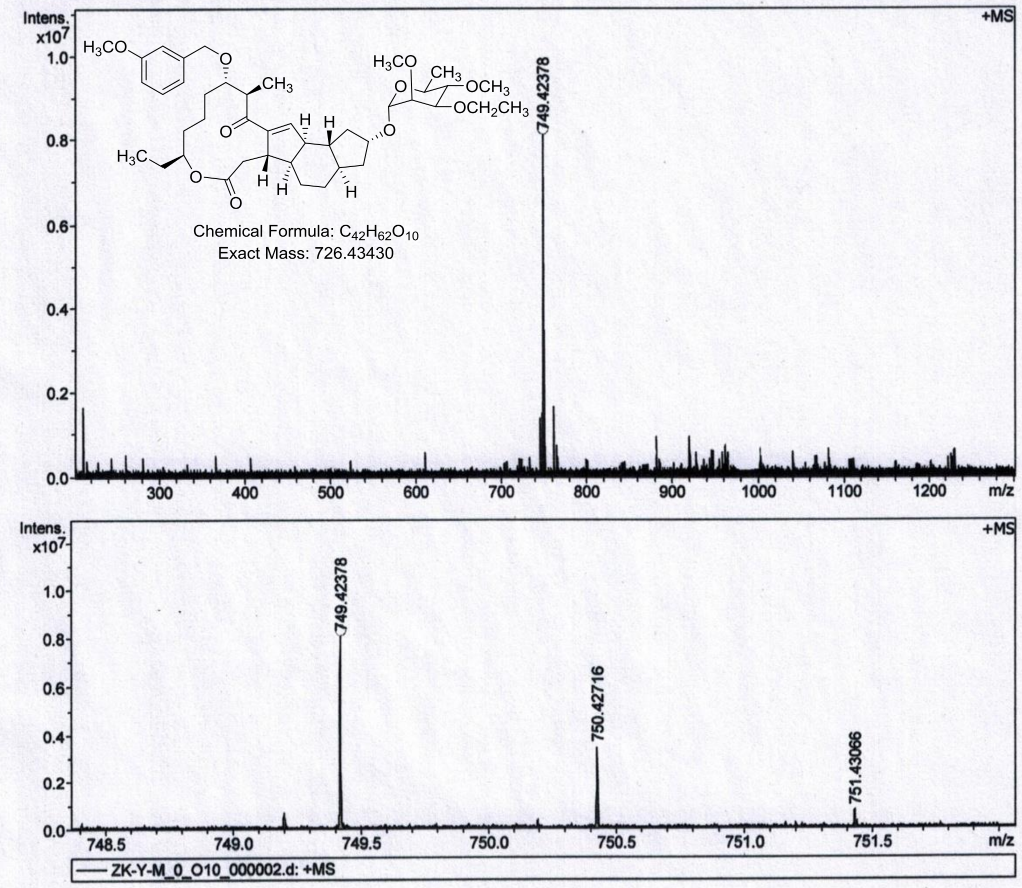

Meas. m/z	#	Ion Formula	Score	m/z	err [ppm]	Mean err [ppm]	mSigma	rdb	e⁻ Conf	N-Rule
749.423777	1	C42H62NaO10	100.00	749.423519	0.3	-0.4	23.0	11.5	even	ok

MS of **7j**

Acquisition Parameter

Acquisition Mode	Single MS	Acquired Scans	2	Calibration Date	Tue Nov 22 04:41:09
Polarity	Positive	No. of Cell Fills	1	Data Acquisition Size	2096152
Broadband Low Mass	202.1 m/z	No. of Laser Shots	10	Data Processing Size	4194304
Broadband High Mass	2400.0 m/z	Laser Power	41.4 lp	Apodization	Sine-Bell Multiplication
Source Accumulation	0.001 sec	Laser Shot Frequency	0.020 sec		
Ion Accumulation Time	0.300 sec				

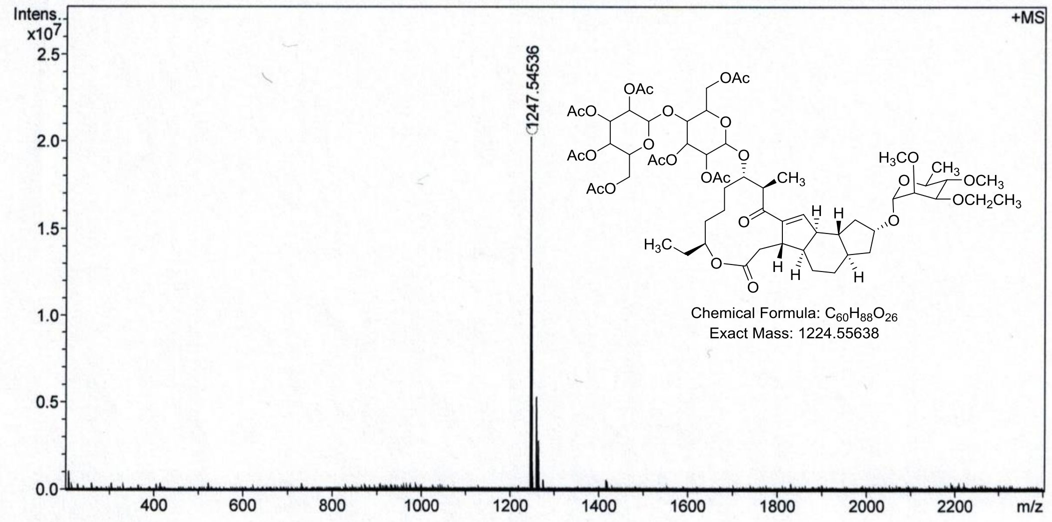

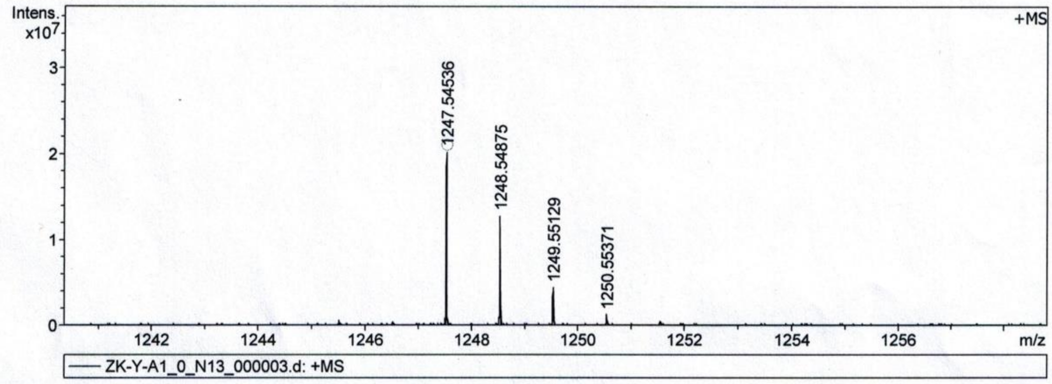

Meas. m/z	#	Ion Formula	Score	m/z	err [ppm]	Mean err [ppm]	mSigma	rdb	e⁻ Conf	N-Rule
1247.545361	1	C60H88NaO26	100.00	1247.545604	0.2	0.3	27.5	16.5	even	ok

MS of **7k**

Acquisition Parameter

Acquisition Mode	Single MS	Acquired Scans	4	Calibration Date	Fri Dec 9 06:41:47 2016
Polarity	Positive	No. of Cell Fills	1	Data Acquisition Size	2097152
Broadband Low Mass	202.1 m/z	No. of Laser Shots	10	Data Processing Size	4194304
Broadband High Mass	1500.0 m/z	Laser Power	34.6 lp	Apodization	Sine-Bell Multiplication
Source Accumulation	0.001 sec	Laser Shot Frequency	0.020 sec		
Ion Accumulation Time	0.300 sec				

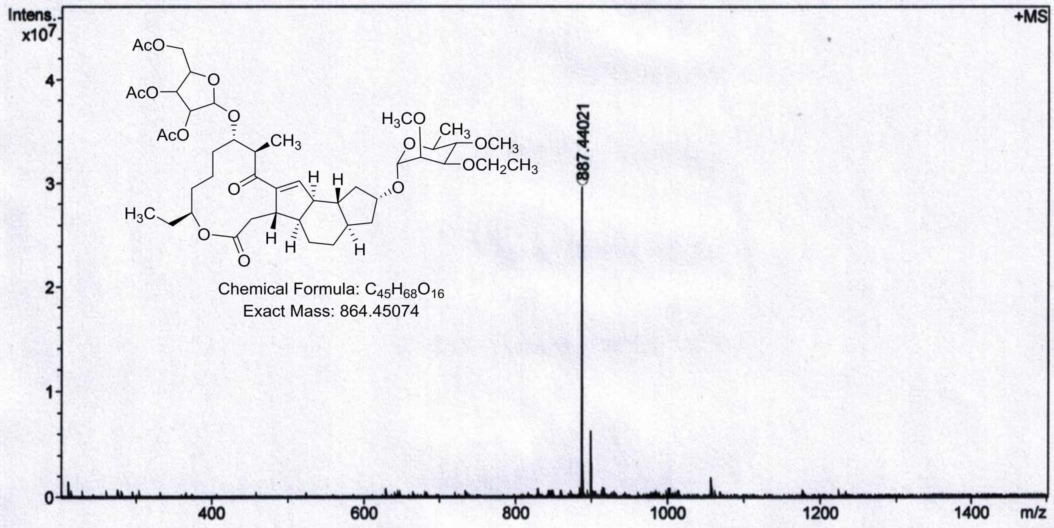

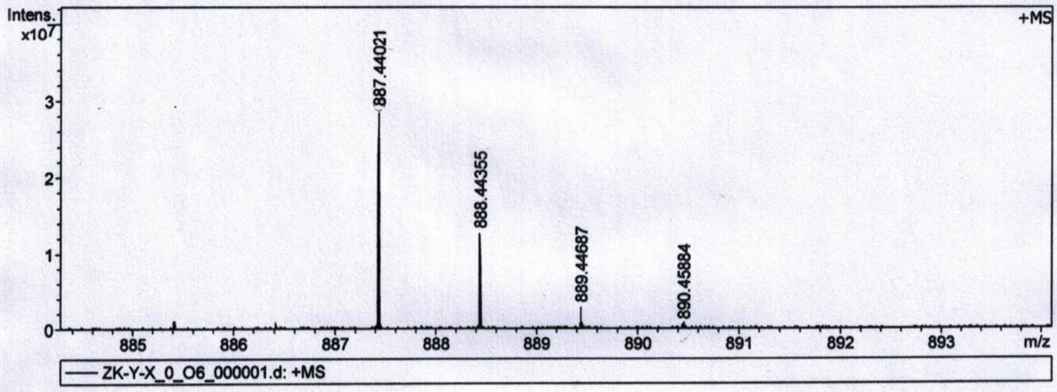

Meas. m/z	#	Ion Formula	Score	m/z	err [ppm]	Mean err [ppm]	mSigma	rdb	e⁻ Conf	N-Rule
887.440211	1	C45H68NaO16	100.00	887.439957	-0.3	-0.3	21.5	11.5	even	ok

MS of **7l**

Acquisition Parameter

Acquisition Mode	Single MS	Acquired Scans	5	Calibration Date	Fri Dec 23 05:39:24 2016
Polarity	Positive	No. of Cell Fills	1	Data Acquisition Size	2097152
Broadband Low Mass	202.1 m/z	No. of Laser Shots	10	Data Processing Size	4194304
Broadband High Mass	1600.0 m/z	Laser Power	35.4 lp	Apodization	Sine-Bell Multiplication
Source Accumulation	0.001 sec	Laser Shot Frequency	0.020 sec		
Ion Accumulation Time	1.000 sec				

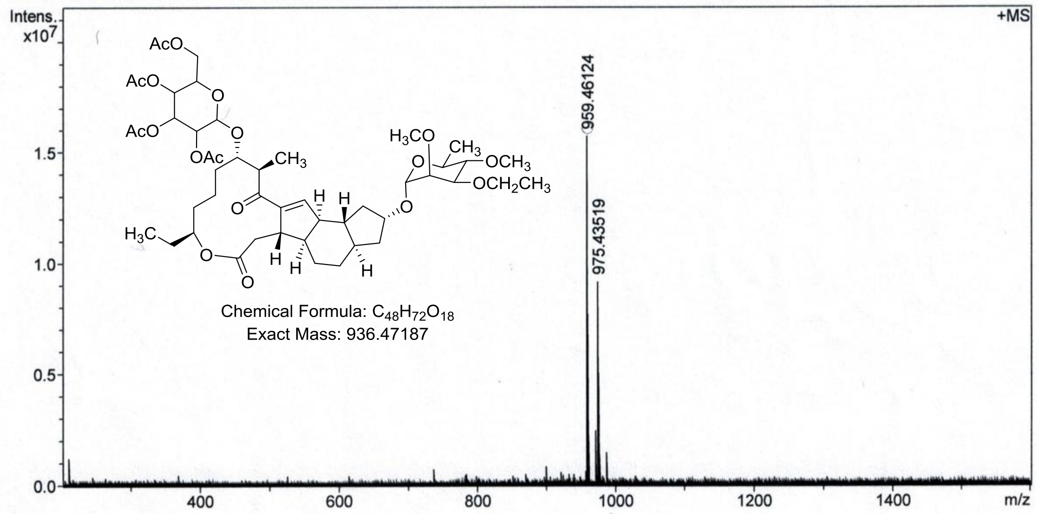

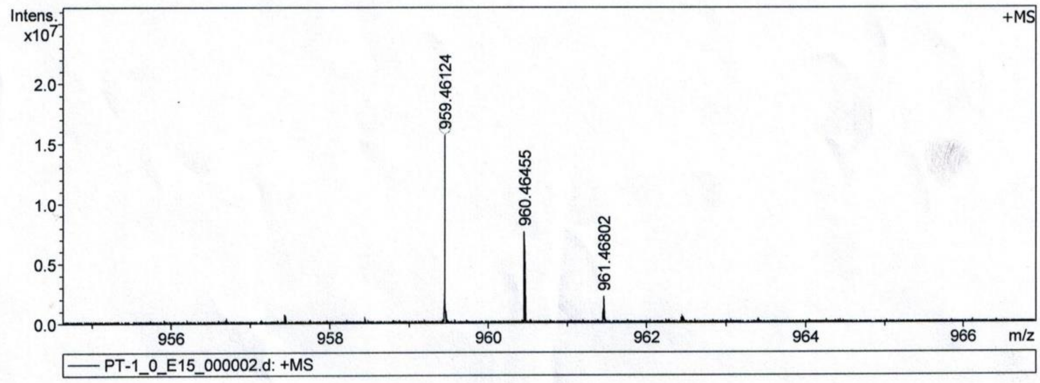

Meas. m/z	#	Ion Formula	Score	m/z	err [ppm]	Mean err [ppm]	mSigma	rdb	e⁻ Conf	N-Rule
959.461240	1	C48H72NaO18	100.00	959.461086	0.2	-0.2	25.2	12.5	even	ok

MS of **8a**

Acquisition Parameter

Acquisition Mode	Single MS	Acquired Scans	4	Calibration Date	Fri Jun 3 05:53:25 2016
Polarity	Positive	No. of Cell Fills	1	Data Acquisition Size	2097152
Broadband Low Mass	202.1 m/z	No. of Laser Shots	10	Data Processing Size	4194304
Broadband High Mass	1300.0 m/z	Laser Power	28.2 lp	Apodization	Sine-Bell Multiplication
Source Accumulation	0.001 sec	Laser Shot Frequency	0.020 sec		
Ion Accumulation Time	0.800 sec				

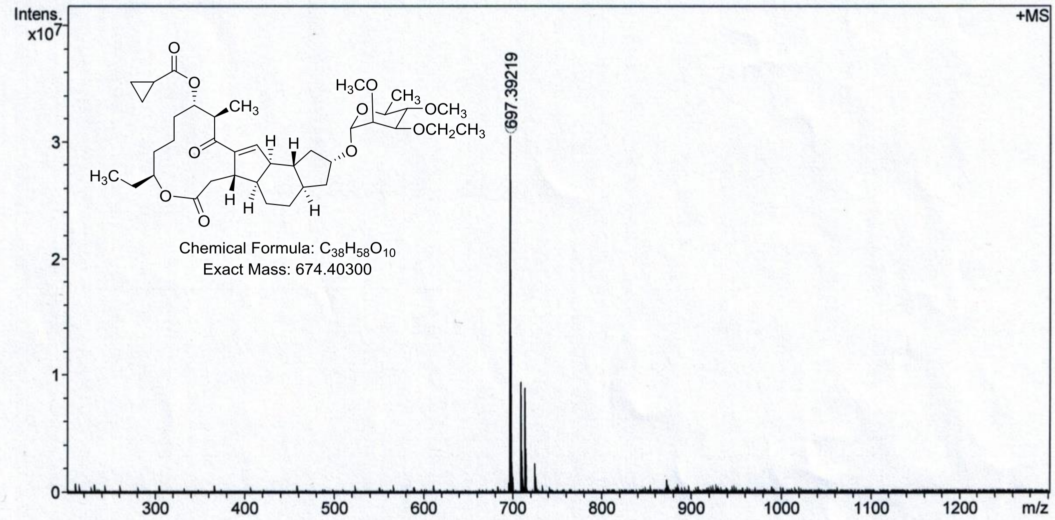

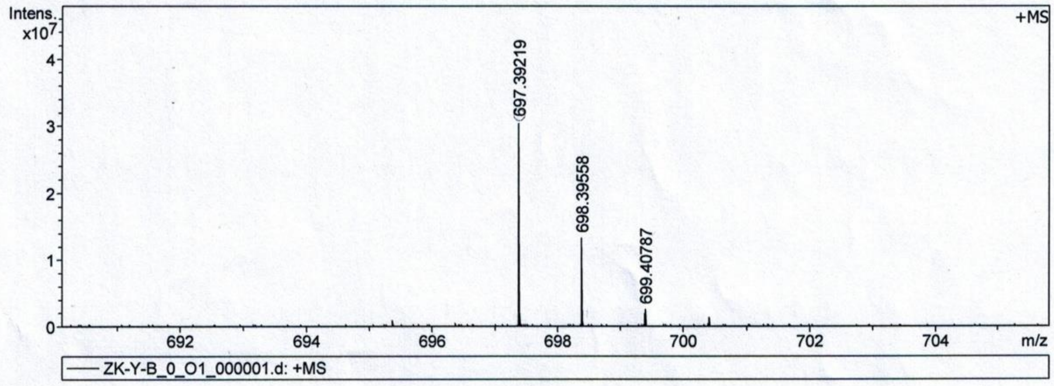

Meas. m/z	#	Ion Formula	Score	m/z	err [ppm]	Mean err [ppm]	mSigma	rdb	e⁻ Conf	N-Rule
697.392189	1	C38H58NaO10	100.00	697.392219	-0.0	0.0	55.0	9.5	even	ok

MS of **8b**

Acquisition Parameter

Acquisition Mode	Single MS	Acquired Scans	3	Calibration Date	Fri Jun 3 05:53:25 2016
Polarity	Positive	No. of Cell Fills	1	Data Acquisition Size	2097152
Broadband Low Mass	202.1 m/z	No. of Laser Shots	10	Data Processing Size	4194304
Broadband High Mass	1300.0 m/z	Laser Power	28.2 lp	Apodization	Sine-Bell Multiplication
Source Accumulation	0.001 sec	Laser Shot Frequency	0.020 sec		
Ion Accumulation Time	0.800 sec				

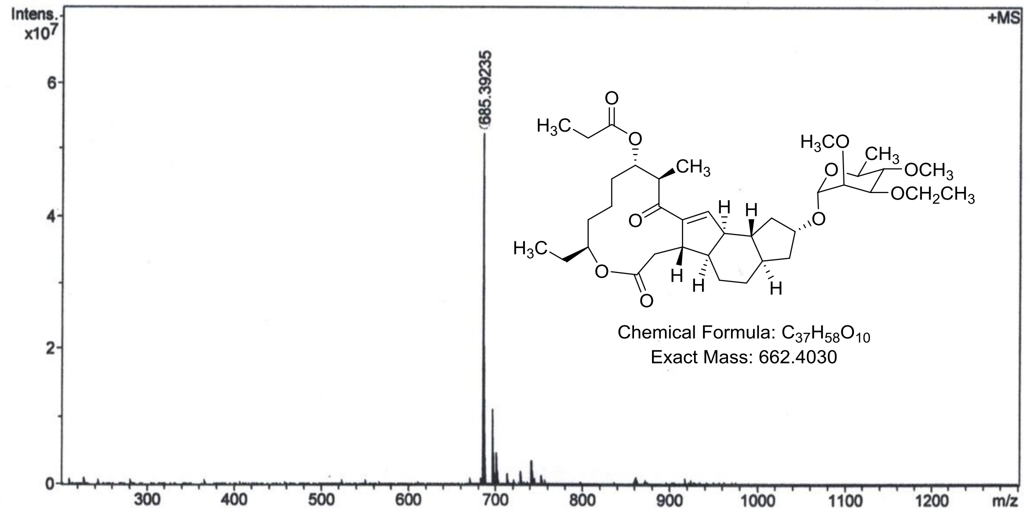

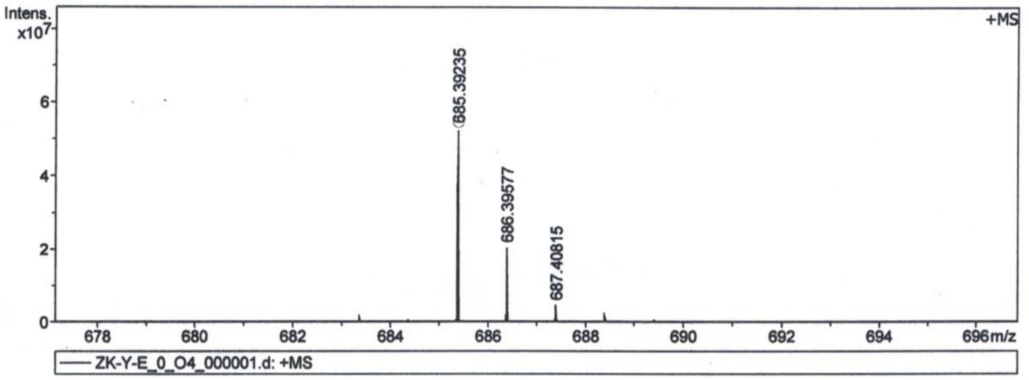

Meas. m/z	#	Ion Formula	Score	m/z	err [ppm]	Mean err [ppm]	mSigma	rdb	e⁻ Conf	N-Rule
685.392352	1	C37H58NaO10	100.00	685.392219	0.2	-0.2	53.2	8.5	even	ok

MS of **8c**

Acquisition Parameter

Acquisition Mode	Single MS	Acquired Scans	3			Calibration Date	Fri Jun 3 05:53:25 2016
Polarity	Positive	No. of Cell Fills	1			Data Acquisition Size	2097152
Broadband Low Mass	202.1 m/z	No. of Laser Shots	10			Data Processing Size	4194304
Broadband High Mass	1300.0 m/z	Laser Power	28.2 lp			Apodization	Sine-Bell Multiplication
Source Accumulation	0.001 sec	Laser Shot Frequency	0.020 sec				
Ion Accumulation Time	0.800 sec						

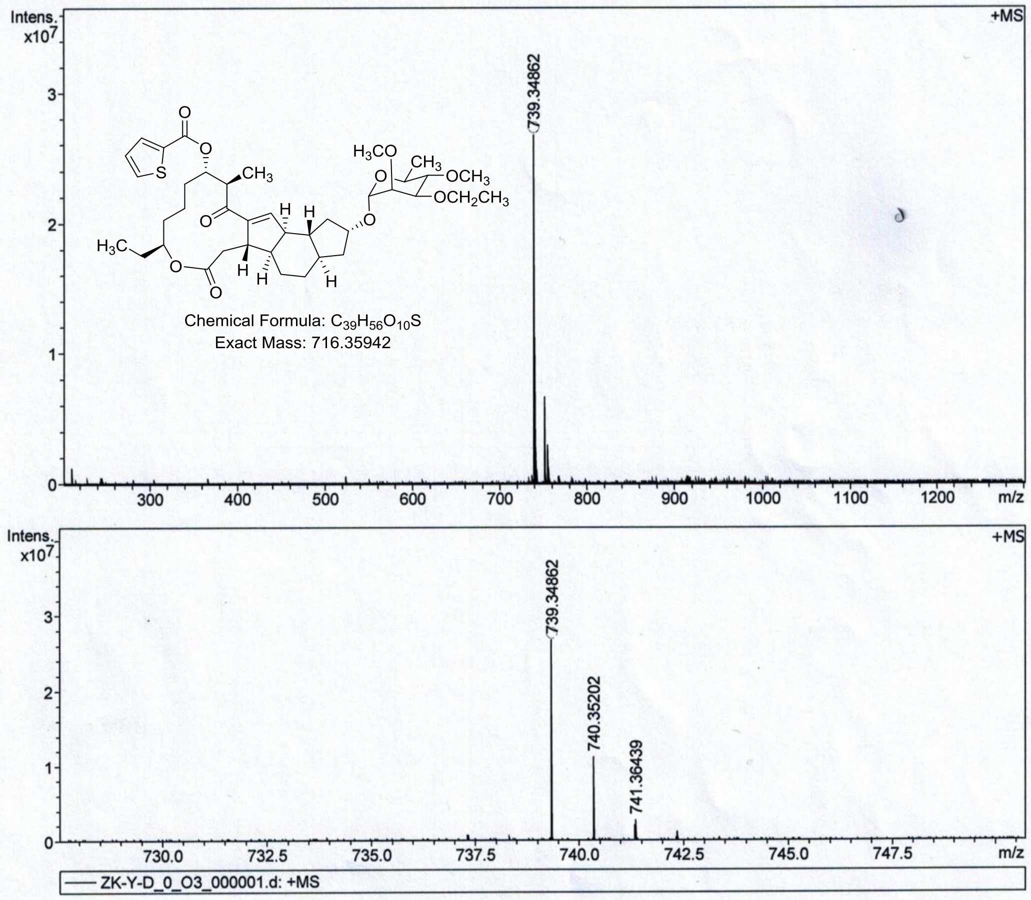

Meas. m/z	#	Ion Formula	Score	m/z	err [ppm]	Mean err [ppm]	mSigma	rdb	e⁻ Conf	N-Rule
739.348619	1	C39H56NaO10S	100.00	739.348640	0.0	-0.0	74.5	11.5	even	ok

MS of **8d**

Acquisition Parameter

Acquisition Mode	Single MS	Acquired Scans	4	Calibration Date	Fri Jun 3 05:53:25 2016
Polarity	Positive	No. of Cell Fills	1	Data Acquisition Size	2097152
Broadband Low Mass	202.1 m/z	No. of Laser Shots	10	Data Processing Size	4194304
Broadband High Mass	1300.0 m/z	Laser Power	28.2 lp	Apodization	Sine-Bell Multiplication
Source Accumulation	0.001 sec	Laser Shot Frequency	0.020 sec		
Ion Accumulation Time	0.800 sec				

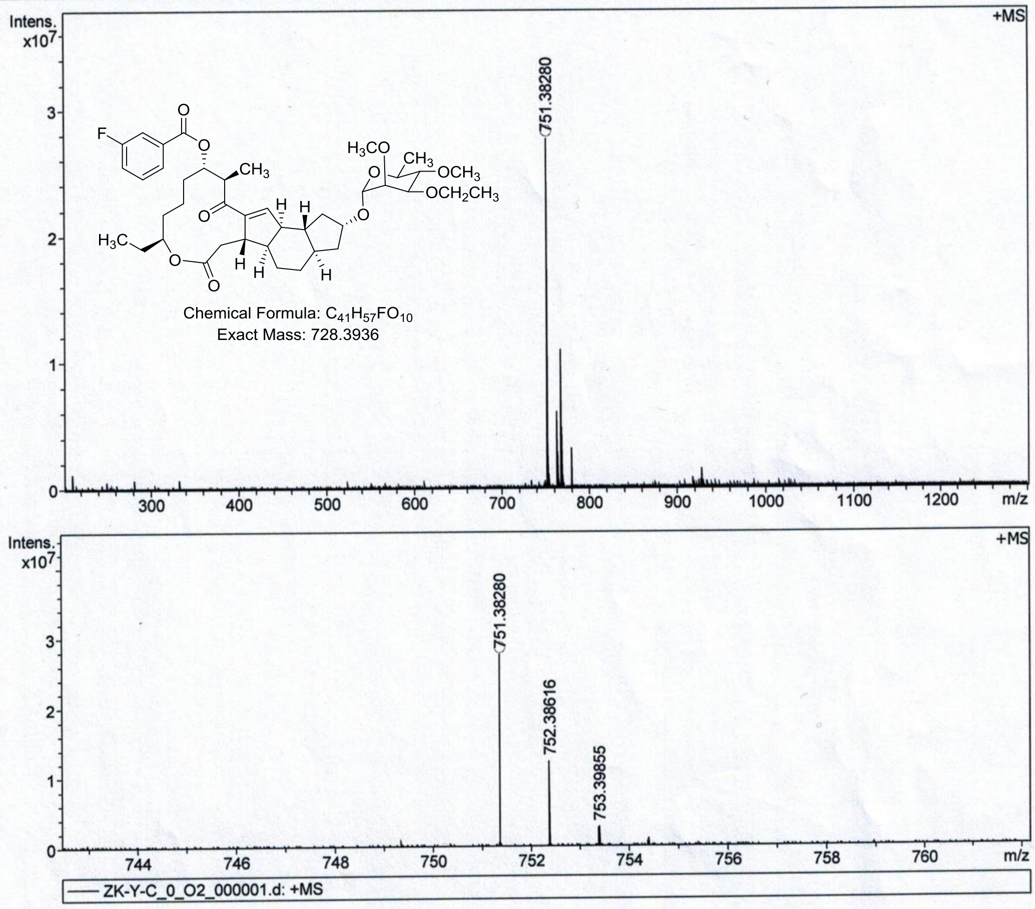

Meas. m/z	#	Ion Formula	Score	m/z	err [ppm]	Mean err [ppm]	mSigma	rdb	e⁻ Conf	N-Rule
751.382804	1	C41H57FNaO10	100.00	751.382797	0.0	0.0	55.3	12.5	even	ok

MS of **8e**

Acquisition Parameter

Acquisition Mode	Single MS	Acquired Scans	2	Calibration Date	Wed Apr 4 05:35:24 2018
Polarity	Positive	No. of Cell Fills	1	Data Acquisition Size	2097152
Broadband Low Mass	101.1 m/z	No. of Laser Shots	10	Data Processing Size	4194304
Broadband High Mass	1400.0 m/z	Laser Power	26.0 lp	Apodization	Sine-Bell Multiplication
Source Accumulation	0.001 sec	Laser Shot Frequency	0.020 sec		
Ion Accumulation Time	0.300 sec				

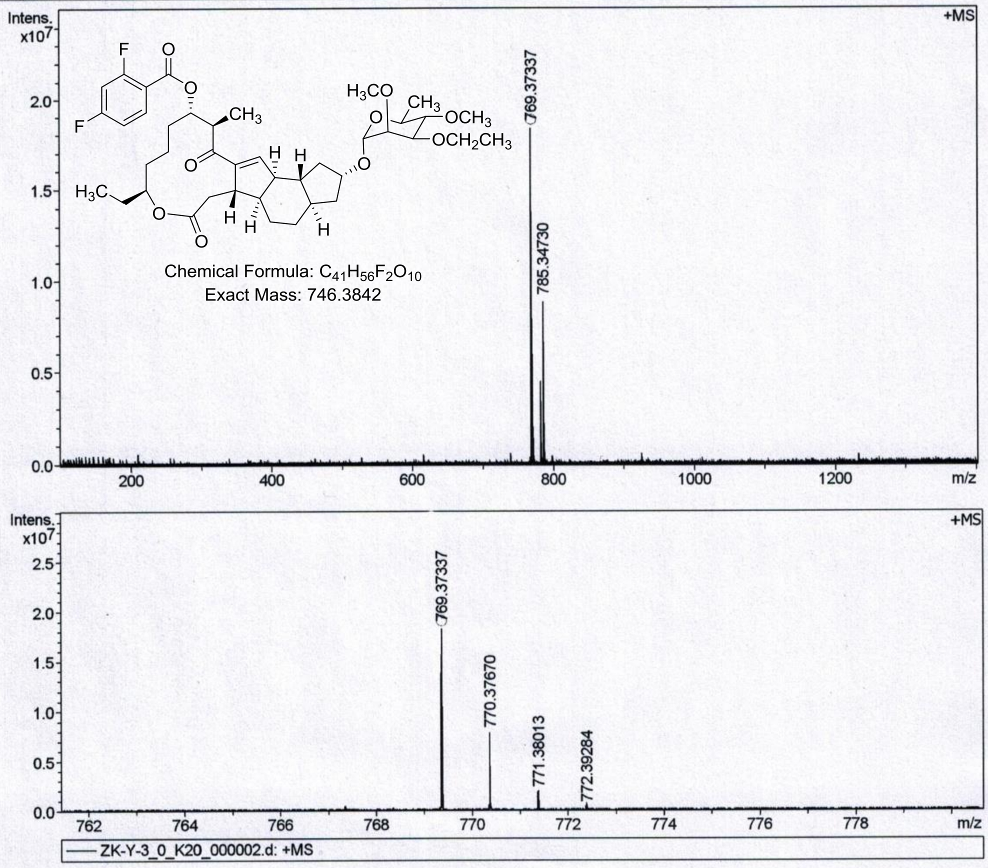

Meas. m/z	#	Ion Formula	Score	m/z	err [ppm]	Mean err [ppm]	mSigma	rdb	e⁻ Conf	N-Rule
769.373368	1	C41H56F2NaO10	100.00	769.373375	-0.0	0.0	17.9	12.5	even	ok

MS of **8f**

Acquisition Parameter

Acquisition Mode	Single MS	Acquired Scans	6	Calibration Date	Wed Apr 4 05:35:24 2018
Polarity	Positive	No. of Cell Fills	1	Data Acquisition Size	2097152
Broadband Low Mass	101.1 m/z	No. of Laser Shots	10	Data Processing Size	4194304
Broadband High Mass	1400.0 m/z	Laser Power	26.0 lp	Apodization	Sine-Bell Multiplication
Source Accumulation	0.001 sec	Laser Shot Frequency	0.020 sec		
Ion Accumulation Time	0.300 sec				

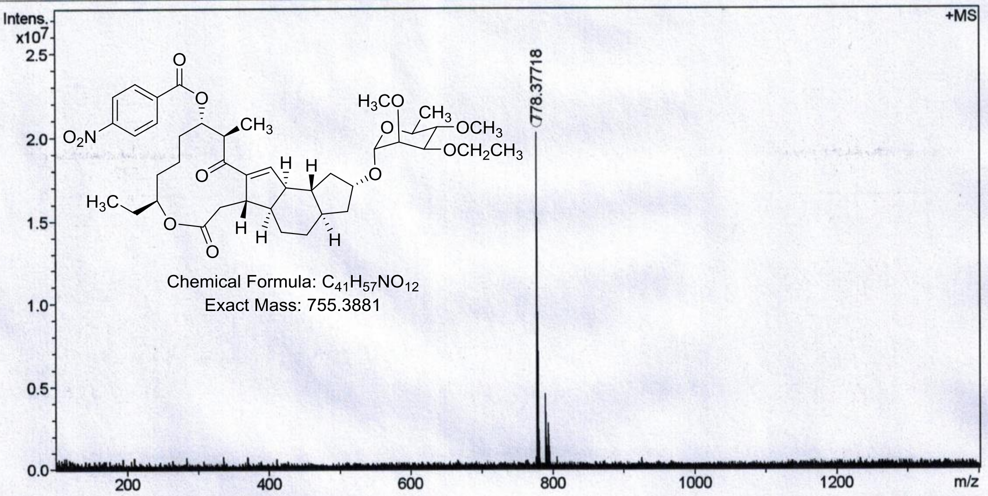

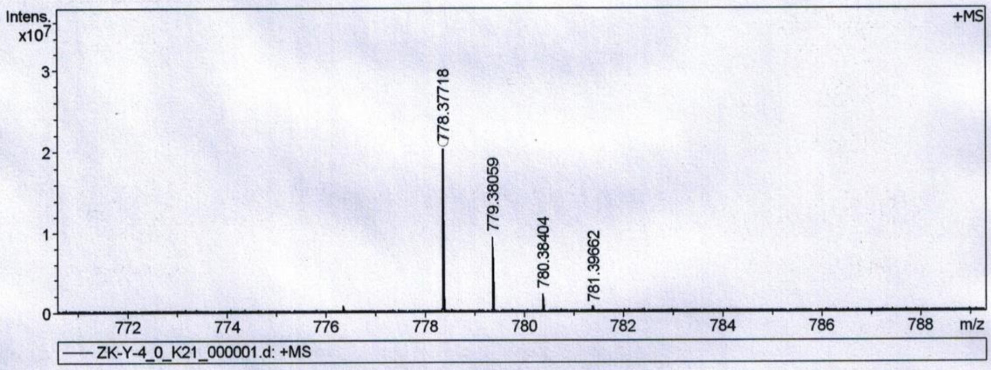

Meas. m/z	#	Ion Formula	Score	m/z	err [ppm]	Mean err [ppm]	mSigma	rdb	e⁻ Conf	N-Rule
778.377181	1	C41H57NNaO12	100.00	778.377297	0.1	0.1	14.3	13.5	even	ok

MS of **8g**

Acquisition Parameter

Acquisition Mode	Single MS	Acquired Scans	5	Calibration Date	Wed Apr 4 05:35:24 2018
Polarity	Positive	No. of Cell Fills	1	Data Acquisition Size	2097152
Broadband Low Mass	101.1 m/z	No. of Laser Shots	10	Data Processing Size	4194304
Broadband High Mass	1400.0 m/z	Laser Power	27.0 lp	Apodization	Sine-Bell Multiplication
Source Accumulation	0.001 sec	Laser Shot Frequency	0.020 sec		
Ion Accumulation Time	0.300 sec				

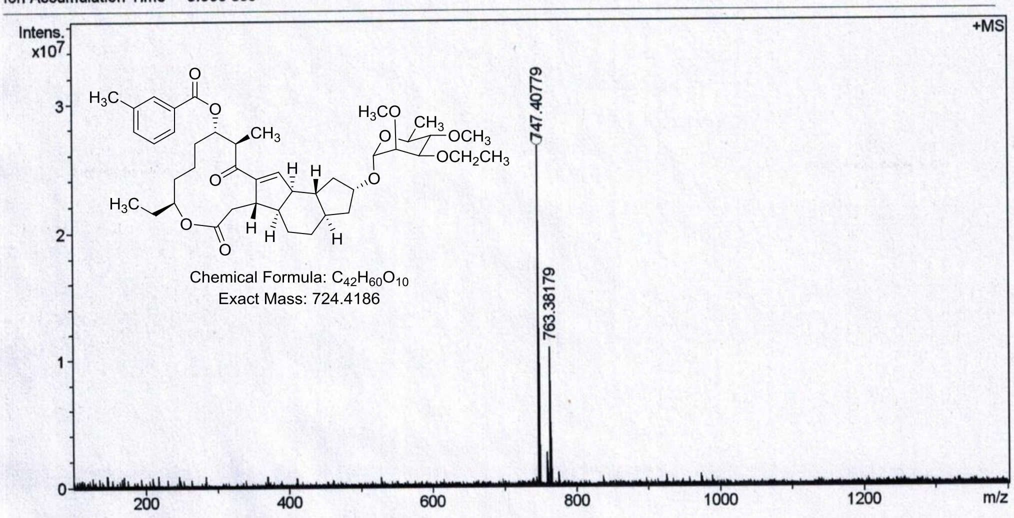

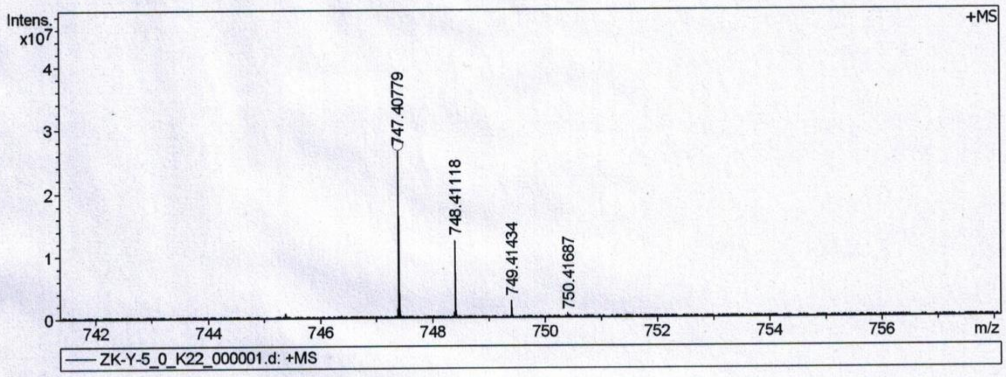

Meas. m/z	#	Ion Formula	Score	m/z	err [ppm]	Mean err [ppm]	mSigma	rdb	e⁻ Conf	N-Rule
747.407790	1	C42H60NaO10	100.00	747.407869	0.1	0.1	11.1	12.5	even	ok

MS of **8h**

Acquisition Parameter

Acquisition Mode	Single MS	Acquired Scans	11	Calibration Date	Wed Apr 4 05:35:24 2018
Polarity	Positive	No. of Cell Fills	1	Data Acquisition Size	2097152
Broadband Low Mass	101.1 m/z	No. of Laser Shots	10	Data Processing Size	4194304
Broadband High Mass	1400.0 m/z	Laser Power	27.0 lp	Apodization	Sine-Bell Multiplication
Source Accumulation	0.001 sec	Laser Shot Frequency	0.020 sec		
Ion Accumulation Time	0.300 sec				

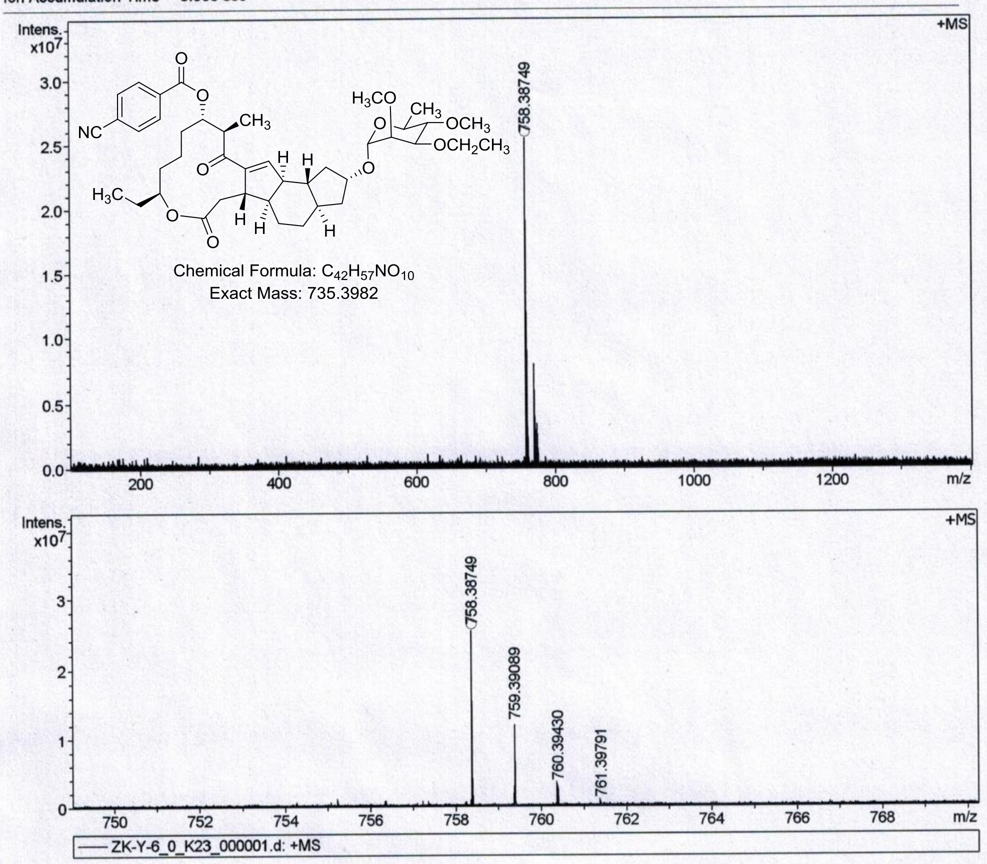

Meas. m/z	#	Ion Formula	Score	m/z	err [ppm]	Mean err [ppm]	mSigma	rdb	e⁻ Conf	N-Rule
758.387492	1	C42H57NNaO10	100.00	758.387468	0.0	-0.1	3.2	14.5	even	ok

MS of **8i**

Acquisition Parameter

Acquisition Mode	Single MS	Acquired Scans	7	Calibration Date	Wed Apr 4 05:35:24 2018
Polarity	Positive	No. of Cell Fills	1	Data Acquisition Size	2097152
Broadband Low Mass	101.1 m/z	No. of Laser Shots	10	Data Processing Size	4194304
Broadband High Mass	1400.0 m/z	Laser Power	31.4 lp	Apodization	Sine-Bell Multiplication
Source Accumulation	0.001 sec	Laser Shot Frequency	0.020 sec		
Ion Accumulation Time	0.300 sec				

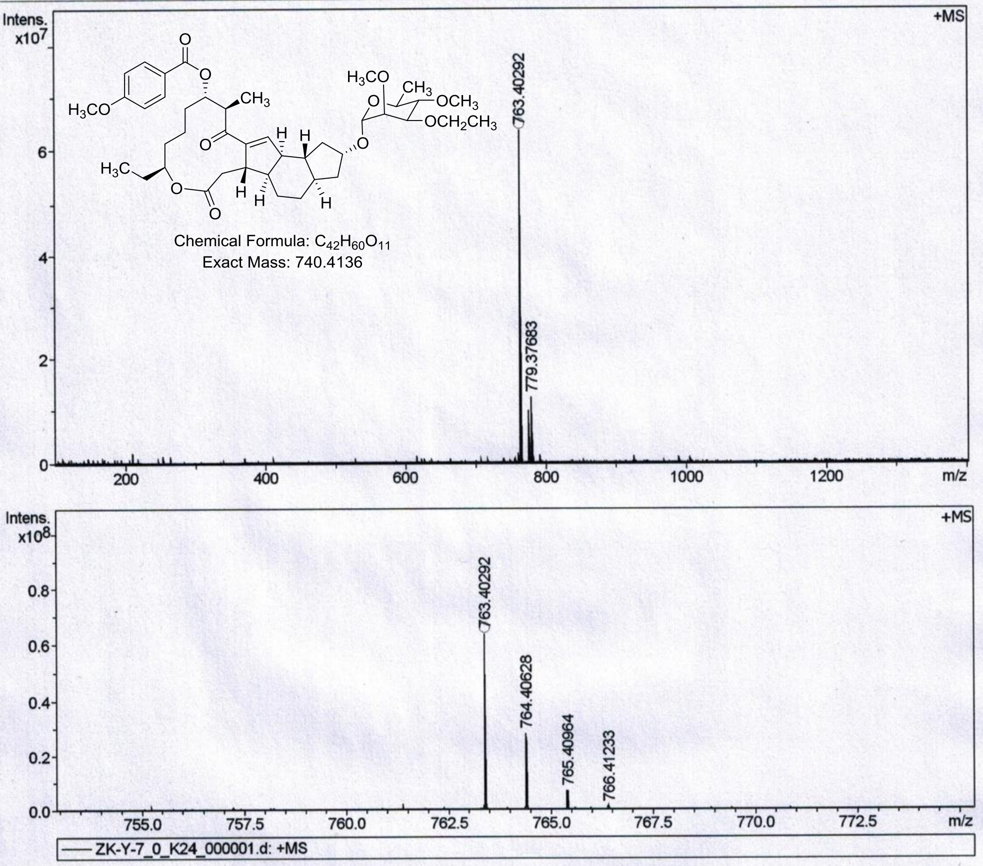

Meas. m/z	#	Ion Formula	Score	m/z	err [ppm]	Mean err [ppm]	mSigma	rdb	e⁻ Conf	N-Rule
763.402919	1	C42H60NaO11	100.00	763.402783	0.2	-0.2	14.2	12.5	even	ok
